# Rapid assessment of conformational preferences in biaryl and aryl carbonyl fragments

**DOI:** 10.1371/journal.pone.0192974

**Published:** 2018-03-14

**Authors:** Sonia Maria Gutiérrez Sanfeliciano, John M. Schaus

**Affiliations:** 1 Centro de Investigación Lilly S.A., Alcobendas, Madrid, Spain; 2 Discovery Chemistry Research and Technologies, Lilly Research Laboratories, Eli Lilly and Company, Indianapolis, Indiana, United States of America; Universidade Nova de Lisboa Instituto de Tecnologia Quimica e Biologica, PORTUGAL

## Abstract

The ability to rapidly assess the preferred conformation of key fragments in a structure “by visual inspection” is a very useful starting point in the process of drug design. With the ability to do so, one could address questions like: “How could we avoid planarity in a molecule?”, “Will a molecule change its conformational preference if we make it more or less basic?” or “How does this electronic repulsion affect the conformational preference in the system?” in timely fashion. In this paper, we describe how the conformational energy profile (CEP, plot of energy as a function of dihedral bond angle) of a fragment can be interpreted through the understanding the interplay between resonance stabilization, steric effects and electrostatic interactions. Fifty-nine biaryl and aryl carbonyl fragments present in oral drugs or which are close derivatives thereof were selected. Calculation of their CEPs using *ab initio* methodology allowed us to conclude the relative importance of these factors in the conformational preference of these fragments as follows: “steric repulsion > lone pair—lone pair repulsion > lone pair—fluorine repulsion > resonance stabilization” and to formulate “rules of thumb” that the practicing medicinal/organic chemist can apply when analysing molecules that contain these fragments.

## Introduction

Understanding a molecule’s conformational preference [1–3] is critical in the practice of medicinal chemistry [4]. Factors that affect a molecule’s biological activity, such as the shape, physicochemical properties and the position of key functional groups, are highly dependent on its conformation. [5] Computational methods (molecular mechanics, semi-empirical or *ab initio*) are often employed to identify low energy conformations and to guide compound design to optimize biological activity [6–7]. While information helpful to the medicinal chemist is obtained from these studies, they are often time-consuming and may require specialized software, knowledge and training. The development of a qualitative method that allows the medicinal chemist to do a rapid conformational assessment “by visual inspection” of key fragments in a structure would save time during compound design and brainstorming discussions. More rigorous and time-consuming molecular modeling studies could subsequently be conducted to evaluate and refine the ideas coming from this qualitative assessment.

One method for performing such a rapid assessment of conformation would be to dissect the molecule of interest into a number of fragments. Determination of the lowest energy conformation or conformations of each fragment followed by reconstitution of the molecule using the resulting energy-minimized fragments would provide a good idea of the conformation of the molecule of interest. In order to perform this evaluation, one needs to have a high quality energetic torsional profile of each fragment. Fortunately, several groups have described studies of molecular fragments commonly found in drug-like molecules. Their findings allowed us to conclude that DFT/B3LYP with 6-31G* as the basis set would be generally be sufficient to evaluate the selected fragments in an efficient way. We later confirmed this with some additional high theory calculations on a subset of these fragments.

Hao, *et al* [8] analyzed the conformations of common torsional motifs of small molecules extracted from crystal structures of protein-ligand complexes. Due to the limited number of complexes available, only twenty-one fragments were examined and compared with their torsional potentials. They used *ab initio* DFT/B3LYP calculations and a 6–311++G** basis set in a torsion-coordinate scan procedure applied to the majority of motifs that they described. They concluded that the most probable conformations of the torsion motifs agreed well with the calculated global energy minima. Importantly, they showed that greater than 95% of the conformational torsions found in the crystal structures occurred in the energy region less than 4 kcal/mol above the global minimum. Based on the strength of their studies, we have used this 4 kcal/mol threshold to judge which conformations are likely to be energetically relevant.

Chein and Corey [9] later used the same method, DFT/B3LYP, but with smaller 6-31G* basis set, to study a series of heteroaromatic ethers and 2-(2-pyridyl)pyridine. They concluded that the conformations of these molecules were determined by electron repulsion between in-plane nonbonding electron lone pairs.

In an effort to determine highly accurate values for parameterization of the OPLS force field, Jorgensen, *et al*. [10] analyzed the torsional energetics for thirty-three biaryl molecules using *ab initio* MP2-(full)/6-311G(d,p) optimizations followed by single point calculations at the MP2(full)/aug-cc-pVTZ level. In agreement with Hao’s group [8] and Chein and Corey [9], their systems showed a planar or non-planar geometry preference based on the prevalence of steric and electrostatic repulsions.

These papers demonstrate that *ab initio* calculations can be used to correctly determine rotational preferences of fragments commonly found in drug or drug-like molecules. Unfortunately, only a relatively small number of fragments had already been studied. As a result, the conformational preferences of many other commonly used fragments have not been calculated and the relative importance of the steric, resonance and electronic factors that determine their conformational preferences hasn’t been explicitly reported. Here we report an expansion of these studies including 6, 6-biaryls, 5, 6-biaryls and aryl carbonyl fragments as structural motifs commonly used in drug discovery. Rotation about the bond linking the two aromatic rings or the aryl-carbonyl motif is graphically presented (CEP: Conformational Energy Profile- a plot of energy (kcal/mol) as a function of bond angle (degrees)) and it gives rise to a series of rotamers of varying energy that has allowed us to study the effect of each one of these factors individually:

- Resonance stabilization between aromatic rings or within aryl-carbonyl fragments which favors planar conformations.
- Steric effects between the *ortho* substituents on biaryl or aryl-carbonyl fragments, which disfavors planar conformations.
- Electrostatic interactions (lone pair—lone pair and lone pair—pi electron cloud interactions) which disfavor conformations in which a lone pair is in proximity to either another lone pair or a pi cloud. And also a brief discussion about how the interaction of a polar hydrogen-pi cloud favors the proximity of the polar hydrogen and the pi cloud. and to develop “rules of thumb” that a medicinal chemist can use during a drug design process to do a rapid conformational assessment.

As crystallographic information [11] has been widely used in the literature [12–13] to determine the conformational preferences of small molecules, we looked for the existence of the studied fragments as part of a final molecule in the Crystallographic Cambridge Database (CSD) [14] (ConQuest, Relibase) [15]. We measured the rotation angle about the bond linking the two aromatic rings or the aryl-carbonyl motif, and confirmed that torsions that occurred in the energy region higher than 4 kcal/mol above the global minimum were mostly not found in the crystal structures as Hao et al. [8] had published previously. Positive Predictive Values (PPV) and Specificity statistical parameters were calculated and showed a significant correlation between experimental non-observance of any rotamer in a defined interval of rotation and the condition that all rotamers in this torsional interval had an energy higher than 4 kcal/mol.

## Method

Torsional energy profiles were calculated using *Jaguar* (DFT) versions (7.7–8.1, Maestro, Schrodinger Software) [16] in the gas phase. The results between versions were consistent for those cases where more than one version was tested. Geometries of the fragments were optimized at various fixed aryl-aryl or aryl-carbonyl dihedral angles, using relaxed dihedral scans at the B3LYP/ 6-31G* level for most systems in 20° increments and the rotation was performed from 0° to 180°. B3LYP/lacvp* was used for biphenyl fragments containing heavy halogens (25, 26). The dihedral angle being studied in each of the fragments is indicated with stars, with the orientation as drawn defining 0°. This level of theory has been reported previously as being sufficient for our purpose [9].

The resulting energies were plotted as a function of the dihedral angle, curve fitting these points provided the conformational energy profile (CEP). Because all fragments studied showed C2 symmetry with respect to the bond of rotation, the CEP of a fragment from 0° to 180° is identical to that from 0° to -180°. CEPs of related fragments are shown on the same graph in order to make it easier to follow the discussion. Individual graphs can be built from the tabulated data provided in the Supporting Information (S1 Table).

Positive Predictive Values (PPV) and Specificity statistical parameters were calculated with the data found in the CSD searches, by defining a “positive observation” as the non-existence of a rotamer in the defined torsional interval where ΔE > 4 kcal/mol over ground state while, the defined torsional interval within which all rotamers had ΔE > 4 kcal/mol over ground state (and therefore should not be observed) was”predicted to non-exist” is a “positive prediction”. These binary statistical parameters are based on total number of true positives and false positives [17]. We could not adequately calculate the NPV (Negative Predictive Value) or Sensitivity since these require true negative and false negative values. A false negative would be defined as a rotamer that was predicted to exist but wasn’t found in the CSD. There are many reasons why a rotamer may not be found in the CSD apart from energetic inaccessibility (principally because the compound was never prepared or was prepared but the crystal structure never determined.) Detailed information about how the searches were done, tables with the torsional angle, frequency of appearance of fragments in available X-ray structures in ConQuest and Relibase and the statistical analysis of the collected data are provided in the Supporting information (S1 File).

A comparison with CEPs of fragments 1, 5, 10, 24, 41, 51 and 57 reported using different higher level basis sets [1, 8, 10] showed that in all cases the energy differences were within 1 kcal/mol of the 6-31G* values and the minimum and maximum energy rotamers were located at the same torsional angles. We performed calculations on fragments 1, 4–7, 15, 23–26, 31, 42, 44–47, 49 and 50 using cc-pVTZ (-f) (cc-pVTZ-pp (-f) on the iododerivative 26). The CEPs obtained did not significantly change nor alter our conclusions either. Graphs comparing basis sets (S2 File) and tabulated data are provided in the Supporting Information (S2 Table).

## Results and discussion

Fifty-nine fragments have been selected for this study based on their utility in drug design. Some have been reported previously in the literature. Although some fragments have already been reported, they were re-analyzed to allow direct comparison with structurally related fragments.

**Fig 1:**
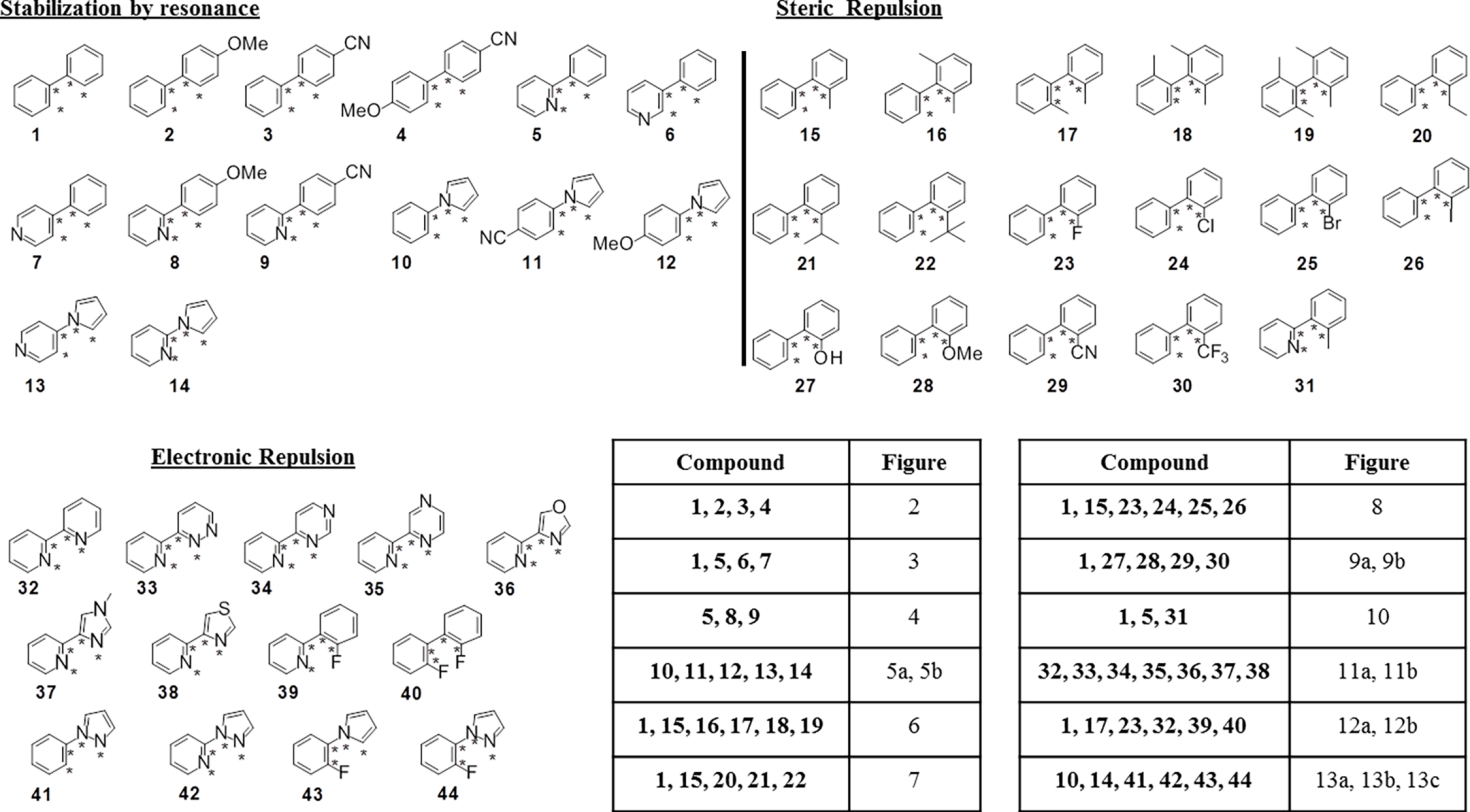
Aromatic biaryl systems distributed in three groups based on the major factor that leads to their corresponding CEP. The dihedral angle being studied in each of the fragments is indicated with stars, with the orientation as drawn defining 0°. Table indicates the figure where each CEP is represented in the paper.

The forty-four biaryl systems studied are displayed in Fig 1.

### Resonance stabilization studies

**Fig 2:**
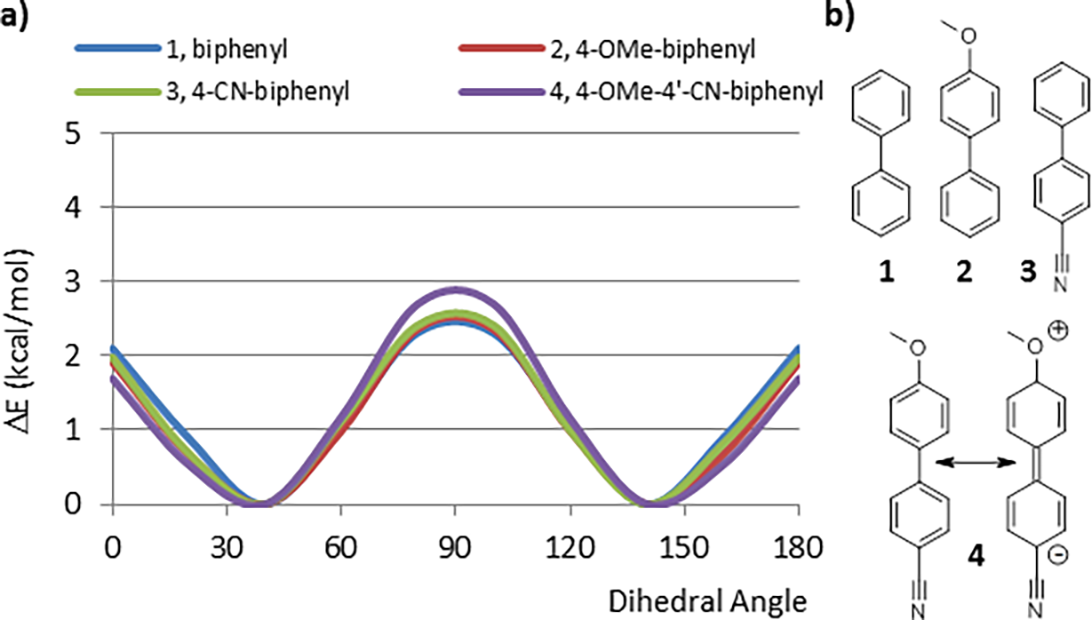
a) Comparison of the CEP for biphenyl 1 in with that of the *para* substituted biphenyls (2–4), b) Representation of fragments 1, 2 and 3 plus the charge-separated resonance form of structure 4.

The calculated CEP of biphenyl (1, Fig 2) displays an energy minimum at 40°, which agrees well with the experimentally determined [18] value of 44.4° and previously reported quantum mechanics results. [19] To investigate the significance of resonance stabilization, we studied how the addition of an electron donor and/or acceptor group at the *para* position of biphenyl affects the CEP. The CEPs of 2–4 are virtually identical to that of 1, indicating that the charge-separated resonance forms offer little, if any, additional stabilization to the planar conformation.

**Fig 3:**
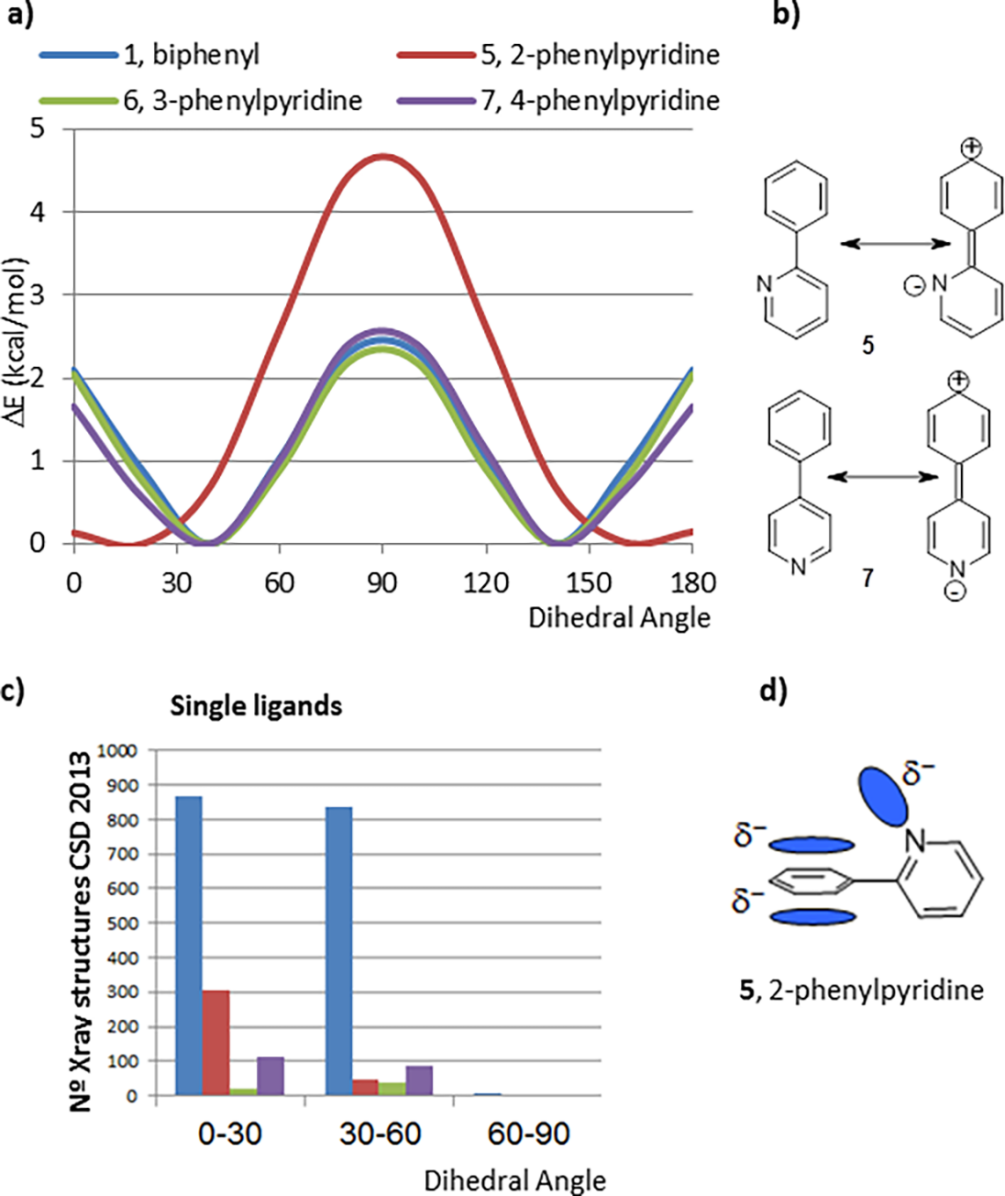
a) Comparison between biphenyl 1 and phenylpyridines (5, 6, 7), b) Charge-separated resonance forms for 2- and 4-phenylpyridines, 5 and 7, c) Histogram extracted from ConQuest repository for single ligands that contain the corresponding fragment, d) Diagram shows a potential electronic repulsion at 90° between electron lone pair of nitrogen of pyridine and the pi electron cloud of the phenyl ring in 2-phenylpyridine fragment, 5.

Resonance stabilization was further studied by calculating the CEPs of 2-, 3-, and 4-phenylpyridine (5–7) (Fig 3A). While the CEPs of 3- and 4-phenylpyridine (6, 7) closely resemble that of biphenyl, the CEP of 2-phenylpyridine (5) displays energy minima at 0° and 180° with the energy maximum conformation being the perpendicular conformation, over 4 kcal/mol higher in energy with respect to the minimum.

The charge-separated resonance forms of 5 and 7 (Fig 3B) should have similar resonance stabilization, so the different CEP of 5 compared to 1, 6 and 7 is likely due to a decrease in the steric interaction between the rings because of the elimination of one hydrogen-hydrogen interaction. As a result, the energy minima for 5 are the planar rotamers. Electronic repulsion between the electron lone pair of the nitrogen in the pyridine and the pi cloud of the phenyl ring [20] also likely contributes to the higher, meaningful (ΔE > 4kcal/mol) energy increase observed in the CEP of 5 at 90° (Fig 3D). This interaction is only present in this system because the orientation of the electron lone pair of the nitrogen for 3- and 4-phenylpyridines (6, 7) is not directed toward the pi electron cloud of their corresponding phenyl rings.

**Fig 4:**
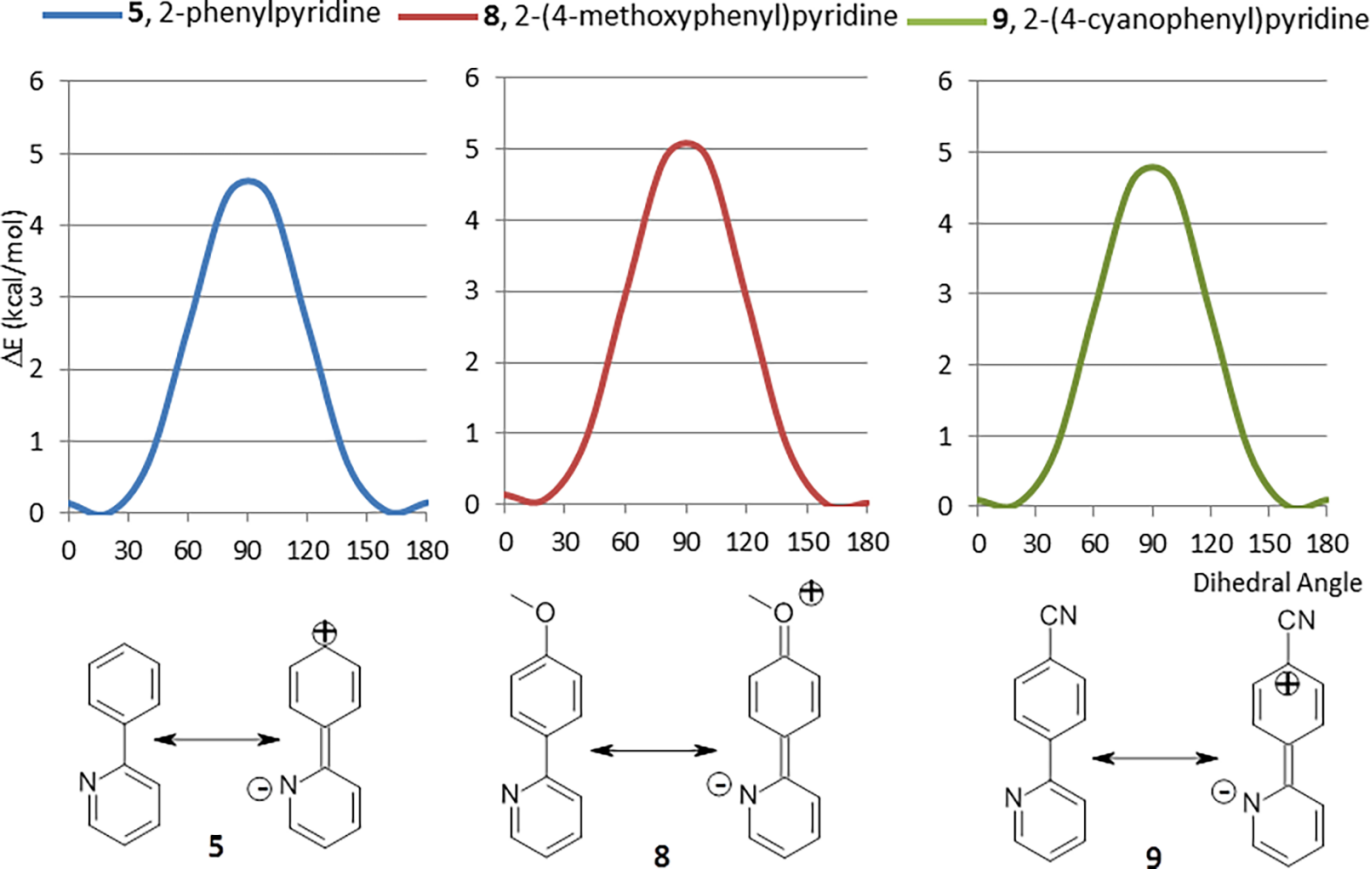
Comparison of the CEPs of 5, 8 and 9 with their corresponding charge-separated resonance forms.

The absence of crystal structures with torsional angles between 60–90° in the total of 349 structures that contain a 2-phenylpyridyl fragment in the single ligands ConQuest repository correlates with their CEPs (Fig 3C red, green, and purple bars). The relatively minor contribution of resonance stabilization to the CEP of 5 can be further deduced from the derivatives in which an electron donating (methoxy group) or electron withdrawing (cyano group) substituent has been added to the *para* position of the phenyl ring (8 and 9) (Fig 4), in neither case does the CEP change significantly from 5.

**Fig 5:**
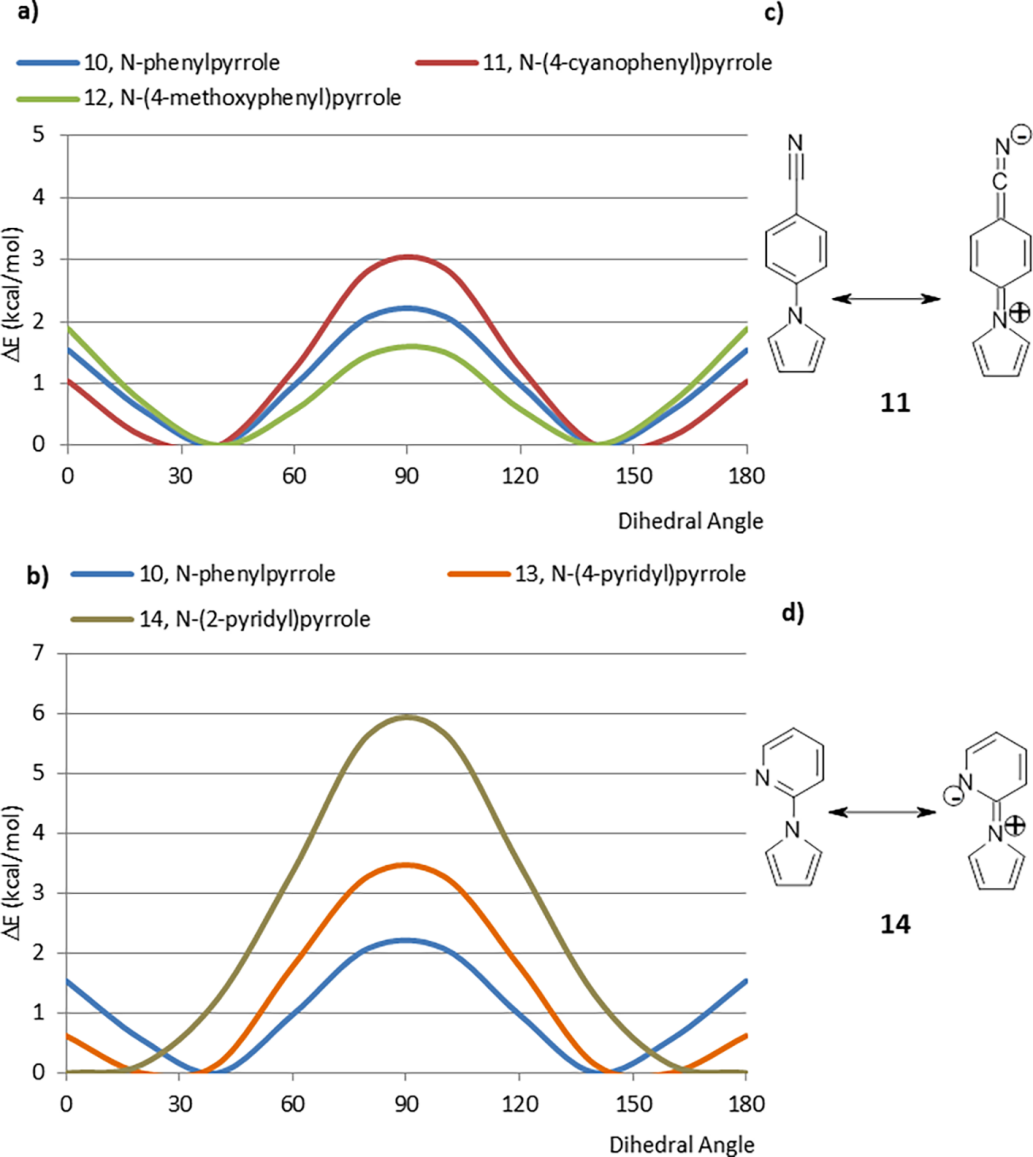
a) CEPs of several *N*-phenylpyrrole derivatives, b) Charge-separated resonance stabilization of 4-cyano-phenyl-pyrrole (11), c) CEPs of several *N-*pyridinepyrrole derivatives, d) Charge-separated resonance stabilization of 4-cyano-phenyl-pyrrole (14).

The situation is slightly different in the case of phenylpyrroles (Fig 5A) and pyridylpyrroles (Fig 5B). The CEP of the parent system, 10, bears a strong resemblance to that of biphenyl, 1. Introduction of a *para*-cyano group, 11, provides a CEP that demonstrates a modestly increased stabilization of the planar conformations, an effect that would be expected by stabilizing the charge-separated resonance structure (Fig 5C). The opposite effect is seen upon introduction of an electron donating (methoxy group) substituent (12). This is likely due to a decreased steric interaction between the pyrrole hydrogens and the *ortho* hydrogens of the pendant aryl ring than in their biphenyl counterparts which allows resonance effects to be more predominant in the pyrrole containing fragments.

The *N*-pyridylpyrroles show larger effects. The CEP of the 4-pyridyl analogue 13 is very similar to that of 11, consistent with similar degrees of stabilization of the charge separated resonance structures (Fig 5D). The effect is more dramatic with the 2-pyridyl analogue (14). In this case, the minimum energy conformation is planar and the maximum energy perpendicular, conformation is about 6 kcal/mol higher. The preference for the planar conformation can be explained as a consequence of stabilization of charge-separated resonance forms and the simultaneous elimination of one hydrogen-hydrogen interaction.

### Steric repulsion studies

**Fig 6:**
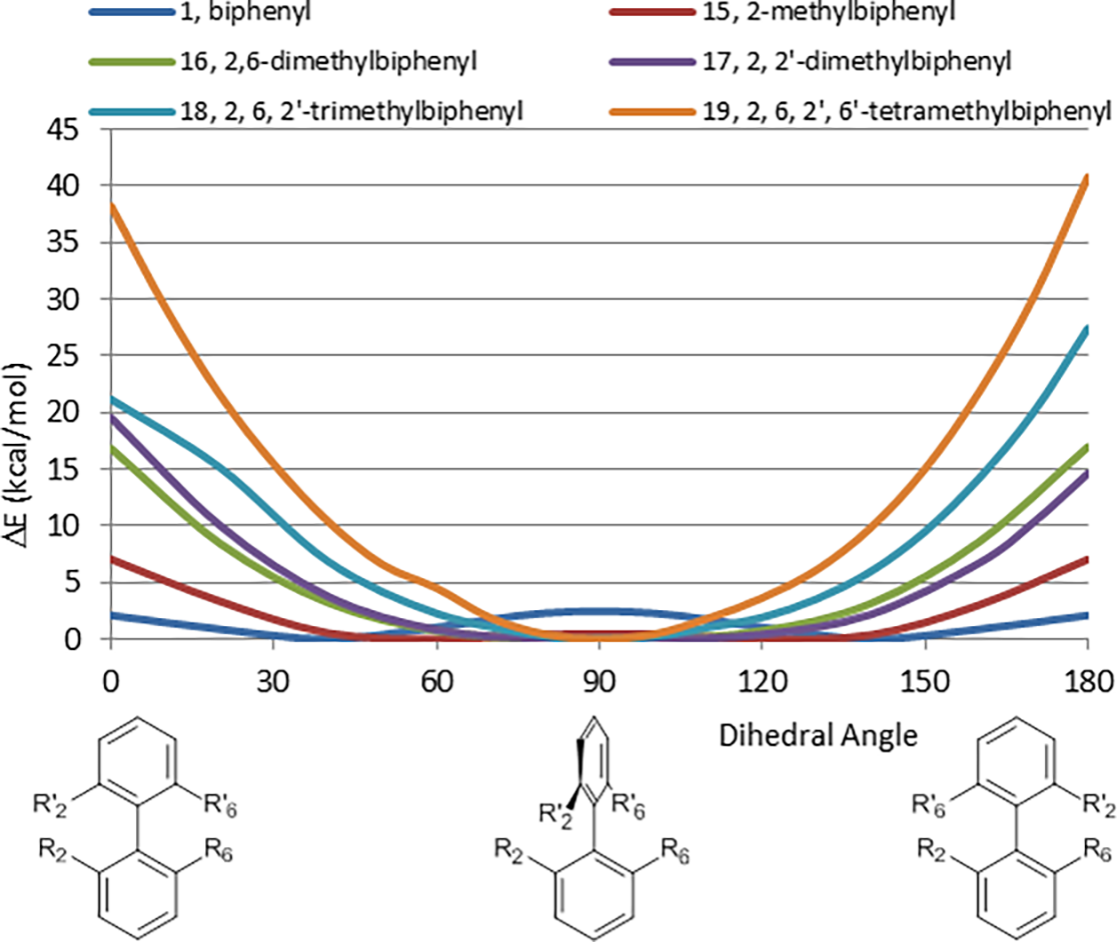
CEPs of series of *ortho*-methyl substituted biphenyls (15–19) and how they compare with unsubstituted biphenyl, 1.

We initiated our investigation of steric effects by studying a series of *ortho*-methyl substituted biphenyls (Fig 6). As expected, the CEP of 2-methylbiphenyl (15) displays a destabilization of the planar conformation relative to biphenyl, with the planar conformation falling outside the 4 kcal/mol threshold delineated by Hao, *et al* [8]. As substitution of the *ortho* positions increases, the energy of the planar conformations increases and the perpendicular conformations become more energetically favorable. The CEP of 2, 6-dimethylbiphenyl (16) is nearly identical to that of 2, 2’-dimethylbiphenyl (17).

For the tri- and tetramethyl analogues (18, 19), energetically accessible conformations are focused on a narrow range close to perpendicular. Structures obtained by optimization at 0° and 180° dihedral angles, where there is a strong steric interaction, are distorted. Although both these conformations should show the same energy values, they differed due to the distortions produced by the strong steric repulsion between methyl groups and the energy values at least for the higher energy observed for these supposedly identical conformations are not optimal. Exact values for these energies are, however, unimportant for our purpose, since they are clearly so high at any angle more than 50° from perpendicular, that their population will be minuscule.

As a general rule, the energy difference between the lowest energy planar and perpendicular conformations for *ortho*-methyl substituted biphenyls can be estimated by adding 7 kcal/mol for each hydrogen-methyl interaction and 20 kcal/mol for each methyl-methyl interaction.

**Fig 7:**
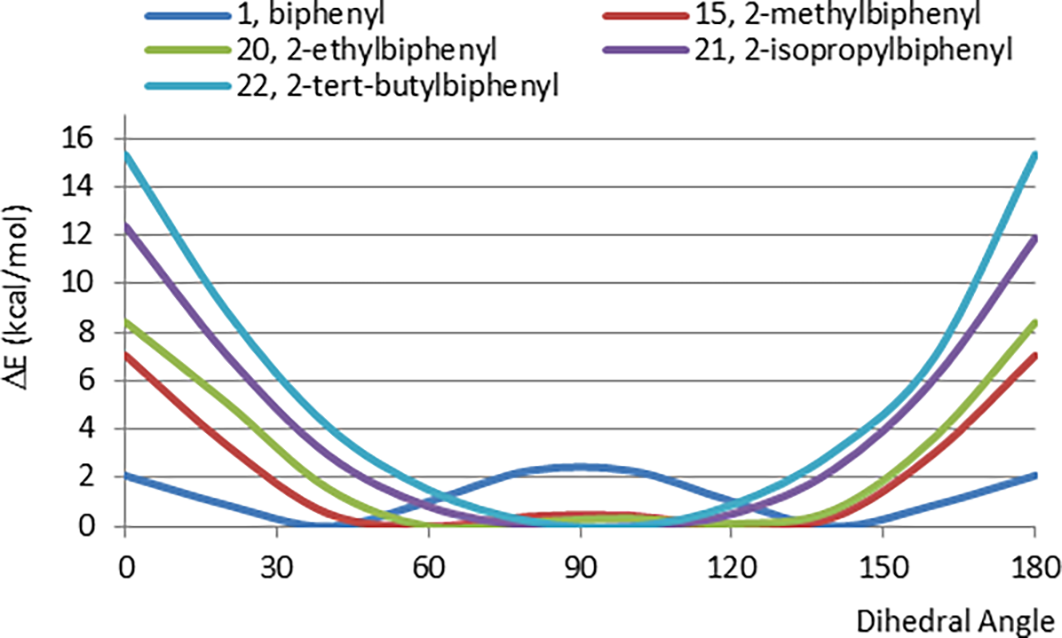
CEPs of 2-alkylbiphenyls (ethyl, *iso*-propyl and *tert*-butyl, 20–22) and the comparison with biphenyl (1) and its methyl analogue (15).

We next investigated the steric effects of larger alkyl groups in the 2-position of the biphenyl system (Fig 7). The CEP of 2-ethylbiphenyl (20) is nearly identical to that of the methyl analogue 15. Examination of the energy minimized planar conformation reveals that this is because the terminal carbon of the ethyl substituent is oriented away from the biphenyl ring system, so the effective steric interactions with the adjacent phenyl ring are very similar to those of a methyl group. For the *iso*-propyl and *tert*-butyl analogues (21 and 22), there is a corresponding increase in energy of the planar conformations. Hence, for 2-alkylbiphenyls, the trend for the energy difference between the planar and perpendicular conformations increases in the series: H < Me ~ Et < *i*-Pr < *t*-Bu. It should be noted that for 22, the effect of the *tert*-butyl group on conformation is less than what is observed for the dimethyl biphenyl derivatives 16 and 17.

**Fig 8:**
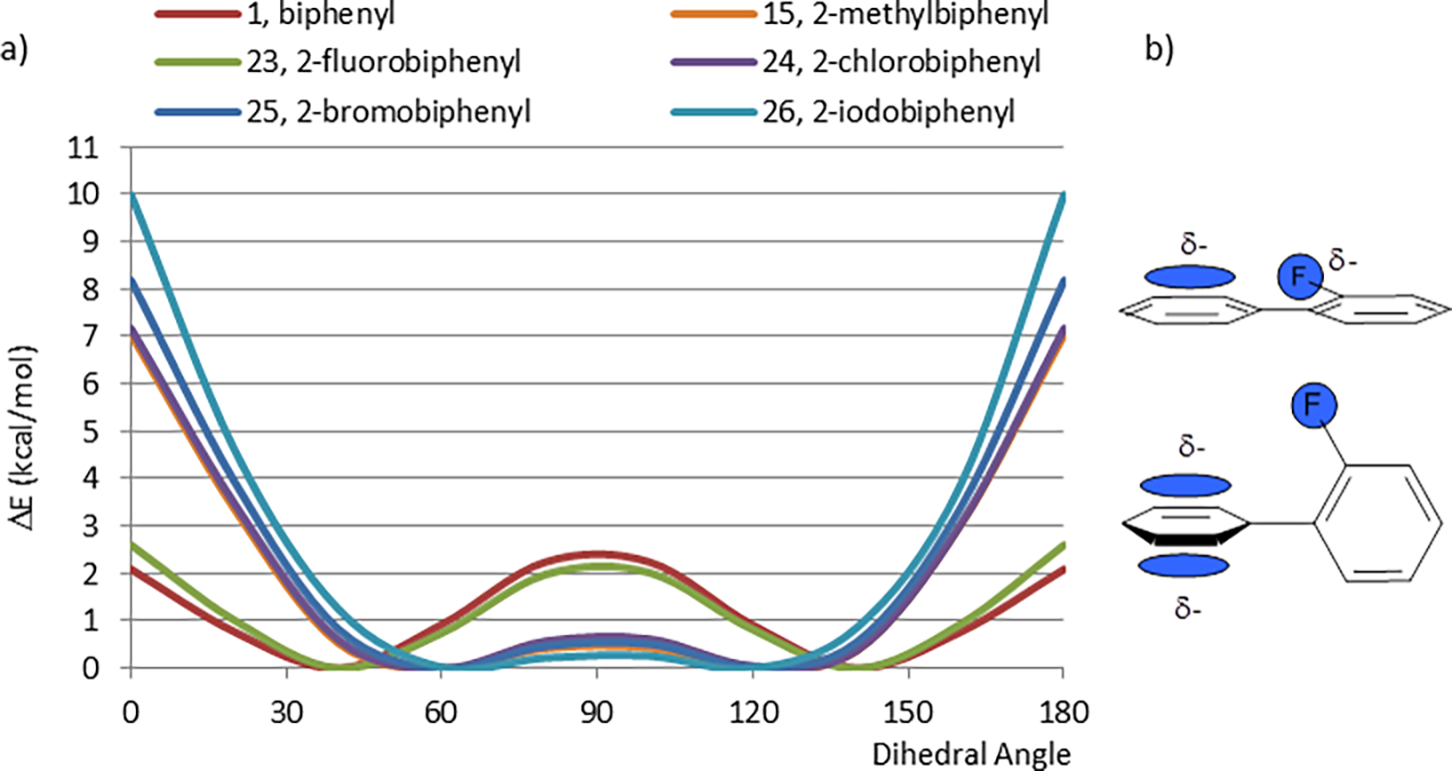
a) CEPs of a series of 2-halobiphenyls (23–26) and comparison with biphenyl 1 and 2-methylbiphenyl 15; b) Electrostatic fluorine—pi cloud aromatic ring attraction and repulsion.

Because exponents and coefficients for bromine and iodine are not available for DFT with the same standard 6-31G* basis set, energy calculations for the 2-halobiphenyls were performed using lacvp* basis set. The CEPs of biphenyl (1) and 2-methylbiphenyl (15) were also recalculated with the lacvp* basis set and no significant differences from the previous calculations were seen. The CEPs of 1, 15 and the 2-halobiphenyls (23–26) are displayed in Fig 8A. The CEP of 2-chlorobiphenyl (24) is nearly identical to that of 2-methylbiphenyl (15) and the energies of the planar conformations of 2-bromo- (25) and 2-iodo-biphenyl (26) increase further, as would be expected from the increasing size of these substituents. It is interesting to note the similarity between the CEPs of biphenyl (1) and 2-fluorobiphenyl (23), in spite of the larger size of the fluorine substituent with respect to the hydrogen (van der Waals radii: 1.47 Å for F atom vs 1.10 Å for H atom [21]). As seen in 2-phenylpyridine (5), this is partially explained by lone pair–pi cloud repulsion. In addition, it may be that the electronegative fluorine substituent has a favorable interaction within the positive electrostatic potential on the ring edge of the aromatic phenyl group which translates into an increase of the energy of the perpendicular conformation while stabilizing the planar conformation, thus balancing any steric effect (Fig 8B). A similar type of electrostatic attractive interaction has been reported in the literature as anion—pi interaction in a ligand-protein complex in X-ray crystal structures [22]. A closely related effect may also be playing a role in the CEP of 2-phenylpyridine (5).

**Fig 9:**
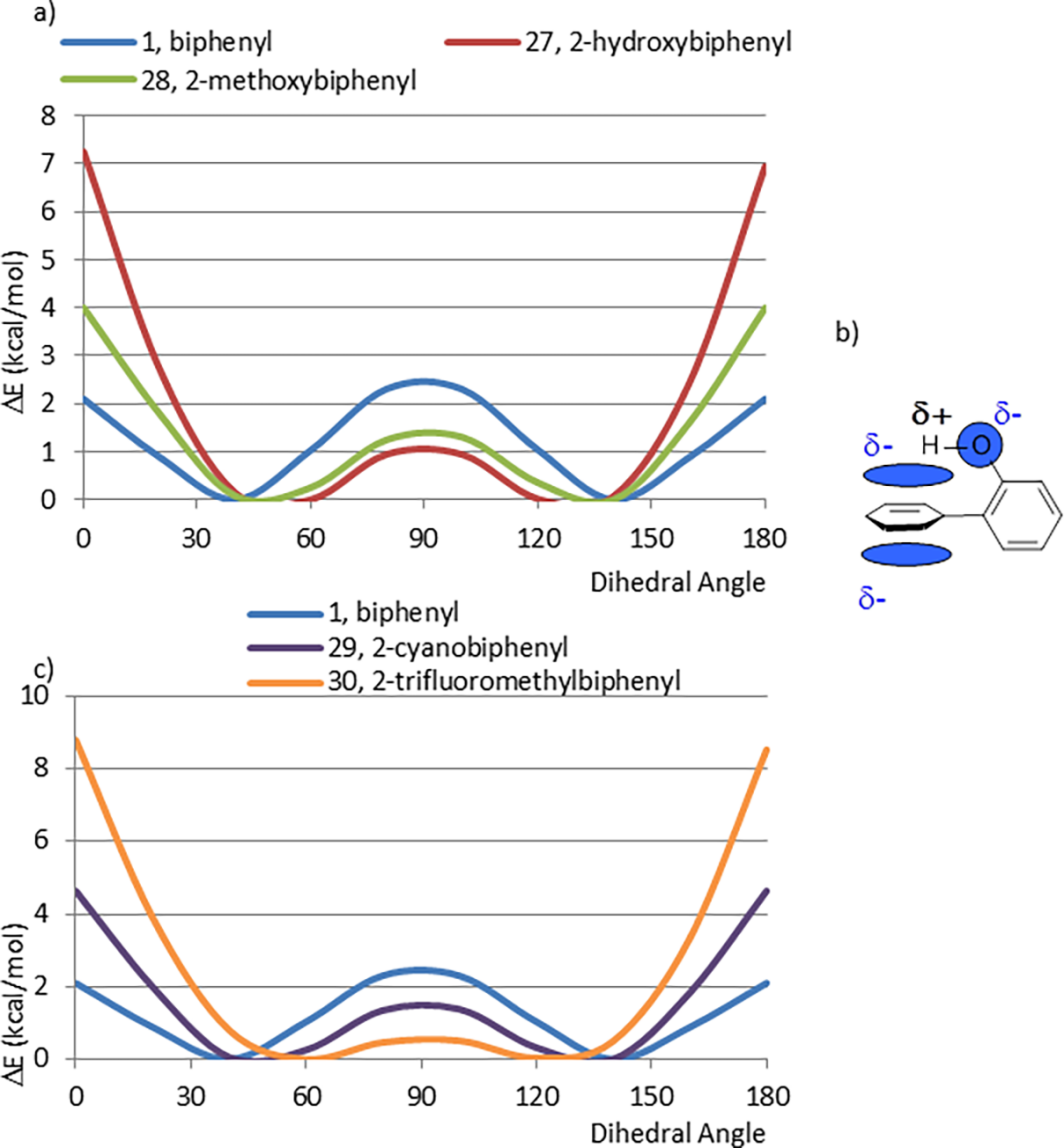
a) Comparison between CEPs of biphenyl and *ortho* substituted hydroxyl and methoxy derivatives, b) Diagram that represents the attractive interaction between the electrostatically positive hydrogen of the hydroxyl group and the negative pi cloud of the pendant phenyl ring, c) Comparison between CEPs of biphenyl and *ortho* substituted cyano and trifluoromethyl derivatives.

Corresponding effects may be responsible for the observed CEPs of 2-hydroxy- and 2-methoxybiphenyl (27, 28) (Fig 9A). The energy of the planar conformation of 2-methoxybiphenyl (28) is considerably lower than that of the “isosteric” 2-ethylbiphenyl (20), evidence for a similar destabilization of the perpendicular conformation to that seen in the 2-fluoro analogue (23). In contrast, the relative energy of the planar conformation of 2-hydroxybiphenyl (27) is considerably greater than that of the larger 2-methoxybiphenyl (28) in about 3kcal/mol. We propose that this is because rotation of the C-O bond of the phenol allows the fragment to present the electropositive hydrogen toward the electronegative pi cloud in the perpendicular conformation, resulting in an additional stabilizing interaction (Fig 9B). Reported high level *ab initio* calculations of the benzene-water (OH/ᴫ) intermolecular interaction quantified this interaction as much as 3.17 kcal/mol, well within the degree of stabilization inferred by our calculations of 27 [23]. This interaction has been also observed in dimer crystal structures [24]. Furthermore, it has been reported in the literature that O-H···pi hydrogen bonds exist, dominated by electrostatic interactions between the two systems [25]. Numerous examples are also found where peptide X—H…pi interactions are responsible for stabilization of helix termini, strand ends, strand edges, beta-bulges and regular turns [26].

Because cyano and trifluoromethyl groups are commonly used in medicinal chemistry, we calculated the CEPs of 2-cyanobiphenyl (29) and 2-trifluoromethylbiphenyl (30) (Fig 9C). The results agree well with what would be expected based on the size of these *ortho* substituents.

**Fig 10:**
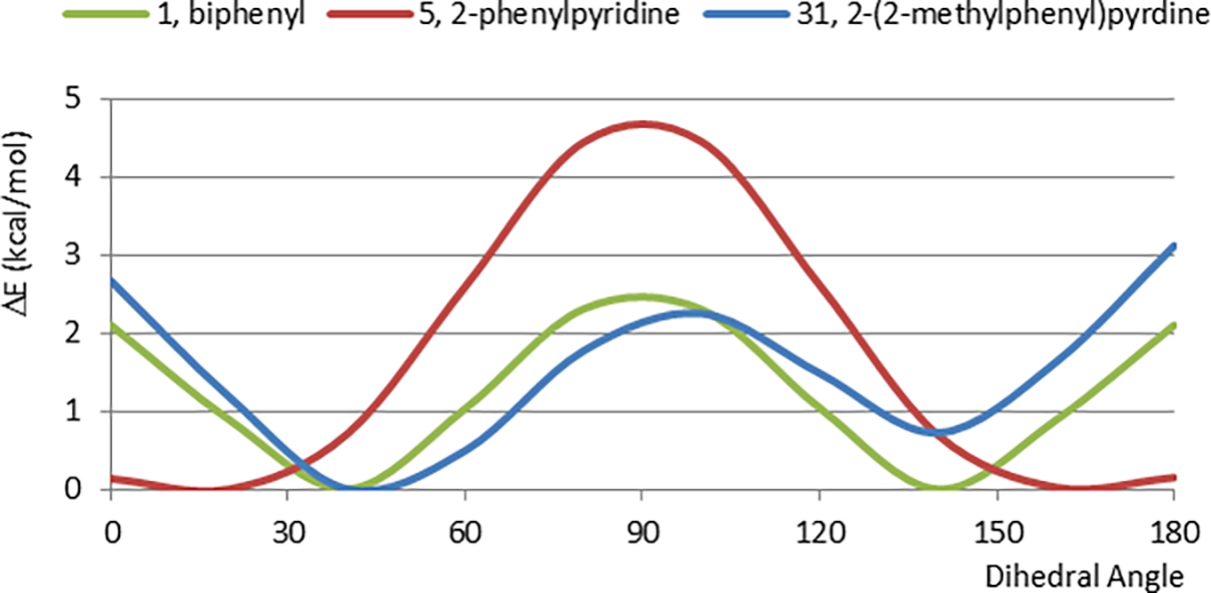
Comparison between biphenyl **1**, 2-phenylpyridine **5** and 2-(2-methylphenyl)-pyridine **31**.

A comparison of the CEP of 2-(2-methylphenyl)pyridine (31) (Fig 10) with that of the desmethyl analogue, 5, indicates the importance of steric factors in these systems. Introduction of the methyl group destabilizes both planar conformations to make them of comparable energy with one another and with that of the perpendicular conformation and the resulting CEP bears a close resemblance to that of 1. Because the entire CEP of 31 lies within an energy range < 4kcal/mol, this is a 2-phenylpyridyl substituted fragment in which the perpendicular conformation is energetically accessible.

### Electrostatic lone pair-lone pair repulsion

We initiated our studies of lone pair–lone pair electrostatic interactions by calculating the CEP of 32. The minimum energy conformation of 32 is the planar conformation in which the two nitrogen atoms are *anti* to one another, the planar conformation in which the nitrogen atoms are *syn* to one another and the perpendicular conformations are both 8–9 kcal/mol higher energy. These results replicate the findings of Chein and Corey who proposed that the *syn* conformation is strongly destabilized by a lone pair–lone pair repulsion [9]. In addition, we propose that the presence of hydrogen-hydrogen steric interactions between the two rings in the *syn* conformation and the lack of such interactions in the *anti* conformation, lead to further stabilization of the *anti* conformation. Rotamers with a dihedral angle about 90° in between both pyridine rings showed very high energy values. We believe this destabilization is due, in part, to repulsive interactions between the nitrogen lone pairs and the pi electron clouds.

**Fig 11:**
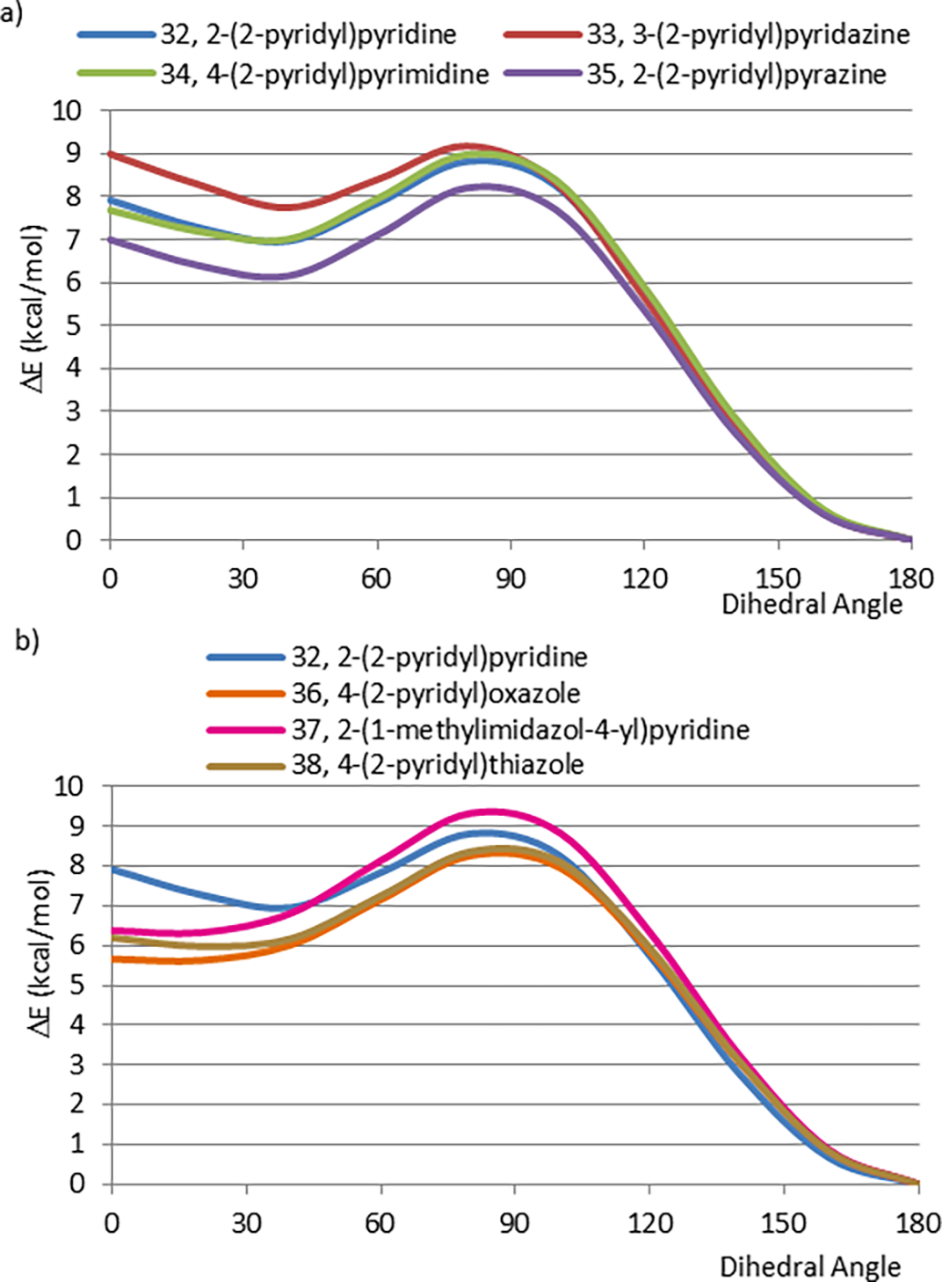
a) CEPs of (6, 6)-pyridyl biaryls, b) CEPs of (5, 6)-pyridyl biaryls.

The nitrogen lone pair of pyridine is responsible for its basicity and for the electronic interactions we observe in the CEP of 32. Since the availability of the nitrogen lone pair influences basicity we investigated whether replacing a basic pyridine ring (pKa = 5.14, water, at 20°) [27] with a correspondingly less basic pyridazine (pKa = 2.44), pyrimidine (pKa = 1.23) or pyrazine (pKa = 1.23) ring (33–35) [28] would change the CEP. In all cases (33–35), the CEPs are very similar to that of 32 (Fig 11A). Hence, the CEPs of these fragments are not influenced by the basicity of the interacting nitrogen atoms. (In response to a reviewer’s comment, we have calculated the CEP of 4-(4-pyrimidinyl)-pyrimidine, a fragment in which neither ring has appreciable basicity. The resulting CEP is nearly identical to that of 32 (the comparison can be found in the Supporting Information (S1 Fig)) further demonstrating that the CEPs are not influenced by the basicity of the system.).

We extended these studies to include the 5-membered ring heterocyclic analogues 36–38 (Fig 11B). The CEP of the oxazole (36, pKa = 1.67 [29]), *N*-methylimidazole (37, pKa = 7.1) [30] and thiazole (38, pKa = 2.68) [29] analogues are very similar to that of 2, 2’-bipyridyl (32), with the exception being that the *syn* planar conformation is approximately 2 kcal/mol lower in relative energy. We believe that this energy difference is due to the fact that the smaller intra-annular angle of these five membered ring analogues causes the nitrogen lone pair to project further from the *syn* nitrogen in the pyridine ring. This electronic effect is related to the steric effects seen in aryl-pyrroles (10–14). In summary, there is no correlation between the basicity of the heterocycles and their CEPs in the pyridine analogues (32–38).

**Fig 12:**
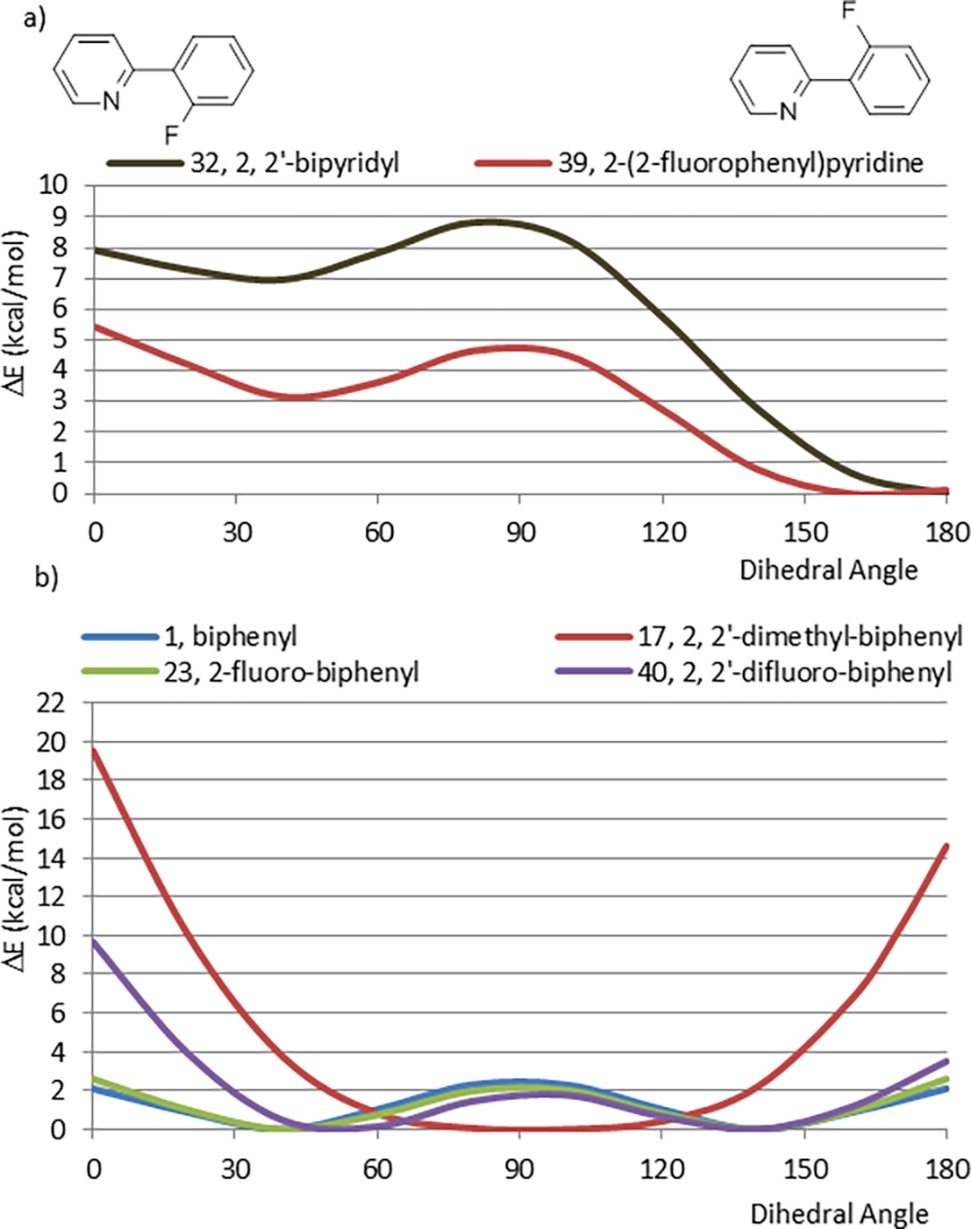
a) Comparison between 2, 2’-bipyridyl (32) and 2-(2-fluorophenyl)pyridine (39), b) Comparison of CEPs between biphenyl systems with and without methyl or fluorine atoms in ortho positions (1, 17, 23, 40).

The CEP of 2-(2-fluorophenyl)pyridine (39) (Fig 12A) bears a strong similarity to that of 2-(2-pyridyl)pyridine (32) where the conformational preference is driven by electrostatic repulsions, however, the energy of the perpendicular and planar *syn* rotamers are about 2 kcal/mol lower energy for 39 than 32. This indicates that, in planar *syn* conformations, the energy of repulsion between a fluorine atom and a nitrogen lone pair is less than that observed between two nitrogen lone pairs. Similarly, in the perpendicular conformation, the aromatic pi electron cloud exhibits a stronger repulsion with a nitrogen lone pair than with a fluorine atom. However, in spite of this smaller energy difference, for fragments, 32 and 39, the energetically accessible conformations (those within 4 kcal/mol of the global minimum) lie within 50° of the planar *anti* conformation.

Compared with biphenyl (1) and 2-fluorobiphenyl (23), the CEP of 2, 2’-difluorobiphenyl (40) shows a severe eclipsed interaction for the planar *syn* rotamer (dihedral < 30°) where the two fluorine atoms interact (Fig 12B). This is most likely due to electrostatic factors rather than steric ones, since, in 2, 2’-dimethylbiphenyl (17), the planar *anti* rotamer is of comparable energy to the *syn* planar conformation. In contrast to 32 and 39, fragment 40 exhibits a much larger range of energetically accessible conformations, with conformations within 150° of the planar *anti* conformation all being within 4 kcal/mol of the global energy minimum.

**Fig 13:**
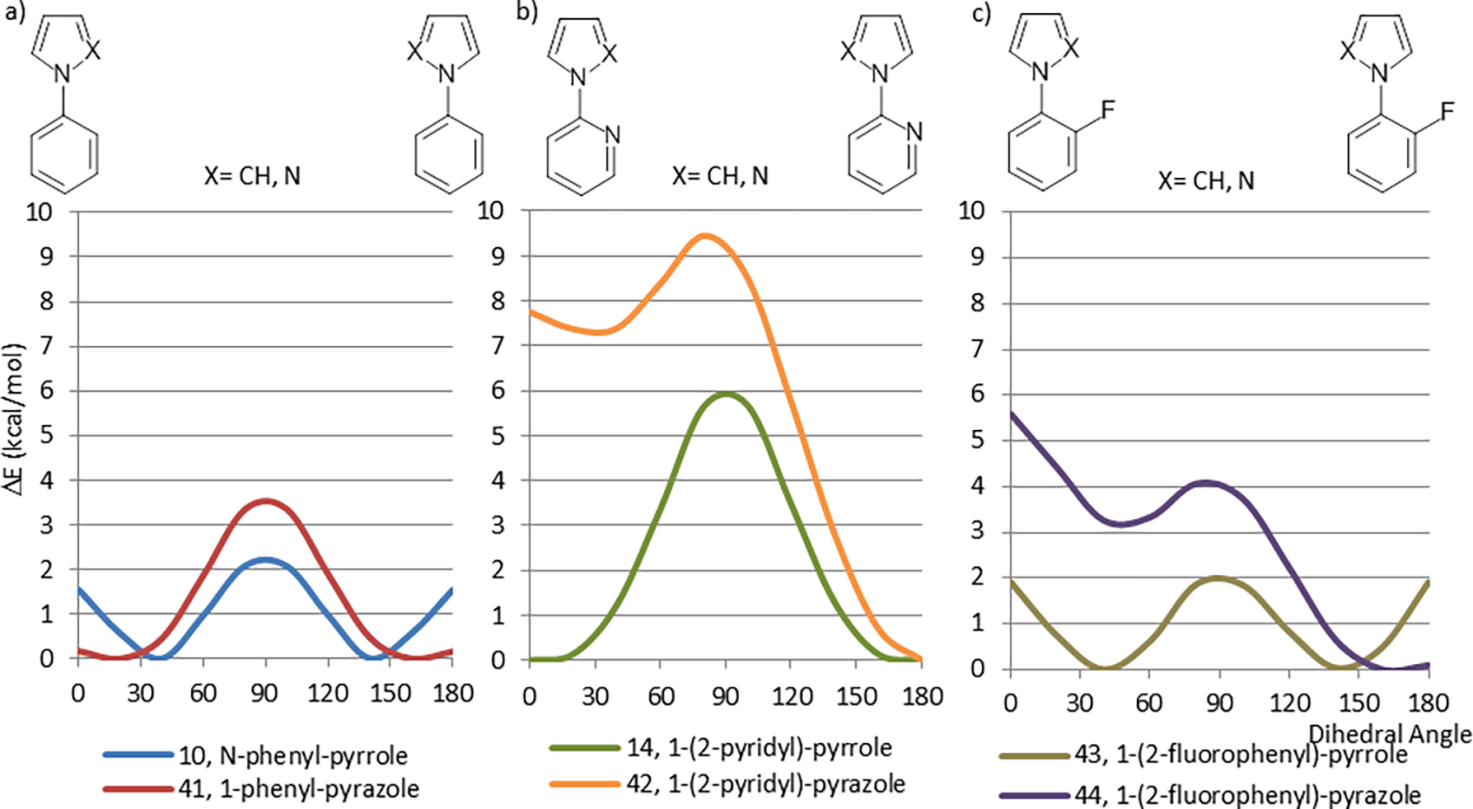
a) Comparison between CEPs of structures containing a phenyl ring, 10 and 41, b) Comparison between CEPs of structures that contain a pyridine ring, 14 and 42, c) Comparison between CEPs of structures that contain a 2-fluoro-phenyl ring, 43 and 44.

We have made analogous observations for compounds related to *N*-phenylpyrroles 10–12. For 1-phenylpyrazole (41, Fig 13A), the planar rotamer is the lowest energy conformation. This is likely a reflection of the loss of one transannular hydrogen-hydrogen interaction, analogous to what was observed with 2-phenylpyridine, (5) and 1-(2-pyridyl)pyrrole, (14). As expected, the low energy conformer of 1-(2-pyridyl)pyrazole (42) is the planar *anti* rotamer with the perpendicular and planar *syn* conformations being of considerably higher energy (Fig 13B). These lone pair repulsion effects are similar to what was seen with the pyridyl-heteroaryl fragments (32–38). The fluorine-substituted pyrrole analogue 43 (Fig 13C) has a CEP that is very similar to the non-fluorinated parent 10. Any steric repulsion of the fluorine is likely cancelled by a positive interaction with the electropositive region within the plane of the pyrrole ring, the same behavior that is seen in fluorinated (6, 6)-biaryls. The introduction of the second nitrogen atom in fragment 44 (Fig 13C) adds an electrostatic repulsion component which increases the energy of the perpendicular and planar *syn* conformations. It should be noted that this effect is less than what was observed for 42, consistent with the other calculations showing that a fluorine atom has a smaller effect on the CEP than a nitrogen lone pair.

### Extension to aromatic carbonyl systems

**Fig 14:**
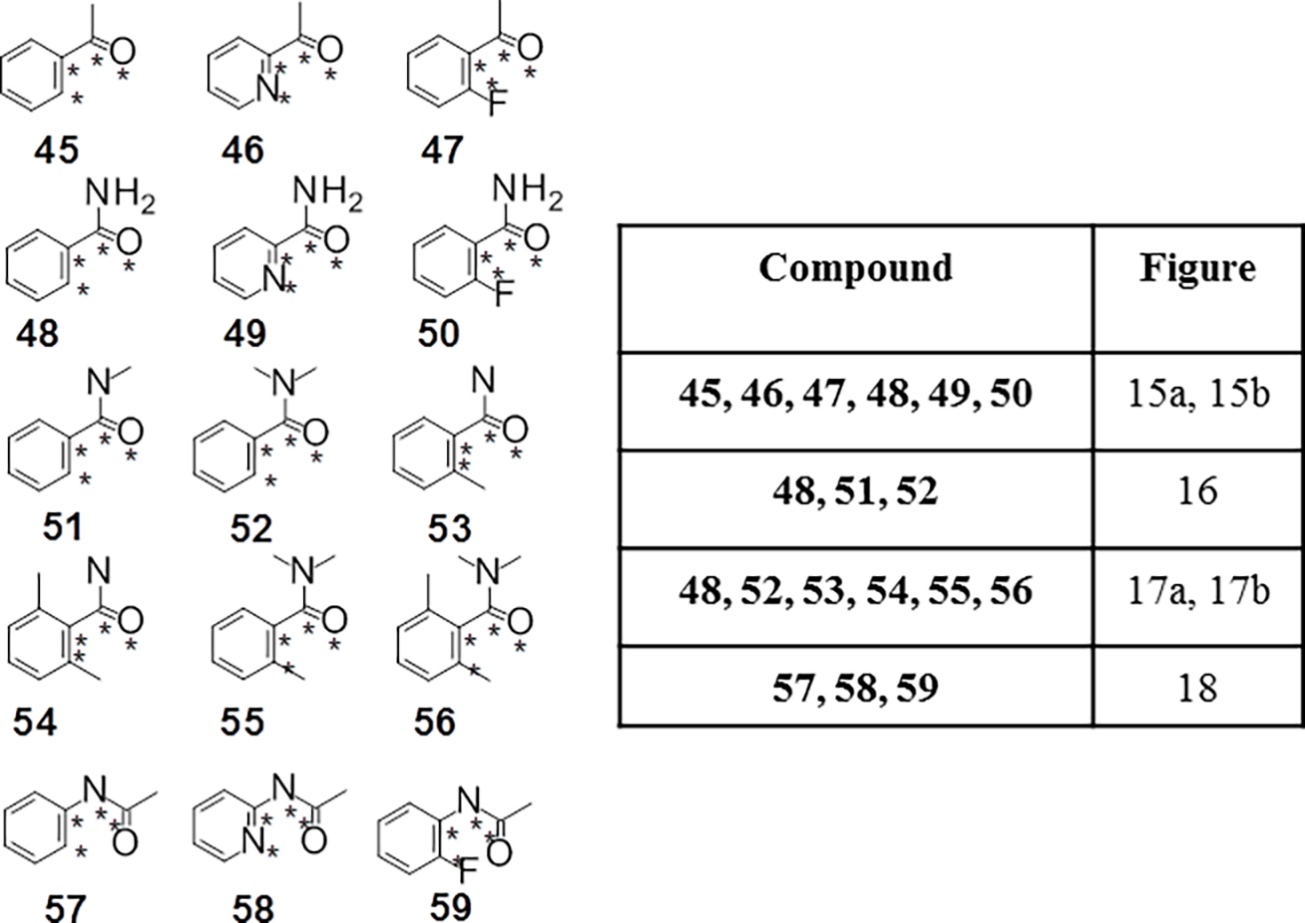
Aromatic carbonyl systems and table that indicates which figure each CEP is found in. The dihedral angle being studied in each of the fragments is indicated with stars, with the orientation as drawn defining 0°.

Fifteen fragments (Fig 14) were studied to evaluate how resonance, steric and lone pair repulsion events determined the CEPs of aryl carbonyl fragments.

**Fig 15:**
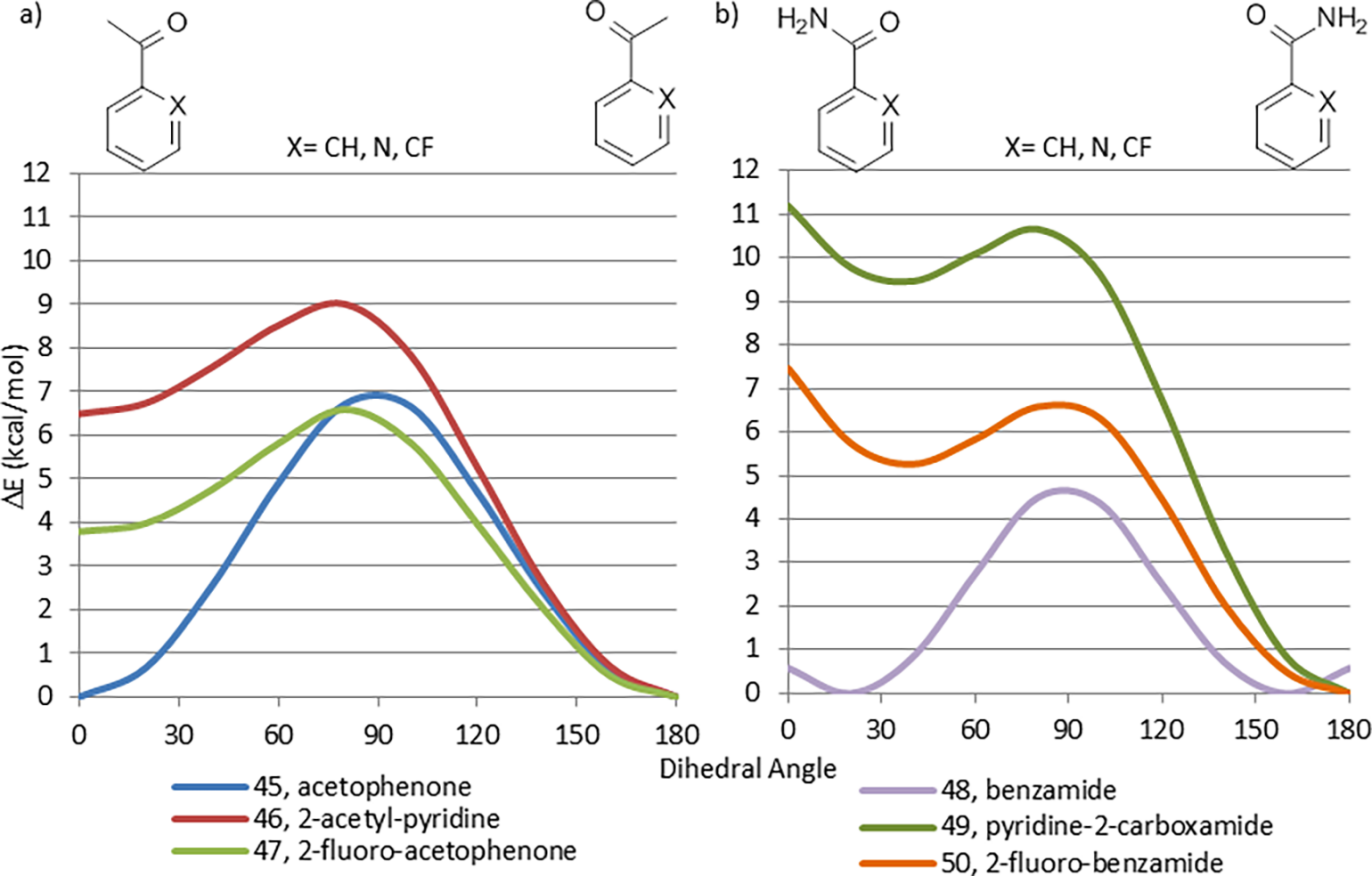
a) Comparison between CEPs of aryl acetyls, b) Comparison between CEPs of aryl primary amides.

The CEPs of acetophenone (45), 2-acetylpyridine (46) and 2-fluoroacetophenone (47) are displayed in Fig 15A. Planar conformations are strongly favored for all three systems and the perpendicular rotamer is the highest energy conformation. As expected, lone pair repulsion effects destabilize the planar *syn* conformations in 46 and 47, with the nitrogen of 46 having a bigger effect than the fluorine in 47. It is surprising that a nitrogen lone pair should exert a stronger effect than an *ortho*-fluorine atom, which has three lone pairs and is located closer to the interacting carbonyl oxygen. Accordingly we calculated CEPs of 42, 44, 46, 47, 49 and 50 using the more complex cc-pVTZ(-f) basis set (S2 File). These CEPs are very similar to the ones performed using 6-31G*, confirming our findings. We cannot offer a qualitative explanation for the greater effect of the ring nitrogen than an ortho-fluorine atom.

The CEPs of the corresponding primary amide fragments 48–50 (Fig 15B) are of similar shape, but the planar *syn* conformations are 3–5 kcal/mol higher energy than their corresponding planar *anti* conformations. As noted by Kuhn *et al*.[31], it is likely that this is due to a stabilizing intramolecular hydrogen bond between the amide N-H and the ring nitrogen and fluorine in 46 and 47, respectively, in the *anti* conformations. It should be noted that the global minimum energy of 48 occurs at 20°. This is likely due to adverse steric interactions between the amine N-H and the phenyl ring in the planar conformation.

**Fig 16:**
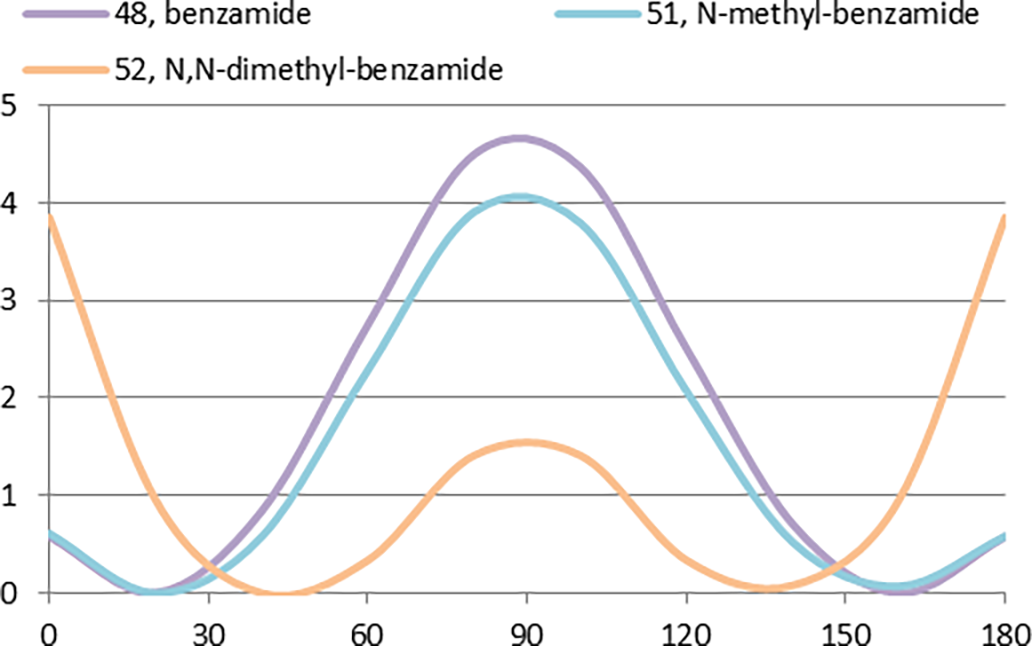
Effect of methylated amides in the CEP profile of benzamides 48, 51 and 52.

The CEPs of primary (48), secondary (51) and tertiary (52) amides are compared in Fig 16. Because of the thermodynamic preference of secondary amides to adopt the S*-trans* conformation, the profiles of benzamide (48) and *N-*methylbenzamide (51) are nearly identical, with the energy minima at 40° and 140° and the planar conformations being < 1 kcal/mol higher energy. For the *N*,*N*-dimethyl amide derivative (52), steric effects predominate and conformations within 20° of planar are energetically unlikely. It is interesting to note that a significant loss in a compound’s potency upon methylation of a secondary benzamide could be due either to the loss of a hydrogen bond donor-acceptor interaction with the target or because of a large change in ligand conformation.

**Fig 17:**
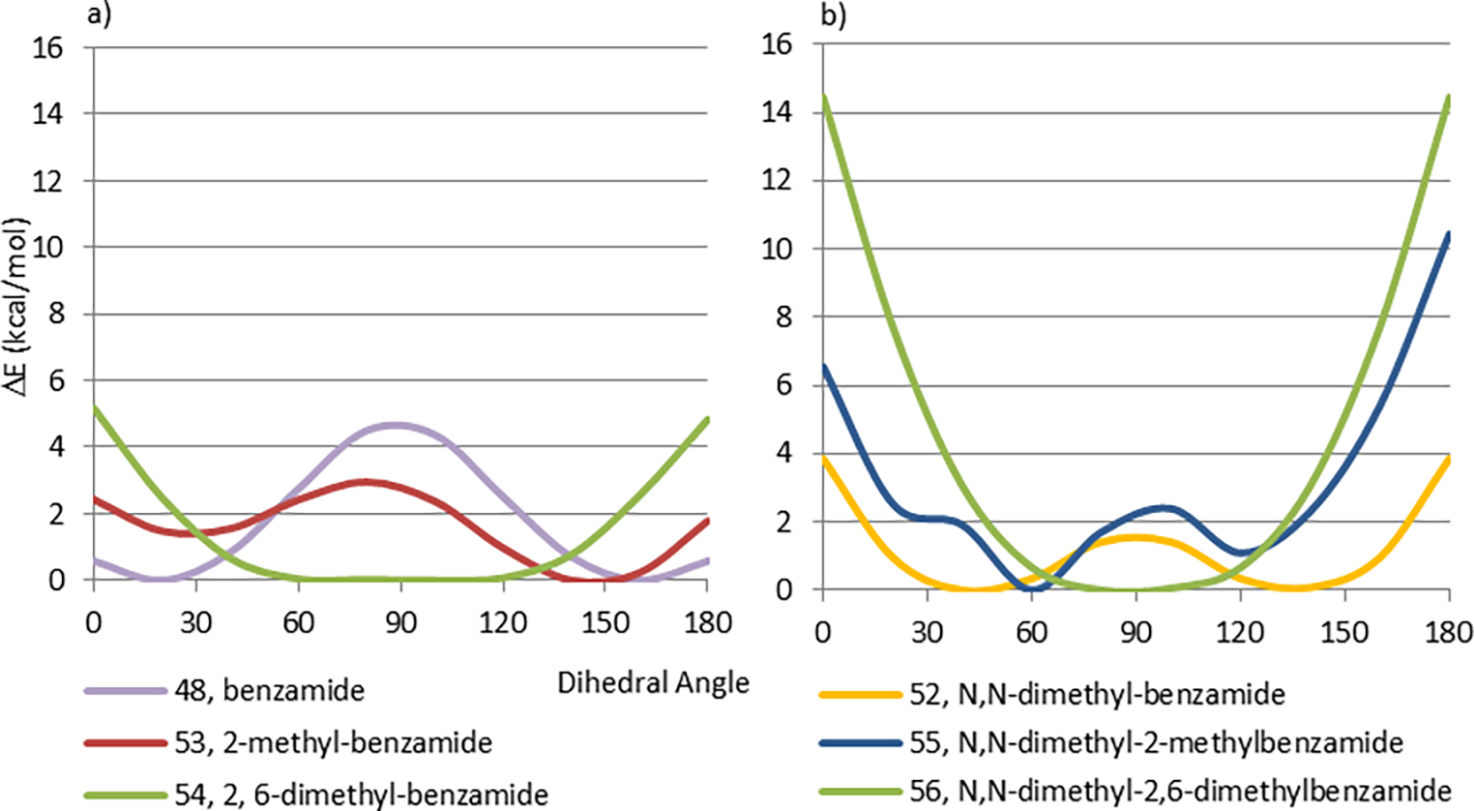
a) Effect of methyl groups in benzamide 48, b) Effect of methyl groups in dimethylated amides 52, 55 and 56.

Methyl substitution at the ortho position of these aryl-carbonyl systems has the same effect on the CEP as seen in biaryls, with increasing methylation favoring the perpendicular conformations over the planar ones. A comparison between benzamide (48), 2-methyl-benzamide (53) and 2, 6-dimethyl-benzamide (54) is displayed in Fig 17A. An even more pronounced effect is seen with the tertiary amides 55 and 56 (Fig 17B).

When exploring the SAR of a series of amides, one commonly used strategy is to reverse the orientation of the amide linkage [32]. Hence we were interested in also including N-aryl-acetamide fragments in our study.

**Fig 18:**
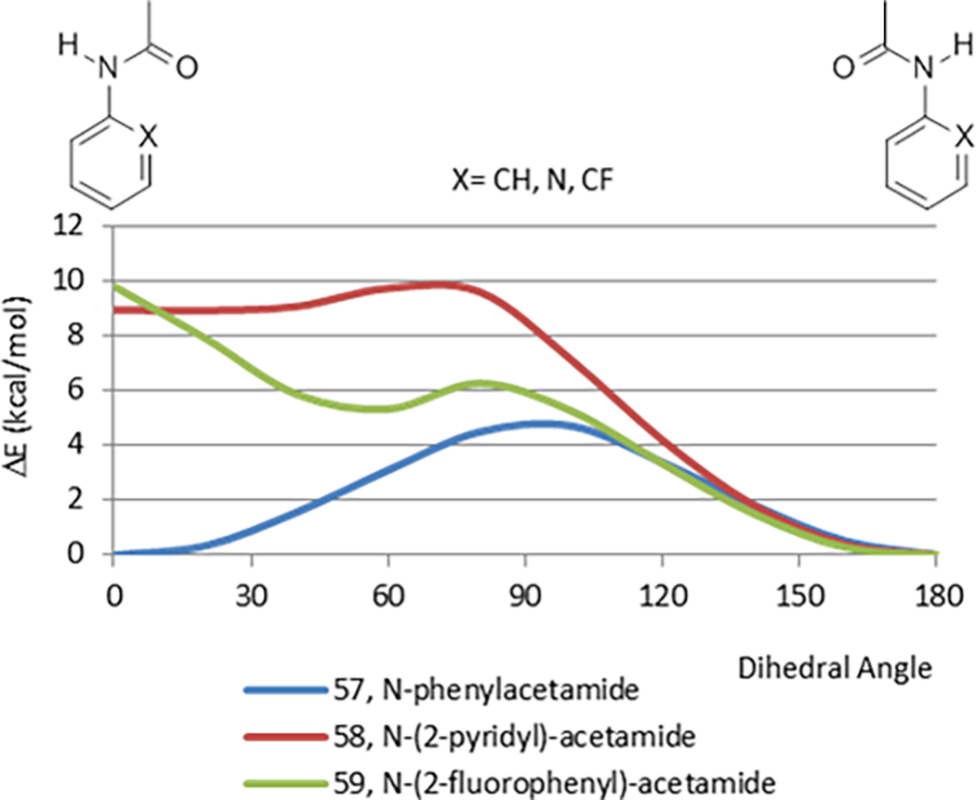
Comparison between CEPs of N-aryl-acetamides.

The CEPs of *N*-aryl-acetamides, 57–59 displayed in Fig 18 have very similar CEPs to the arylcarboxamide analogues 48–50 (Fig 15B). This similarity in CEPs may help explain why this “reverse amide” strategy is often successful. The planar conformation of *N*-phenylacetamide (57) is likely favored by resonance effects, the lack of strong steric interactions and repulsion of the carbonyl oxygen with the pi electron cloud of the benzene ring. Analogous to other aryl carbonyl fragments, the planar *syn* conformations of *N*-(2-pyridyl)-acetamide (58) and *N*-(2-fluorophenyl)-acetamide (59) are strongly destabilized by lone pair–lone pair interactions. In contrast to what was observed for the pyridine/fluorophenyl pairs 32/39, 42/44 and 49/50, the energy of the planar *syn* conformation of the fluorinated analogue 59 is comparable to that of the pyridine analogue 58. This is likely due to increased steric interaction of the fluorine in 59 over that seen in the other systems.

### Experimental validation studies

As a mode of experimental validation of our CEP calculations, we have performed searches of the CSD, ConQuest and Relibase databases, for the substructures 1–59 and extracted the value of the relevant dihedral angle of the structures found and determined whether dihedral angles are in regions of the calculated CEP that are >4 kcal/mol. This analysis is reported in detail in the Supporting Information (S1 File). From the Conquest data, we show that we have Positive Predictive Value of 0.91 and Specificity of 0.95. In other words, 91% of the time in which the CEP shows that a given conformation is >4 kcal/mol over ground state, so it shouldn’t exist, we do not find that conformation in the ConQuest database. If the rotamer does exist in the ConQuest database, 95% of the time, the CEP shows the energy of that rotamer to be <4 kcal/mol predicting its existence. The corresponding values for structures found in the Relibase database are Positive Predictive Value = 0.93 and Specificity = 0.98. This analysis demonstrates the predictive value of the CEP calculations.

## Conclusions

The conformational preferences of biaryl and aryl carbonyl systems can be interpreted as an interplay between resonance stabilization, steric effects and electrostatic interactions, which include lone pair-lone pair and pi cloud-lone pair interactions. The ground state of the parent biphenyl system (1) is non planar, however energy differences between the lowest and highest energy rotamers are small and no significant conformational bias is displayed. The parent aryl carbonyl system (acetophenone, 45) displays an energetic preference toward planar conformations. Structural modifications of these parent systems can lead to compounds with dramatically different CEPs. The energetic differences can be readily compared and quantified from the CEPs we have presented in Figs 2–13 and Figs 15–18. From our studies, we propose several “rules of thumb” regarding the relative importance of resonance stabilization, steric effects and electrostatic interactions on conformation of these systems;

- Resonance stabilization effects favor planar conformations in biaryl and aryl carbonyl fragments. However, they are rather weak and only slightly enhanced, if at all, by placement of electron-donating or electron-withdrawing substituents on the aromatic rings. These effects are slightly larger when a heteroatom in the ring links to the adjoining ring (Fig 5A). Resonance stabilization has the smallest influence on CEP and is so weak, that even *ortho*-hydrogen atoms exert a steric effect strong enough to override the resonance stabilization of planar conformers. The only structures we observed in which the planar conformation is the lowest energy conformation are those in which no more than one *ortho*-hydrogen–*ortho*-hydrogen interaction exists.
- Substituents in the *ortho* positions of the aromatic rings can have profound steric effects which favor non-planar conformations and are the most pronounced of the interactions we studied (up to 40 kcal/mol for tetrasubstituted biphenyls). The likelihood that the perpendicular conformation is favored increases both with the number of *ortho* substituents and the size of these substituents, with the number of *ortho* substituents being the more significant factor. As mentioned above, even *ortho* hydrogens exert a significant steric effect, as seen in the CEP of biphenyl and the finding that those fragments in which the minimum energy conformation is planar are ones in which at least one hydrogen-hydrogen steric interaction is missing (e.g. 2-phenylpyridine, 5). The preferred planar conformation of 5 can be overridden sterically by substitution with an *ortho*-methyl group (e.g. 31).
- *Ortho* steric interactions can be modulated by electrostatic lone pair–pi cloud interactions. *Ortho* substituents that possess an electron lone pair (e.g. fluorine, oxygen) may have a smaller than expected effect on conformation due to repulsive interactions between the lone pair and the pi electron cloud which destabilize the perpendicular conformation and attractive interactions between the lone pair and the in-plane electropositive region that stabilizes the planar conformation. Because of this effect, replacement of an *ortho*-hydrogen with a fluorine atom does not result in a significant change in the CEP. An *ortho*-hydroxy substituent more strongly favors the perpendicular conformation than an *ortho*-methoxy substituent because C-O bond rotation allows for an attractive hydroxyl–pi cloud interaction in the perpendicular conformation and, as a result, paradoxically exerts a larger *apparent* steric effect than the methoxy substituent.
- In biaryl systems in which a nitrogen is present in the *ortho* position of both aromatic rings (e.g. 2, 2’-bipyridyl) and in aryl carbonyl systems possessing an *ortho* nitrogen (e.g. 2-acetylpyridine), electrostatic repulsion of adjacent lone pairs strongly disfavors those conformations in which the heteroatoms are in proximity.
- Similar effects are observed in biaryl systems in which an *ortho* fluorine is present in one ring and a nitrogen in the *ortho* position of the other ring (e.g. 2-(2-fluorophenyl)-pyridine) and in *ortho* fluorinated aryl carbonyl systems. However, in these fragments, the electrostatic repulsive effect is less pronounced than seen for the nitrogen cases.

In general, the relative importance of these factors in determining the CEP of these systems is:

steric repulsion > lone pair—lone pair repulsion> lone pair–fluorine repulsion > resonance stabilization

Since these trends have been identified through calculations on the parent fragments, in complex molecules, these trends may be overridden by steric clashes or other long-range effects present in more highly-elaborated molecules.

PPV and Specificity values higher than 90% obtained from the study of a given rotamer of these fragments as part of complete molecules in ConQuest and Relibase CSD databases increases the confidence for the application of CEP for all fragments, including those for which no crystal structures have been reported.

The application of CEP calculations and the simple described rules of thumb can be used to quickly assess molecular conformations and understand SAR trends without the necessity of bespoke computations of the specific series; to ensure that fragment libraries sample an appropriate expanse of conformational space [33] and, prospectively, to design molecules with the desired conformational constraints.

## Supporting information

S1 Fig CEP of a non basic 4-pyrimidine-4-yl-pyridine vs 2-(2-pyridine)-pyridine, 32. (DOCX)

S1 Table DFT energy value in each studied dihedral angle using 6-31g* or lacvp*for each fragment. Tabulated data to build up the Conformational Energy Profiles for biaryl (**1–44**) and aryl carbonyl fragments (**45–59**) and the non basic 4-pyrimidine-4-yl-pyridine.
(DOCX)

S2 Table DFT energy value in each studied dihedral angle using cc-pvtz(-f) for biaryl (1, 4, 5–7, 15, 23–25, 31, 32, 42, 44) and aryl carbonyl fragments (45–47, 50) and cc-pvtz-pp(-f) for 26. (DOCX)

S1 File Experimental validation and statistical analysis of the method. Description of how torsional angle of the fragments were extracted from available crystal structures (CSD: ConQuest and Relibase) and tabulated data of frequency of appearance of fragment in a molecule in a defined torsional angle interval with statistical treatment of the data.
(DOCX)

S2 File Comparison between CEPs using simple and complex basis sets. Set of CEPs comparing CEPs obtained with 6-31g* or lacvp and cc-pVTZ (-f) (cc-pVTZ-pp (-f) for **26**, iodo derivative) basis sets for **1, 5–7, 23–26, 42, 44–47, 49, 50** fragments.
(DOCX)
