# Supplementary material for: Rapid assessment of conformational preferences in biaryl and aryl carbonyl fragments
Source: PLoS One. 2018 Mar 14;13(3):e0192974. doi: 10.1371/journal.pone.0192974 (PMC5851544; doi:10.1371/journal.pone.0192974)
Supplement: S1 Fig — (DOCX) [file pone.0192974.s001.docx]

**S1 Fig**. **CEP of a non basic 4-pyrimidine-4-yl-pyridine vs 2-(2-pyridine)-pyridine, 32**.
