# Supplementary material for: Rapid assessment of conformational preferences in biaryl and aryl carbonyl fragments
Source: PLoS One. 2018 Mar 14;13(3):e0192974. doi: 10.1371/journal.pone.0192974 (PMC5851544; doi:10.1371/journal.pone.0192974)
Supplement: S1 Table — Tabulated data to build up the Conformational Energy Profiles for biaryl (1–44) and aryl carbonyl fragments (45–59) and the non basic 4-pyrimidine-4-yl-pyridine. (DOCX) [file pone.0192974.s002.docx]

# S1 Table: DFT energy value in each studied dihedral angle using 6-31g* or lacvp*for each fragment.

Tabulated data to build up the Conformational Energy Profiles for biaryl (**1-44**) and aryl carbonyl fragments (**45-59**)

4-pyrimidin-4-ylpyrimidine data was added in a separate table at the end of the file.

Rows coloured in blue corresponds to data obtained with a different QM basis set (lacvp*, used for haloderivatives) that the default one used in our study (6-31g*)

| Fragment | Dihedral Angle | Jaguar Relative Energy (kcal/mol) | Gas Phase Energy (eV) | QM Basis | QM Method |
| --- | --- | --- | --- | --- | --- |
| **1**  **1**  **1**  **1**  **1**  **1**  **1**  **1**  **1**  **1** | 0  20  40  60  80  100  120  140  160  180 | 2.09671  0.888857  0  1.03324  2.307407  2.299064  1.037139  0.000062  0.885797  2.099267 | -463.302257  -463.304181  -463.305598  -463.303951  -463.301921  -463.301934  -463.303945  -463.305598  -463.304186  -463.302252 | 6-31g*  6-31g*  6-31g*  6-31g*  6-31g*  6-31g*  6-31g*  6-31g*  6-31g*  6-31g* | DFT(b3lyp)  DFT(b3lyp)  DFT(b3lyp)  DFT(b3lyp)  DFT(b3lyp)  DFT(b3lyp)  DFT(b3lyp)  DFT(b3lyp)  DFT(b3lyp)  DFT(b3lyp) |
| **1**  **1**  **1**  **1**  **1**  **1**  **1**  **1**  **1**  **1** | 0  20  40  60  80  100  120  140  160  180 | 2.09115  0.769257  0  0.929926  2.247179  2.245672  0.911853  0.003355  0.769399  2.091031 | -463.292563  -463.29467  -463.295896  -463.294414  -463.292315  -463.292317  -463.294443  -463.295891  -463.29467  -463.292564 | lacvp*  lacvp*  lacvp*  lacvp*  lacvp*  lacvp*  lacvp*  lacvp*  lacvp*  lacvp* | DFT(b3lyp)  DFT(b3lyp)  DFT(b3lyp)  DFT(b3lyp)  DFT(b3lyp)  DFT(b3lyp)  DFT(b3lyp)  DFT(b3lyp)  DFT(b3lyp)  DFT(b3lyp) |
| **2**  **2**  **2**  **2**  **2**  **2**  **2**  **2**  **2**  **2**  **3**  **3**  **3**  **3**  **3**  **3**  **3**  **3**  **3**  **3**  **4**  **4**  **4**  **4**  **4**  **4**  **4**  **4**  **4**  **4** | 0  20  40  60  80  100  120  140  160  180  0  20  40  60  80  100  120  140  160  180  0  20  40  60  80  100  120  140  160  180 | 1.912647  0.66767  0  0.988327  2.361944  2.355669  0.989582  0.003765  0.665787  1.915157  1.976026  0.68524  0.001255  1.123241  2.407752  2.405242  0.990837  0  0.806349  1.976026  1.691137  0.530873  0  1.174697  2.692641  2.693896  1.152107  0.005648  0.52648  1.690509 | -577.825202  -577.827186  -577.82825  -577.826675  -577.824486  -577.824496  -577.826673  -577.828244  -577.827189  -577.825198  -555.546374  -555.548431  -555.549521  -555.547733  -555.545686  -555.54569  -555.547944  -555.549523  -555.548238  -555.546374  -670.070139  -670.071988  -670.072834  -670.070962  -670.068543  -670.068541  -670.070998  -670.072825  -670.071995  -670.07014 | 6-31g*  6-31g*  6-31g*  6-31g*  6-31g*  6-31g*  6-31g*  6-31g*  6-31g*  6-31g*  6-31g*  6-31g*  6-31g*  6-31g*  6-31g*  6-31g*  6-31g*  6-31g*  6-31g*  6-31g*  6-31g*  6-31g*  6-31g*  6-31g*  6-31g*  6-31g*  6-31g*  6-31g*  6-31g*  6-31g* | DFT(b3lyp)  DFT(b3lyp)  DFT(b3lyp)  DFT(b3lyp)  DFT(b3lyp)  DFT(b3lyp)  DFT(b3lyp)  DFT(b3lyp)  DFT(b3lyp)  DFT(b3lyp)  DFT(b3lyp)  DFT(b3lyp)  DFT(b3lyp)  DFT(b3lyp)  DFT(b3lyp)  DFT(b3lyp)  DFT(b3lyp)  DFT(b3lyp)  DFT(b3lyp)  DFT(b3lyp)  DFT(b3lyp)  DFT(b3lyp)  DFT(b3lyp)  DFT(b3lyp)  DFT(b3lyp)  DFT(b3lyp)  DFT(b3lyp)  DFT(b3lyp)  DFT(b3lyp)  DFT(b3lyp) |
| **5**  **5**  **5**  **5**  **5**  **5**  **5**  **5**  **5**  **5** | 0  20  40  60  80  100  120  140  160  180 | 0.134366  0  0.708045  2.596443  4.440618  4.445891  2.608041  0.693611  0.023286  0.148209 | -479.344965  -479.34518  -479.344051  -479.341042  -479.338103  -479.338095  -479.341023  -479.344074  -479.345142  -479.344943 | 6-31g*  6-31g*  6-31g*  6-31g*  6-31g*  6-31g*  6-31g*  6-31g*  6-31g*  6-31g* | DFT(b3lyp)  DFT(b3lyp)  DFT(b3lyp)  DFT(b3lyp)  DFT(b3lyp)  DFT(b3lyp)  DFT(b3lyp)  DFT(b3lyp)  DFT(b3lyp)  DFT(b3lyp) |
| **6**  **6**  **6**  **6**  **6**  **6**  **6**  **6**  **6**  **6** | 0  20  40  60  80  100  120  140  160  180 | 2.043169  0.761796  0.003138  0.896083  2.195654  2.182476  0.89671  0  0.747363  2.040659 | -479.33915  -479.341192  -479.342401  -479.340978  -479.338907  -479.338928  -479.340977  -479.342406  -479.341215  -479.339154 | 6-31g*  6-31g*  6-31g*  6-31g*  6-31g*  6-31g*  6-31g*  6-31g*  6-31g*  6-31g* | DFT(b3lyp)  DFT(b3lyp)  DFT(b3lyp)  DFT(b3lyp)  DFT(b3lyp)  DFT(b3lyp)  DFT(b3lyp)  DFT(b3lyp)  DFT(b3lyp)  DFT(b3lyp) |
| **7**  **7**  **7**  **7**  **7**  **7**  **7**  **7**  **7**  **7** | 0  20  40  60  80  100  120  140  160  180 | 1.657251  0.5566  0.004393  1.014682  2.40838  2.399594  1.124496  0  0.635667  1.654741 | -479.340269  -479.342023  -479.342903  -479.341293  -479.339072  -479.339086  -479.341118  -479.34291  -479.341897  -479.340273 | 6-31g*  6-31g*  6-31g*  6-31g*  6-31g*  6-31g*  6-31g*  6-31g*  6-31g*  6-31g* | DFT(b3lyp)  DFT(b3lyp)  DFT(b3lyp)  DFT(b3lyp)  DFT(b3lyp)  DFT(b3lyp)  DFT(b3lyp)  DFT(b3lyp)  DFT(b3lyp)  DFT(b3lyp) |
| **8** | 0 | 0.138679 | -593.868332 | 6-31g* | DFT(b3lyp) |
| **8** | 20 | 0.075301 | -593.868433 | 6-31g* | DFT(b3lyp) |
| **8** | 40 | 0.897338 | -593.867123 | 6-31g* | DFT(b3lyp) |
| **8** | 60 | 2.963725 | -593.86383 | 6-31g* | DFT(b3lyp) |
| **8** | 80 | 4.901473 | -593.860742 | 6-31g* | DFT(b3lyp) |
| **8** | 100 | 4.908375 | -593.860731 | 6-31g* | DFT(b3lyp) |
| **8** | 120 | 2.909132 | -593.863917 | 6-31g* | DFT(b3lyp) |
| **8** | 140 | 0.834587 | -593.867223 | 6-31g* | DFT(b3lyp) |
| **8** | 160 | 0 | -593.868553 | 6-31g* | DFT(b3lyp) |
| **8** | 180 | 0.024473 | -593.868514 | 6-31g* | DFT(b3lyp) |
| **9** | 0 | 0.090989 | -571.588899 | 6-31g* | DFT(b3lyp) |
| **9** | 20 | 0.03263 | -571.588992 | 6-31g* | DFT(b3lyp) |
| **9** | 40 | 0.791916 | -571.587782 | 6-31g* | DFT(b3lyp) |
| **9** | 60 | 2.771707 | -571.584627 | 6-31g* | DFT(b3lyp) |
| **9** | 80 | 4.617211 | -571.581686 | 6-31g* | DFT(b3lyp) |
| **9** | 100 | 4.617839 | -571.581685 | 6-31g* | DFT(b3lyp) |
| **9** | 120 | 2.717114 | -571.584714 | 6-31g* | DFT(b3lyp) |
| **9** | 140 | 0.764933 | -571.587825 | 6-31g* | DFT(b3lyp) |
| **9** | 160 | 0 | -571.589044 | 6-31g* | DFT(b3lyp) |
| **9** | 180 | 0.094126 | -571.588894 | 6-31g* | DFT(b3lyp) |
| **10** | 0 | 1.538096 | -441.216489 | 6-31g* | DFT(b3lyp) |
| **10** | 20 | 0.55931 | -441.218049 | 6-31g* | DFT(b3lyp) |
| **10** | 40 | 0 | -441.21894 | 6-31g* | DFT(b3lyp) |
| **10** | 60 | 0.975631 | -441.217385 | 6-31g* | DFT(b3lyp) |
| **10** | 80 | 2.083091 | -441.215621 | 6-31g* | DFT(b3lyp) |
| **10** | 100 | 2.077557 | -441.215629 | 6-31g* | DFT(b3lyp) |
| **10** | 120 | 0.973472 | -441.217389 | 6-31g* | DFT(b3lyp) |
| **10** | 140 | 0.004253 | -441.218933 | 6-31g* | DFT(b3lyp) |
| **10** | 160 | 0.557936 | -441.218051 | 6-31g* | DFT(b3lyp) |
| **10** | 180 | 1.538637 | -441.216488 | 6-31g* | DFT(b3lyp) |
| **11** | 0 | 1.034135 | -533.460404 | 6-31g* | DFT(b3lyp) |
| **11** | 20 | 0.141817 | -533.461835 | 6-31g* | DFT(b3lyp) |
| **11** | 40 | 0 | -533.462047 | 6-31g* | DFT(b3lyp) |
| **11** | 60 | 1.240585 | -533.460075 | 6-31g* | DFT(b3lyp) |
| **11** | 80 | 2.841361 | -533.457524 | 6-31g* | DFT(b3lyp) |
| **11** | 100 | 2.852656 | -533.457506 | 6-31g* | DFT(b3lyp) |
| **11** | 120 | 1.253763 | -533.460054 | 6-31g* | DFT(b3lyp) |
| **11** | 140 | 0 | -533.462052 | 6-31g* | DFT(b3lyp) |
| **11** | 160 | 0.141817 | -533.461826 | 6-31g* | DFT(b3lyp) |
| **11** | 180 | 1.032252 | -533.460407 | 6-31g* | DFT(b3lyp) |
| **12** | 0 | 1.881272 | -555.737847 | 6-31g* | DFT(b3lyp) |
| **12** | 20 | 0.683985 | -555.739755 | 6-31g* | DFT(b3lyp) |
| **12** | 40 | 0 | -555.740845 | 6-31g* | DFT(b3lyp) |
| **12** | 60 | 0.568523 | -555.739939 | 6-31g* | DFT(b3lyp) |
| **12** | 80 | 1.466489 | -555.738508 | 6-31g* | DFT(b3lyp) |
| **12** | 100 | 1.501002 | -555.738453 | 6-31g* | DFT(b3lyp) |
| **12** | 120 | 0.580446 | -555.73992 | 6-31g* | DFT(b3lyp) |
| **12** | 140 | 0.006903 | -555.740834 | 6-31g* | DFT(b3lyp) |
| **12** | 160 | 0.68775 | -555.739749 | 6-31g* | DFT(b3lyp) |
| **12** | 180 | 1.880644 | -555.737848 | 6-31g* | DFT(b3lyp) |
| **13** | 0 | 0.619979 | -457.25524 | 6-31g* | DFT(b3lyp) |
| **13** | 20 | 0.000628 | -457.256227 | 6-31g* | DFT(b3lyp) |
| **13** | 40 | 0.144327 | -457.255998 | 6-31g* | DFT(b3lyp) |
| **13** | 60 | 1.783381 | -457.253386 | 6-31g* | DFT(b3lyp) |
| **13** | 80 | 3.289402 | -457.250986 | 6-31g* | DFT(b3lyp) |
| **13** | 100 | 3.284382 | -457.250994 | 6-31g* | DFT(b3lyp) |
| **13** | 120 | 1.778988 | -457.253393 | 6-31g* | DFT(b3lyp) |
| **13** | 140 | 0.141817 | -457.256002 | 6-31g* | DFT(b3lyp) |
| **13** | 160 | 0 | -457.256228 | 6-31g* | DFT(b3lyp) |
| **13** | 180 | 0.619979 | -457.25524 | 6-31g* | DFT(b3lyp) |
| **14** | 0 | 0 | -457.262303 | 6-31g* | DFT(b3lyp) |
| **14** | 20 | 0.138591 | -457.262082 | 6-31g* | DFT(b3lyp) |
| **14** | 40 | 1.2303 | -457.260343 | 6-31g* | DFT(b3lyp) |
| **14** | 60 | 3.360896 | -457.256947 | 6-31g* | DFT(b3lyp) |
| **14** | 80 | 5.647553 | -457.253303 | 6-31g* | DFT(b3lyp) |
| **14** | 100 | 5.679514 | -457.253252 | 6-31g* | DFT(b3lyp) |
| **14** | 120 | 3.485101 | -457.256749 | 6-31g* | DFT(b3lyp) |
| **14** | 140 | 1.296223 | -457.260238 | 6-31g* | DFT(b3lyp) |
| **14** | 160 | 0.137781 | -457.262084 | 6-31g* | DFT(b3lyp) |
| **14** | 180 | 0.003166 | -457.262298 | 6-31g* | DFT(b3lyp) |
| **15** | 0 | 7.046926 | -502.608097 | 6-31g* | DFT(b3lyp) |
| **15** | 20 | 3.352153 | -502.613985 | 6-31g* | DFT(b3lyp) |
| **15** | 40 | 0.537775 | -502.61847 | 6-31g* | DFT(b3lyp) |
| **15** | 60 | 0 | -502.619327 | 6-31g* | DFT(b3lyp) |
| **15** | 80 | 0.426079 | -502.618648 | 6-31g* | DFT(b3lyp) |
| **15** | 100 | 0.448041 | -502.618613 | 6-31g* | DFT(b3lyp) |
| **15** | 120 | 0.016943 | -502.6193 | 6-31g* | DFT(b3lyp) |
| **15** | 140 | 0.373995 | -502.618731 | 6-31g* | DFT(b3lyp) |
| **15** | 160 | 3.021456 | -502.614512 | 6-31g* | DFT(b3lyp) |
| **15** | 180 | 7.046299 | -502.608098 | 6-31g* | DFT(b3lyp) |
| **15** | 0 | 7.040971 | -502.597718 | lacvp* | DFT(b3lyp) |
| **15** | 20 | 3.347463 | -502.603604 | lacvp* | DFT(b3lyp) |
| **15** | 40 | 0.529553 | -502.608095 | lacvp* | DFT(b3lyp) |
| **15** | 60 | 0 | -502.608939 | lacvp* | DFT(b3lyp) |
| **15** | 80 | 0.417848 | -502.608273 | lacvp* | DFT(b3lyp) |
| **15** | 100 | 0.431941 | -502.60825 | lacvp* | DFT(b3lyp) |
| **15** | 120 | 0.014883 | -502.608915 | lacvp* | DFT(b3lyp) |
| **15** | 140 | 0.369764 | -502.60835 | lacvp* | DFT(b3lyp) |
| **15** | 160 | 3.018033 | -502.604129 | lacvp* | DFT(b3lyp) |
| **15** | 180 | 7.037749 | -502.597723 | lacvp* | DFT(b3lyp) |
| **16** | 0 | 16.826654 | -541.908072 | 6-31g* | DFT(b3lyp) |
| **16** | 20 | 8.465096 | -541.921397 | 6-31g* | DFT(b3lyp) |
| **16** | 40 | 3.207198 | -541.929776 | 6-31g* | DFT(b3lyp) |
| **16** | 60 | 0.746108 | -541.933698 | 6-31g* | DFT(b3lyp) |
| **16** | 80 | 0 | -541.934887 | 6-31g* | DFT(b3lyp) |
| **16** | 100 | 0.003138 | -541.934882 | 6-31g* | DFT(b3lyp) |
| **16** | 120 | 0.72791 | -541.933727 | 6-31g* | DFT(b3lyp) |
| **16** | 140 | 3.148213 | -541.92987 | 6-31g* | DFT(b3lyp) |
| **16** | 160 | 8.605658 | -541.921173 | 6-31g* | DFT(b3lyp) |
| **16** | 180 | 16.969098 | -541.907845 | 6-31g* | DFT(b3lyp) |
| **17** | 0 | 19.526198 | -541.904036 | 6-31g* | DFT(b3lyp) |
| **17** | 20 | 10.058969 | -541.919123 | 6-31g* | DFT(b3lyp) |
| **17** | 40 | 3.763171 | -541.929156 | 6-31g* | DFT(b3lyp) |
| **17** | 60 | 0.819527 | -541.933847 | 6-31g* | DFT(b3lyp) |
| **17** | 80 | 0.061496 | -541.935055 | 6-31g* | DFT(b3lyp) |
| **17** | 100 | 0 | -541.935153 | 6-31g* | DFT(b3lyp) |
| **17** | 120 | 0.422941 | -541.934479 | 6-31g* | DFT(b3lyp) |
| **17** | 140 | 2.179339 | -541.93168 | 6-31g* | DFT(b3lyp) |
| **17** | 160 | 6.729407 | -541.924429 | 6-31g* | DFT(b3lyp) |
| **17** | 180 | 10.309973 | -541.918723 | 6-31g* | DFT(b3lyp) |
| **18** | 0 | 21.169644 | -581.217896 | 6-31g* | DFT(b3lyp) |
| **18** | 20 | 15.166265 | -581.227463 | 6-31g* | DFT(b3lyp) |
| **18** | 40 | 6.851771 | -581.240713 | 6-31g* | DFT(b3lyp) |
| **18** | 60 | 2.268445 | -581.248017 | 6-31g* | DFT(b3lyp) |
| **18** | 80 | 0.255396 | -581.251225 | 6-31g* | DFT(b3lyp) |
| **18** | 100 | 0.219001 | -581.251283 | 6-31g* | DFT(b3lyp) |
| **18** | 120 | 1.023467 | -581.250001 | 6-31g* | DFT(b3lyp) |
| **18** | 140 | 1.922688 | -581.248568 | 6-31g* | DFT(b3lyp) |
| **18** | 160 | 14.22312 | -581.229 | 6-31g* | DFT(b3lyp) |
| **18** | 180 | 27.428418 | -581.208 | 6-31g* | DFT(b3lyp) |
| **19** | 0 | 38.217181 | -620.507108 | 6-31g* | DFT(b3lyp) |
| **19** | 20 | 21.59133 | -620.533603 | 6-31g* | DFT(b3lyp) |
| **19** | 40 | 10.375861 | -620.551476 | 6-31g* | DFT(b3lyp) |
| **19** | 60 | 4.497985 | -620.560843 | 6-31g* | DFT(b3lyp) |
| **19** | 80 | 0.438001 | -620.567313 | 6-31g* | DFT(b3lyp) |
| **19** | 100 | 0.437374 | -620.567314 | 6-31g* | DFT(b3lyp) |
| **19** | 120 | 3.64771 | -620.562198 | 6-31g* | DFT(b3lyp) |
| **19** | 140 | 9.840596 | -620.552329 | 6-31g* | DFT(b3lyp) |
| **19** | 160 | 21.851118 | -620.533189 | 6-31g* | DFT(b3lyp) |
| **19** | 180 | 40.798125 | -620.502995 | 6-31g* | DFT(b3lyp) |
| **20** | 0 | 8.418033 | -541.918399 | 6-31g* | DFT(b3lyp) |
| **20** | 20 | 5.097883 | -541.92369 | 6-31g* | DFT(b3lyp) |
| **20** | 40 | 1.57442 | -541.929305 | 6-31g* | DFT(b3lyp) |
| **20** | 60 | 0 | -541.931814 | 6-31g* | DFT(b3lyp) |
| **20** | 80 | 0.183233 | -541.931522 | 6-31g* | DFT(b3lyp) |
| **20** | 100 | 0.333207 | -541.931283 | 6-31g* | DFT(b3lyp) |
| **20** | 120 | 0.107304 | -541.931643 | 6-31g* | DFT(b3lyp) |
| **20** | 140 | 0.674572 | -541.930739 | 6-31g* | DFT(b3lyp) |
| **20** | 160 | 3.66026 | -541.925981 | 6-31g* | DFT(b3lyp) |
| **20** | 180 | 8.407993 | -541.918415 | 6-31g* | DFT(b3lyp) |
| **21** | 0 | 12.377615 | -581.219745 | 6-31g* | DFT(b3lyp) |
| **21** | 20 | 7.069516 | -581.228204 | 6-31g* | DFT(b3lyp) |
| **21** | 40 | 2.96247 | -581.234749 | 6-31g* | DFT(b3lyp) |
| **21** | 60 | 0.820782 | -581.238162 | 6-31g* | DFT(b3lyp) |
| **21** | 80 | 0.038278 | -581.239409 | 6-31g* | DFT(b3lyp) |
| **21** | 100 | 0 | -581.23947 | 6-31g* | DFT(b3lyp) |
| **21** | 120 | 0.512047 | -581.238654 | 6-31g* | DFT(b3lyp) |
| **21** | 140 | 2.339354 | -581.235742 | 6-31g* | DFT(b3lyp) |
| **21** | 160 | 6.094367 | -581.229758 | 6-31g* | DFT(b3lyp) |
| **21** | 180 | 11.899453 | -581.220507 | 6-31g* | DFT(b3lyp) |
| **22** | 0 | 15.324397 | -620.521544 | 6-31g* | DFT(b3lyp) |
| **22** | 20 | 8.870467 | -620.531829 | 6-31g* | DFT(b3lyp) |
| **22** | 40 | 4.142187 | -620.539364 | 6-31g* | DFT(b3lyp) |
| **22** | 60 | 1.508532 | -620.543561 | 6-31g* | DFT(b3lyp) |
| **22** | 80 | 0.220256 | -620.545614 | 6-31g* | DFT(b3lyp) |
| **22** | 100 | 0 | -620.545965 | 6-31g* | DFT(b3lyp) |
| **22** | 120 | 0.892318 | -620.544543 | 6-31g* | DFT(b3lyp) |
| **22** | 140 | 3.057224 | -620.541093 | 6-31g* | DFT(b3lyp) |
| **22** | 160 | 6.972252 | -620.534854 | 6-31g* | DFT(b3lyp) |
| **22** | 180 | 15.34887 | -620.521505 | 6-31g* | DFT(b3lyp) |
| **23** | 0 | 2.602769 | -562.523226 | lacvp* | DFT(b3lyp) |
| **23** | 20 | 1.012245 | -562.525761 | lacvp* | DFT(b3lyp) |
| **23** | 40 | 0.005899 | -562.527364 | lacvp* | DFT(b3lyp) |
| **23** | 60 | 0.750957 | -562.526177 | lacvp* | DFT(b3lyp) |
| **23** | 80 | 1.981407 | -562.524216 | lacvp* | DFT(b3lyp) |
| **23** | 100 | 2.019994 | -562.524155 | lacvp* | DFT(b3lyp) |
| **23** | 120 | 0.815905 | -562.526073 | lacvp* | DFT(b3lyp) |
| **23** | 140 | 0 | -562.527374 | lacvp* | DFT(b3lyp) |
| **23** | 160 | 0.931452 | -562.525889 | lacvp* | DFT(b3lyp) |
| **23** | 180 | 2.603561 | -562.523225 | lacvp* | DFT(b3lyp) |
| **24** | 0 | 7.167733 | -922.872336 | lacvp* | DFT(b3lyp) |
| **24** | 20 | 3.467295 | -922.878233 | lacvp* | DFT(b3lyp) |
| **24** | 40 | 0.605461 | -922.882794 | lacvp* | DFT(b3lyp) |
| **24** | 60 | 0 | -922.883759 | lacvp* | DFT(b3lyp) |
| **24** | 80 | 0.576357 | -922.88284 | lacvp* | DFT(b3lyp) |
| **24** | 100 | 0.620293 | -922.88277 | lacvp* | DFT(b3lyp) |
| **24** | 120 | 0.053885 | -922.883673 | lacvp* | DFT(b3lyp) |
| **24** | 140 | 0.377244 | -922.883158 | lacvp* | DFT(b3lyp) |
| **24** | 160 | 3.024963 | -922.878938 | lacvp* | DFT(b3lyp) |
| **24** | 180 | 7.170849 | -922.872331 | lacvp* | DFT(b3lyp) |
| **25** | 0 | 8.185866 | -475.837029 | lacvp* | DFT(b3lyp) |
| **25** | 20 | 3.930922 | -475.84381 | lacvp* | DFT(b3lyp) |
| **25** | 40 | 0.825455 | -475.848759 | lacvp* | DFT(b3lyp) |
| **25** | 60 | 0 | -475.850074 | lacvp* | DFT(b3lyp) |
| **25** | 80 | 0.470928 | -475.849324 | lacvp* | DFT(b3lyp) |
| **25** | 100 | 0.518613 | -475.849248 | lacvp* | DFT(b3lyp) |
| **25** | 120 | 0.031806 | -475.850023 | lacvp* | DFT(b3lyp) |
| **25** | 140 | 0.548713 | -475.8492 | lacvp* | DFT(b3lyp) |
| **25** | 160 | 3.376107 | -475.844694 | lacvp* | DFT(b3lyp) |
| **25** | 180 | 8.182516 | -475.837034 | lacvp* | DFT(b3lyp) |
| **26** | 0 | 9.993729 | -474.052764 | lacvp* | DFT(b3lyp) |
| **26** | 20 | 4.603747 | -474.061353 | lacvp* | DFT(b3lyp) |
| **26** | 40 | 1.248163 | -474.066701 | lacvp* | DFT(b3lyp) |
| **26** | 60 | 0.021843 | -474.068655 | lacvp* | DFT(b3lyp) |
| **26** | 80 | 0.223481 | -474.068334 | lacvp* | DFT(b3lyp) |
| **26** | 100 | 0.261684 | -474.068273 | lacvp* | DFT(b3lyp) |
| **26** | 120 | 0 | -474.06869 | lacvp* | DFT(b3lyp) |
| **26** | 140 | 0.858125 | -474.067322 | lacvp* | DFT(b3lyp) |
| **26** | 160 | 3.861914 | -474.062536 | lacvp* | DFT(b3lyp) |
| **26** | 180 | 9.993601 | -474.052764 | lacvp* | DFT(b3lyp) |
| **27** | 0 | 7.242709 | -538.510598 | 6-31g* | DFT(b3lyp) |
| **27** | 20 | 2.723389 | -538.5178 | 6-31g* | DFT(b3lyp) |
| **27** | 40 | 0.145582 | -538.521908 | 6-31g* | DFT(b3lyp) |
| **27** | 60 | 0 | -538.52214 | 6-31g* | DFT(b3lyp) |
| **27** | 80 | 0.946284 | -538.520632 | 6-31g* | DFT(b3lyp) |
| **27** | 100 | 0.940636 | -538.520641 | 6-31g* | DFT(b3lyp) |
| **27** | 120 | 0.015688 | -538.522115 | 6-31g* | DFT(b3lyp) |
| **27** | 140 | 0.075929 | -538.522019 | 6-31g* | DFT(b3lyp) |
| **27** | 160 | 2.43348 | -538.518262 | 6-31g* | DFT(b3lyp) |
| **27** | 180 | 6.942132 | -538.511077 | 6-31g* | DFT(b3lyp) |
| **28** | 0 | 3.995977 | -577.818113 | 6-31g* | DFT(b3lyp) |
| **28** | 20 | 1.790911 | -577.821627 | 6-31g* | DFT(b3lyp) |
| **28** | 40 | 0.076556 | -577.824359 | 6-31g* | DFT(b3lyp) |
| **28** | 60 | 0.253514 | -577.824077 | 6-31g* | DFT(b3lyp) |
| **28** | 80 | 1.248743 | -577.822491 | 6-31g* | DFT(b3lyp) |
| **28** | 100 | 1.303336 | -577.822404 | 6-31g* | DFT(b3lyp) |
| **28** | 120 | 0.353915 | -577.823917 | 6-31g* | DFT(b3lyp) |
| **28** | 140 | 0 | -577.824481 | 6-31g* | DFT(b3lyp) |
| **28** | 160 | 1.606423 | -577.821921 | 6-31g* | DFT(b3lyp) |
| **28** | 180 | 3.995977 | -577.818113 | 6-31g* | DFT(b3lyp) |
| **29** | 0 | 4.636664 | -555.5387 | 6-31g* | DFT(b3lyp) |
| **29** | 20 | 1.997989 | -555.542905 | 6-31g* | DFT(b3lyp) |
| **29** | 40 | 0.056476 | -555.545999 | 6-31g* | DFT(b3lyp) |
| **29** | 60 | 0.259161 | -555.545676 | 6-31g* | DFT(b3lyp) |
| **29** | 80 | 1.349144 | -555.543939 | 6-31g* | DFT(b3lyp) |
| **29** | 100 | 1.379892 | -555.54389 | 6-31g* | DFT(b3lyp) |
| **29** | 120 | 0.325677 | -555.54557 | 6-31g* | DFT(b3lyp) |
| **29** | 140 | 0 | -555.546089 | 6-31g* | DFT(b3lyp) |
| **29** | 160 | 1.833581 | -555.543167 | 6-31g* | DFT(b3lyp) |
| **29** | 180 | 4.638547 | -555.538697 | 6-31g* | DFT(b3lyp) |
| **30** | 0 | 8.788264 | -800.3235 | 6-31g* | DFT(b3lyp) |
| **30** | 20 | 3.906871 | -800.331279 | 6-31g* | DFT(b3lyp) |
| **30** | 40 | 0.800074 | -800.33623 | 6-31g* | DFT(b3lyp) |
| **30** | 60 | 0 | -800.337505 | 6-31g* | DFT(b3lyp) |
| **30** | 80 | 0.466239 | -800.336762 | 6-31g* | DFT(b3lyp) |
| **30** | 100 | 0.513302 | -800.336687 | 6-31g* | DFT(b3lyp) |
| **30** | 120 | 0.03263 | -800.337453 | 6-31g* | DFT(b3lyp) |
| **30** | 140 | 0.500752 | -800.336707 | 6-31g* | DFT(b3lyp) |
| **30** | 160 | 3.335838 | -800.332189 | 6-31g* | DFT(b3lyp) |
| **30** | 180 | 8.52471 | -800.32392 | 6-31g* | DFT(b3lyp) |
| **31** | 0 | 2.664432 | -518.654196 | 6-31g* | DFT(b3lyp) |
| **31** | 20 | 1.179812 | -518.656562 | 6-31g* | DFT(b3lyp) |
| **31** | 40 | 0 | -518.658442 | 6-31g* | DFT(b3lyp) |
| **31** | 60 | 0.493881 | -518.657655 | 6-31g* | DFT(b3lyp) |
| **31** | 80 | 1.771734 | -518.655619 | 6-31g* | DFT(b3lyp) |
| **31** | 100 | 2.247969 | -518.65486 | 6-31g* | DFT(b3lyp) |
| **31** | 120 | 1.481145 | -518.656082 | 6-31g* | DFT(b3lyp) |
| **31** | 140 | 0.721548 | -518.657292 | 6-31g* | DFT(b3lyp) |
| **31** | 160 | 1.627351 | -518.655849 | 6-31g* | DFT(b3lyp) |
| **31** | 180 | 3.114479 | -518.653479 | 6-31g* | DFT(b3lyp) |
| **32** | 0 | 7.920445 | -495.375168 | 6-31g* | DFT(b3lyp) |
| **32** | 20 | 7.279496 | -495.376189 | 6-31g* | DFT(b3lyp) |
| **32** | 40 | 6.965185 | -495.37669 | 6-31g* | DFT(b3lyp) |
| **32** | 60 | 7.846351 | -495.375286 | 6-31g* | DFT(b3lyp) |
| **32** | 80 | 8.820266 | -495.373734 | 6-31g* | DFT(b3lyp) |
| **32** | 100 | 8.257902 | -495.37463 | 6-31g* | DFT(b3lyp) |
| **32** | 120 | 5.749147 | -495.378628 | 6-31g* | DFT(b3lyp) |
| **32** | 140 | 2.78041 | -495.383359 | 6-31g* | DFT(b3lyp) |
| **32** | 160 | 0.668263 | -495.386725 | 6-31g* | DFT(b3lyp) |
| **32** | 180 | 0 | -495.38779 | 6-31g* | DFT(b3lyp) |
| **33** | 0 | 8.993275 | -511.37667 | 6-31g* | DFT(b3lyp) |
| **33** | 20 | 8.276599 | -511.377812 | 6-31g* | DFT(b3lyp) |
| **33** | 40 | 7.745185 | -511.378659 | 6-31g* | DFT(b3lyp) |
| **33** | 60 | 8.399479 | -511.377617 | 6-31g* | DFT(b3lyp) |
| **33** | 80 | 9.17809 | -511.376376 | 6-31g* | DFT(b3lyp) |
| **33** | 100 | 8.323765 | -511.377737 | 6-31g* | DFT(b3lyp) |
| **33** | 120 | 5.647459 | -511.382002 | 6-31g* | DFT(b3lyp) |
| **33** | 140 | 2.650644 | -511.386778 | 6-31g* | DFT(b3lyp) |
| **33** | 160 | 0.640917 | -511.389981 | 6-31g* | DFT(b3lyp) |
| **33** | 180 | 0 | -511.391002 | 6-31g* | DFT(b3lyp) |
| **34** | 0 | 7.683861 | -511.415128 | 6-31g* | DFT(b3lyp) |
| **34** | 20 | 7.189234 | -511.415917 | 6-31g* | DFT(b3lyp) |
| **34** | 40 | 7.011848 | -511.416199 | 6-31g* | DFT(b3lyp) |
| **34** | 60 | 7.956708 | -511.414694 | 6-31g* | DFT(b3lyp) |
| **34** | 80 | 8.978567 | -511.413065 | 6-31g* | DFT(b3lyp) |
| **34** | 100 | 8.391681 | -511.414 | 6-31g* | DFT(b3lyp) |
| **34** | 120 | 5.888803 | -511.417989 | 6-31g* | DFT(b3lyp) |
| **34** | 140 | 2.889423 | -511.422769 | 6-31g* | DFT(b3lyp) |
| **34** | 160 | 0.728547 | -511.426212 | 6-31g* | DFT(b3lyp) |
| **34** | 180 | 0 | -511.427373 | 6-31g* | DFT(b3lyp) |
| **35** | 0 | 6.996585 | -511.408352 | 6-31g* | DFT(b3lyp) |
| **35** | 20 | 6.394832 | -511.409311 | 6-31g* | DFT(b3lyp) |
| **35** | 40 | 6.153964 | -511.409695 | 6-31g* | DFT(b3lyp) |
| **35** | 60 | 7.104169 | -511.408181 | 6-31g* | DFT(b3lyp) |
| **35** | 80 | 8.203404 | -511.406429 | 6-31g* | DFT(b3lyp) |
| **35** | 100 | 7.702488 | -511.407227 | 6-31g* | DFT(b3lyp) |
| **35** | 120 | 5.345877 | -511.410983 | 6-31g* | DFT(b3lyp) |
| **35** | 140 | 2.535049 | -511.415462 | 6-31g* | DFT(b3lyp) |
| **35** | 160 | 0.617504 | -511.418518 | 6-31g* | DFT(b3lyp) |
| **35** | 180 | 0 | -511.419502 | 6-31g* | DFT(b3lyp) |
| **36** | 0 | 5.666716 | -493.164183 | 6-31g* | DFT(b3lyp) |
| **36** | 20 | 5.63335 | -493.164236 | 6-31g* | DFT(b3lyp) |
| **36** | 40 | 6.028188 | -493.163607 | 6-31g* | DFT(b3lyp) |
| **36** | 60 | 7.16461 | -493.161796 | 6-31g* | DFT(b3lyp) |
| **36** | 80 | 8.268526 | -493.160037 | 6-31g* | DFT(b3lyp) |
| **36** | 100 | 7.960436 | -493.160528 | 6-31g* | DFT(b3lyp) |
| **36** | 120 | 5.853701 | -493.163885 | 6-31g* | DFT(b3lyp) |
| **36** | 140 | 3.078377 | -493.168308 | 6-31g* | DFT(b3lyp) |
| **36** | 160 | 0.845161 | -493.171867 | 6-31g* | DFT(b3lyp) |
| **36** | 180 | 0 | -493.173213 | 6-31g* | DFT(b3lyp) |
| **37** | 0 | 6.380729 | -512.62218 | 6-31g* | DFT(b3lyp) |
| **37** | 20 | 6.333378 | -512.622255 | 6-31g* | DFT(b3lyp) |
| **37** | 40 | 6.825916 | -512.621471 | 6-31g* | DFT(b3lyp) |
| **37** | 60 | 8.144971 | -512.619368 | 6-31g* | DFT(b3lyp) |
| **37** | 80 | 9.335623 | -512.617471 | 6-31g* | DFT(b3lyp) |
| **37** | 100 | 8.836508 | -512.618266 | 6-31g* | DFT(b3lyp) |
| **37** | 120 | 6.343144 | -512.62224 | 6-31g* | DFT(b3lyp) |
| **37** | 140 | 3.249602 | -512.62717 | 6-31g* | DFT(b3lyp) |
| **37** | 160 | 0.872527 | -512.630958 | 6-31g* | DFT(b3lyp) |
| **37** | 180 | 0 | -512.632348 | 6-31g* | DFT(b3lyp) |
| **38** | 0 | 6.200226 | -816.139989 | 6-31g* | DFT(b3lyp) |
| **38** | 20 | 5.983997 | -816.140334 | 6-31g* | DFT(b3lyp) |
| **38** | 40 | 6.183512 | -816.140016 | 6-31g* | DFT(b3lyp) |
| **38** | 60 | 7.258433 | -816.138303 | 6-31g* | DFT(b3lyp) |
| **38** | 80 | 8.367373 | -816.136536 | 6-31g* | DFT(b3lyp) |
| **38** | 100 | 8.099568 | -816.136963 | 6-31g* | DFT(b3lyp) |
| **38** | 120 | 5.949653 | -816.140389 | 6-31g* | DFT(b3lyp) |
| **38** | 140 | 3.092172 | -816.144942 | 6-31g* | DFT(b3lyp) |
| **38** | 160 | 0.83397 | -816.148541 | 6-31g* | DFT(b3lyp) |
| **38** | 180 | 0 | -816.14987 | 6-31g* | DFT(b3lyp) |
| **39** | 0 | 5.42701 | -578.569511 | 6-31g* | DFT(b3lyp) |
| **39** | 20 | 4.182842 | -578.571494 | 6-31g* | DFT(b3lyp) |
| **39** | 40 | 3.150831 | -578.573138 | 6-31g* | DFT(b3lyp) |
| **39** | 60 | 3.629436 | -578.572376 | 6-31g* | DFT(b3lyp) |
| **39** | 80 | 4.64507 | -578.570757 | 6-31g* | DFT(b3lyp) |
| **39** | 100 | 4.502354 | -578.570985 | 6-31g* | DFT(b3lyp) |
| **39** | 120 | 2.735046 | -578.573801 | 6-31g* | DFT(b3lyp) |
| **39** | 140 | 0.798368 | -578.576887 | 6-31g* | DFT(b3lyp) |
| **39** | 160 | 0 | -578.57816 | 6-31g* | DFT(b3lyp) |
| **39** | 180 | 0.126196 | -578.577959 | 6-31g* | DFT(b3lyp) |
| **40** | 0 | 9.673812 | -661.754488 | 6-31g* | DFT(b3lyp) |
| **40** | 20 | 3.920766 | -661.763656 | 6-31g* | DFT(b3lyp) |
| **40** | 40 | 0.473612 | -661.769149 | 6-31g* | DFT(b3lyp) |
| **40** | 60 | 0.145249 | -661.769673 | 6-31g* | DFT(b3lyp) |
| **40** | 80 | 1.452359 | -661.76759 | 6-31g* | DFT(b3lyp) |
| **40** | 100 | 1.768743 | -661.767085 | 6-31g* | DFT(b3lyp) |
| **40** | 120 | 0.670919 | -661.768835 | 6-31g* | DFT(b3lyp) |
| **40** | 140 | 0 | -661.769904 | 6-31g* | DFT(b3lyp) |
| **40** | 160 | 1.170642 | -661.768039 | 6-31g* | DFT(b3lyp) |
| **40** | 180 | 3.517246 | -661.764299 | 6-31g* | DFT(b3lyp) |
| **41** | 0 | 0.168336 | -457.253299 | 6-31g* | DFT(b3lyp) |
| **41** | 20 | 0 | -457.253567 | 6-31g* | DFT(b3lyp) |
| **41** | 40 | 0.455529 | -457.252841 | 6-31g* | DFT(b3lyp) |
| **41** | 60 | 1.87529 | -457.250579 | 6-31g* | DFT(b3lyp) |
| **41** | 80 | 3.349273 | -457.24823 | 6-31g* | DFT(b3lyp) |
| **41** | 100 | 3.354052 | -457.248222 | 6-31g* | DFT(b3lyp) |
| **41** | 120 | 1.893953 | -457.250549 | 6-31g* | DFT(b3lyp) |
| **41** | 140 | 0.474396 | -457.252811 | 6-31g* | DFT(b3lyp) |
| **41** | 160 | 0.002557 | -457.253563 | 6-31g* | DFT(b3lyp) |
| **41** | 180 | 0.158837 | -457.253314 | 6-31g* | DFT(b3lyp) |
| **42** | 0 | 7.750376 | -473.287393 | 6-31g* | DFT(b3lyp) |
| **42** | 20 | 7.369394 | -473.288 | 6-31g* | DFT(b3lyp) |
| **42** | 40 | 7.387752 | -473.287971 | 6-31g* | DFT(b3lyp) |
| **42** | 60 | 8.381408 | -473.286388 | 6-31g* | DFT(b3lyp) |
| **42** | 80 | 9.446882 | -473.28469 | 6-31g* | DFT(b3lyp) |
| **42** | 100 | 8.518138 | -473.28617 | 6-31g* | DFT(b3lyp) |
| **42** | 120 | 5.770362 | -473.290549 | 6-31g* | DFT(b3lyp) |
| **42** | 140 | 2.812356 | -473.295262 | 6-31g* | DFT(b3lyp) |
| **42** | 160 | 0.713481 | -473.298607 | 6-31g* | DFT(b3lyp) |
| **42** | 180 | 0 | -473.299744 | 6-31g* | DFT(b3lyp) |
| **43** | 0 | 1.906944 | -540.445241 | 6-31g* | DFT(b3lyp) |
| **43** | 20 | 0.730288 | -540.447117 | 6-31g* | DFT(b3lyp) |
| **43** | 40 | 0 | -540.44828 | 6-31g* | DFT(b3lyp) |
| **43** | 60 | 0.620083 | -540.447292 | 6-31g* | DFT(b3lyp) |
| **43** | 80 | 1.867583 | -540.445304 | 6-31g* | DFT(b3lyp) |
| **43** | 100 | 1.844452 | -540.445341 | 6-31g* | DFT(b3lyp) |
| **43** | 120 | 0.823084 | -540.446969 | 6-31g* | DFT(b3lyp) |
| **43** | 140 | 0.032996 | -540.448228 | 6-31g* | DFT(b3lyp) |
| **43** | 160 | 0.483941 | -540.447509 | 6-31g* | DFT(b3lyp) |
| **43** | 180 | 1.909258 | -540.445238 | 6-31g* | DFT(b3lyp) |
| **44** | 0 | 5.591247 | -556.475069 | 6-31g* | DFT(b3lyp) |
| **44** | 20 | 4.408224 | -556.476954 | 6-31g* | DFT(b3lyp) |
| **44** | 40 | 3.260416 | -556.478783 | 6-31g* | DFT(b3lyp) |
| **44** | 60 | 3.330643 | -556.478671 | 6-31g* | DFT(b3lyp) |
| **44** | 80 | 4.068882 | -556.477495 | 6-31g* | DFT(b3lyp) |
| **44** | 100 | 3.734389 | -556.478028 | 6-31g* | DFT(b3lyp) |
| **44** | 120 | 2.221505 | -556.480439 | 6-31g* | DFT(b3lyp) |
| **44** | 140 | 0.644173 | -556.482952 | 6-31g* | DFT(b3lyp) |
| **44** | 160 | 0 | -556.483979 | 6-31g* | DFT(b3lyp) |
| **44** | 180 | 0.090872 | -556.483834 | 6-31g* | DFT(b3lyp) |
| **45** | 0 | 0 | -384.895738 | 6-31g* | DFT(b3lyp) |
| **45** | 20 | 0.672463 | -384.894666 | 6-31g* | DFT(b3lyp) |
| **45** | 40 | 2.555764 | -384.891665 | 6-31g* | DFT(b3lyp) |
| **45** | 60 | 4.901791 | -384.887926 | 6-31g* | DFT(b3lyp) |
| **45** | 80 | 6.716123 | -384.885035 | 6-31g* | DFT(b3lyp) |
| **45** | 100 | 6.653774 | -384.885134 | 6-31g* | DFT(b3lyp) |
| **45** | 120 | 4.694072 | -384.888257 | 6-31g* | DFT(b3lyp) |
| **45** | 140 | 2.390079 | -384.891929 | 6-31g* | DFT(b3lyp) |
| **45** | 160 | 0.617834 | -384.894753 | 6-31g* | DFT(b3lyp) |
| **45** | 180 | 0.001169 | -384.895736 | 6-31g* | DFT(b3lyp) |
| **46** | 0 | 6.492125 | -400.924423 | 6-31g* | DFT(b3lyp) |
| **46** | 20 | 6.736982 | -400.924033 | 6-31g* | DFT(b3lyp) |
| **46** | 40 | 7.563382 | -400.922716 | 6-31g* | DFT(b3lyp) |
| **46** | 60 | 8.519868 | -400.921191 | 6-31g* | DFT(b3lyp) |
| **46** | 80 | 9.009893 | -400.92041 | 6-31g* | DFT(b3lyp) |
| **46** | 100 | 7.827595 | -400.922295 | 6-31g* | DFT(b3lyp) |
| **46** | 120 | 5.26079 | -400.926385 | 6-31g* | DFT(b3lyp) |
| **46** | 140 | 2.587603 | -400.930645 | 6-31g* | DFT(b3lyp) |
| **46** | 160 | 0.703527 | -400.933647 | 6-31g* | DFT(b3lyp) |
| **46** | 180 | 0 | -400.934769 | 6-31g* | DFT(b3lyp) |
| **47** | 0 | 3.794344 | -484.122009 | 6-31g* | DFT(b3lyp) |
| **47** | 20 | 3.981206 | -484.121711 | 6-31g* | DFT(b3lyp) |
| **47** | 40 | 4.760496 | -484.120469 | 6-31g* | DFT(b3lyp) |
| **47** | 60 | 5.815581 | -484.118787 | 6-31g* | DFT(b3lyp) |
| **47** | 80 | 6.590485 | -484.117553 | 6-31g* | DFT(b3lyp) |
| **47** | 100 | 5.820442 | -484.11878 | 6-31g* | DFT(b3lyp) |
| **47** | 120 | 3.962132 | -484.121741 | 6-31g* | DFT(b3lyp) |
| **47** | 140 | 2.014363 | -484.124845 | 6-31g* | DFT(b3lyp) |
| **47** | 160 | 0.483586 | -484.127285 | 6-31g* | DFT(b3lyp) |
| **47** | 180 | 0 | -484.128055 | 6-31g* | DFT(b3lyp) |
| **48** | 0 | 0.579318 | -400.949886 | 6-31g* | DFT(b3lyp) |
| **48** | 20 | 0.002703 | -400.950804 | 6-31g* | DFT(b3lyp) |
| **48** | 40 | 0.840534 | -400.949469 | 6-31g* | DFT(b3lyp) |
| **48** | 60 | 2.752246 | -400.946423 | 6-31g* | DFT(b3lyp) |
| **48** | 80 | 4.499325 | -400.943639 | 6-31g* | DFT(b3lyp) |
| **48** | 100 | 4.368984 | -400.943846 | 6-31g* | DFT(b3lyp) |
| **48** | 120 | 2.505645 | -400.946816 | 6-31g* | DFT(b3lyp) |
| **48** | 140 | 0.706347 | -400.949683 | 6-31g* | DFT(b3lyp) |
| **48** | 160 | 0 | -400.950809 | 6-31g* | DFT(b3lyp) |
| **48** | 180 | 0.577943 | -400.949888 | 6-31g* | DFT(b3lyp) |
| **49** | 0 | 11.192227 | -416.977746 | 6-31g* | DFT(b3lyp) |
| **49** | 20 | 9.779996 | -416.979997 | 6-31g* | DFT(b3lyp) |
| **49** | 40 | 9.468521 | -416.980493 | 6-31g* | DFT(b3lyp) |
| **49** | 60 | 10.097488 | -416.979491 | 6-31g* | DFT(b3lyp) |
| **49** | 80 | 10.660064 | -416.978594 | 6-31g* | DFT(b3lyp) |
| **49** | 100 | 9.623332 | -416.980247 | 6-31g* | DFT(b3lyp) |
| **49** | 120 | 6.718734 | -416.984875 | 6-31g* | DFT(b3lyp) |
| **49** | 140 | 3.305799 | -416.990314 | 6-31g* | DFT(b3lyp) |
| **49** | 160 | 0.828551 | -416.994262 | 6-31g* | DFT(b3lyp) |
| **49** | 180 | 0 | -416.995582 | 6-31g* | DFT(b3lyp) |
| **50** | 0 | 7.460334 | -500.174574 | 6-31g* | DFT(b3lyp) |
| **50** | 20 | 5.744842 | -500.177308 | 6-31g* | DFT(b3lyp) |
| **50** | 40 | 5.264639 | -500.178073 | 6-31g* | DFT(b3lyp) |
| **50** | 60 | 5.840573 | -500.177156 | 6-31g* | DFT(b3lyp) |
| **50** | 80 | 6.583835 | -500.175971 | 6-31g* | DFT(b3lyp) |
| **50** | 100 | 6.317929 | -500.176395 | 6-31g* | DFT(b3lyp) |
| **50** | 120 | 4.446916 | -500.179376 | 6-31g* | DFT(b3lyp) |
| **50** | 140 | 2.038812 | -500.183214 | 6-31g* | DFT(b3lyp) |
| **50** | 160 | 0.482818 | -500.185694 | 6-31g* | DFT(b3lyp) |
| **50** | 180 | 0 | -500.186463 | 6-31g* | DFT(b3lyp) |
| **51** | 0 | 0.610566 | -440.260452 | 6-31g* | DFT(b3lyp) |
| **51** | 20 | 0 | -440.261425 | 6-31g* | DFT(b3lyp) |
| **51** | 40 | 0.577936 | -440.260504 | 6-31g* | DFT(b3lyp) |
| **51** | 60 | 2.287898 | -440.257779 | 6-31g* | DFT(b3lyp) |
| **51** | 80 | 3.908754 | -440.255196 | 6-31g* | DFT(b3lyp) |
| **51** | 100 | 3.794547 | -440.255378 | 6-31g* | DFT(b3lyp) |
| **51** | 120 | 2.069525 | -440.258127 | 6-31g* | DFT(b3lyp) |
| **51** | 140 | 0.519577 | -440.260597 | 6-31g* | DFT(b3lyp) |
| **51** | 160 | 0.070281 | -440.261313 | 6-31g* | DFT(b3lyp) |
| **51** | 180 | 0.582956 | -440.260496 | 6-31g* | DFT(b3lyp) |
| **52** | 0 | 3.85165 | -479.558609 | 6-31g* | DFT(b3lyp) |
| **52** | 20 | 0.955696 | -479.563224 | 6-31g* | DFT(b3lyp) |
| **52** | 40 | 0 | -479.564747 | 6-31g* | DFT(b3lyp) |
| **52** | 60 | 0.336972 | -479.56377 | 6-31g* | DFT(b3lyp) |
| **52** | 80 | 1.412523 | -479.562496 | 6-31g* | DFT(b3lyp) |
| **52** | 100 | 1.412523 | -479.56202 | 6-31g* | DFT(b3lyp) |
| **52** | 120 | 0.336972 | -479.56421 | 6-31g* | DFT(b3lyp) |
| **52** | 140 | 0.079694 | -479.56462 | 6-31g* | DFT(b3lyp) |
| **52** | 160 | 0.955696 | -479.562347 | 6-31g* | DFT(b3lyp) |
| **52** | 180 | 3.85165 | -479.557605 | 6-31g* | DFT(b3lyp) |
| **53** | 0 | 2.429087 | -440.261932 | 6-31g* | DFT(b3lyp) |
| **53** | 20 | 1.479666 | -440.263445 | 6-31g* | DFT(b3lyp) |
| **53** | 40 | 1.553085 | -440.263328 | 6-31g* | DFT(b3lyp) |
| **53** | 60 | 2.419047 | -440.261948 | 6-31g* | DFT(b3lyp) |
| **53** | 80 | 2.95243 | -440.261098 | 6-31g* | DFT(b3lyp) |
| **53** | 100 | 2.372612 | -440.262022 | 6-31g* | DFT(b3lyp) |
| **53** | 120 | 0.972639 | -440.264253 | 6-31g* | DFT(b3lyp) |
| **53** | 140 | 0 | -440.265803 | 6-31g* | DFT(b3lyp) |
| **53** | 160 | 0.228413 | -440.265439 | 6-31g* | DFT(b3lyp) |
| **53** | 180 | 1.785263 | -440.262958 | 6-31g* | DFT(b3lyp) |
| **54** | 0 | 5.165027 | -479.570658 | 6-31g* | DFT(b3lyp) |
| **54** | 20 | 2.482426 | -479.574933 | 6-31g* | DFT(b3lyp) |
| **54** | 40 | 0.663905 | -479.577831 | 6-31g* | DFT(b3lyp) |
| **54** | 60 | 0.040788 | -479.578824 | 6-31g* | DFT(b3lyp) |
| **54** | 80 | 0.026983 | -479.578846 | 6-31g* | DFT(b3lyp) |
| **54** | 100 | 0 | -479.578889 | 6-31g* | DFT(b3lyp) |
| **54** | 120 | 0.075301 | -479.578769 | 6-31g* | DFT(b3lyp) |
| **54** | 140 | 0.772464 | -479.577658 | 6-31g* | DFT(b3lyp) |
| **54** | 160 | 2.550824 | -479.574824 | 6-31g* | DFT(b3lyp) |
| **54** | 180 | 4.828682 | -479.571194 | 6-31g* | DFT(b3lyp) |
| **55** | 0 | 6.552449 | -518.870879 | 6-31g* | DFT(b3lyp) |
| **55** | 20 | 2.531371 | -518.877287 | 6-31g* | DFT(b3lyp) |
| **55** | 40 | 1.908882 | -518.878279 | 6-31g* | DFT(b3lyp) |
| **55** | 60 | 0 | -518.881321 | 6-31g* | DFT(b3lyp) |
| **55** | 80 | 1.701177 | -518.87861 | 6-31g* | DFT(b3lyp) |
| **55** | 100 | 2.391437 | -518.87751 | 6-31g* | DFT(b3lyp) |
| **55** | 120 | 1.088728 | -518.879586 | 6-31g* | DFT(b3lyp) |
| **55** | 140 | 2.307351 | -518.877644 | 6-31g* | DFT(b3lyp) |
| **55** | 160 | 5.401597 | -518.872713 | 6-31g* | DFT(b3lyp) |
| **55** | 180 | 10.437357 | -518.864688 | 6-31g* | DFT(b3lyp) |
| **56** | 0 | 14.450277 | -558.174778 | 6-31g* | DFT(b3lyp) |
| **56** | 20 | 7.719616 | -558.185504 | 6-31g* | DFT(b3lyp) |
| **56** | 40 | 3.059106 | -558.192931 | 6-31g* | DFT(b3lyp) |
| **56** | 60 | 0.663277 | -558.196749 | 6-31g* | DFT(b3lyp) |
| **56** | 80 | 0 | -558.197806 | 6-31g* | DFT(b3lyp) |
| **56** | 100 | 0.057731 | -558.197714 | 6-31g* | DFT(b3lyp) |
| **56** | 120 | 1.129516 | -558.196006 | 6-31g* | DFT(b3lyp) |
| **56** | 140 | 3.923814 | -558.191553 | 6-31g* | DFT(b3lyp) |
| **56** | 160 | 8.665272 | -558.183997 | 6-31g* | DFT(b3lyp) |
| **56** | 180 | 15.057706 | -558.17381 | 6-31g* | DFT(b3lyp) |
| **57** | 0 | 0.003196 | -440.264905 | 6-31g* | DFT(b3lyp) |
| **57** | 20 | 0.341778 | -440.264366 | 6-31g* | DFT(b3lyp) |
| **57** | 40 | 1.568091 | -440.262411 | 6-31g* | DFT(b3lyp) |
| **57** | 60 | 3.108595 | -440.259956 | 6-31g* | DFT(b3lyp) |
| **57** | 80 | 4.510635 | -440.257722 | 6-31g* | DFT(b3lyp) |
| **57** | 100 | 4.728029 | -440.257376 | 6-31g* | DFT(b3lyp) |
| **57** | 120 | 3.38797 | -440.259511 | 6-31g* | DFT(b3lyp) |
| **57** | 140 | 1.831964 | -440.261991 | 6-31g* | DFT(b3lyp) |
| **57** | 160 | 0.519185 | -440.264083 | 6-31g* | DFT(b3lyp) |
| **57** | 180 | 0 | -440.26491 | 6-31g* | DFT(b3lyp) |
| **58** | 0 | 8.975092 | -456.298166 | 6-31g* | DFT(b3lyp) |
| **58** | 20 | 8.948918 | -456.298208 | 6-31g* | DFT(b3lyp) |
| **58** | 40 | 9.098058 | -456.29797 | 6-31g* | DFT(b3lyp) |
| **58** | 60 | 9.763161 | -456.29691 | 6-31g* | DFT(b3lyp) |
| **58** | 80 | 9.633511 | -456.297117 | 6-31g* | DFT(b3lyp) |
| **58** | 100 | 7.153479 | -456.301069 | 6-31g* | DFT(b3lyp) |
| **58** | 120 | 4.204531 | -456.305768 | 6-31g* | DFT(b3lyp) |
| **58** | 140 | 1.813737 | -456.309578 | 6-31g* | DFT(b3lyp) |
| **58** | 160 | 0.389602 | -456.311848 | 6-31g* | DFT(b3lyp) |
| **58** | 180 | 0 | -456.312469 | 6-31g* | DFT(b3lyp) |
| **59** | 0 | 9.836236 | -539.483241 | 6-31g* | DFT(b3lyp) |
| **59** | 20 | 7.935212 | -539.486271 | 6-31g* | DFT(b3lyp) |
| **59** | 40 | 5.882996 | -539.489541 | 6-31g* | DFT(b3lyp) |
| **59** | 60 | 5.343513 | -539.490401 | 6-31g* | DFT(b3lyp) |
| **59** | 80 | 6.287306 | -539.488897 | 6-31g* | DFT(b3lyp) |
| **59** | 100 | 5.282939 | -539.490497 | 6-31g* | DFT(b3lyp) |
| **59** | 120 | 3.3479 | -539.493581 | 6-31g* | DFT(b3lyp) |
| **59** | 140 | 1.51009 | -539.49651 | 6-31g* | DFT(b3lyp) |
| **59** | 160 | 0.28572 | -539.498461 | 6-31g* | DFT(b3lyp) |
| **59** | 180 | 0 | -539.498916 | 6-31g* | DFT(b3lyp) |

Addition of calculated data for 4-pyrimidin-4-yl-pyrimidine, a non-basic fragment in response to our reviewers comments about the influence of the basicity in the Energy Profile of the fragment.

| Fragment | Dihedral Angle | Jaguar Relative Energy (kcal/mol) | Gas Phase Energy (eV) | QM Basis | QM Method |
| --- | --- | --- | --- | --- | --- |
| 4-pyrimidin-4-yl-pyrimidine | 0 | 7.462466 | -527.453435 | 6-31g* | DFT(b3lyp) |
| 4-pyrimidin-4-yl-pyrimidine | 20 | 7.025831 | -527.454131 | 6-31g* | DFT(b3lyp) |
| 4-pyrimidin-4-yl-pyrimidine | 40 | 6.892632 | -527.454343 | 6-31g* | DFT(b3lyp) |
| 4-pyrimidin-4-yl-pyrimidine | 60 | 7.766395 | -527.45295 | 6-31g* | DFT(b3lyp) |
| 4-pyrimidin-4-yl-pyrimidine | 80 | 8.703987 | -527.451456 | 6-31g* | DFT(b3lyp) |
| 4-pyrimidin-4-yl-pyrimidine | 100 | 8.096764 | -527.452424 | 6-31g* | DFT(b3lyp) |
| 4-pyrimidin-4-yl-pyrimidine | 120 | 5.726865 | -527.456201 | 6-31g* | DFT(b3lyp) |
| 4-pyrimidin-4-yl-pyrimidine | 140 | 2.822939 | -527.460828 | 6-31g* | DFT(b3lyp) |
| 4-pyrimidin-4-yl-pyrimidine | 160 | 0.719752 | -527.46418 | 6-31g* | DFT(b3lyp) |
| 4-pyrimidin-4-yl-pyrimidine | 180 | 0 | -527.465327 | 6-31g* | DFT(b3lyp) |
