# Supplementary material for: Rapid assessment of conformational preferences in biaryl and aryl carbonyl fragments
Source: PLoS One. 2018 Mar 14;13(3):e0192974. doi: 10.1371/journal.pone.0192974 (PMC5851544; doi:10.1371/journal.pone.0192974)
Supplement: S2 Table — (DOCX) [file pone.0192974.s003.docx]

# S2 Table: DFT energy value in each studied dihedral angle using cc-pvtz(-f) for biaryl (1, 4, 5-7, 15, 23-25, 31, 32, 42, 44) and aryl carbonyl fragments (45-47, 50) and cc-pvtz-pp(-f) for 26.

Tabulated data to build up the Conformational Energy Profiles for biaryl fragments (**1, 4, 5-7, 15, 23-25, 26, 31, 32, 42, 44**) and acetophenone **45-47, 49, 50** with a more complex basis sets.

| Fragment | Dihedral Angle | Jaguar Relative Energy (kcal/mol) | QM Basis | QM Method |
| --- | --- | --- | --- | --- |
| **1**  **1**  **1**  **1**  **1**  **1**  **1**  **1**  **1**  **1** | 0  20  40  60  80  100  120  140  160  180 | 1.896  0.9  0.002  0.837  1.93  1.929  0.836  0  0.901  1.896 | cc-pvtz(-f)  cc-pvtz(-f)  cc-pvtz(-f)  cc-pvtz(-f)  cc-pvtz(-f)  cc-pvtz(-f)  cc-pvtz(-f)  cc-pvtz(-f)  cc-pvtz(-f)  cc-pvtz(-f) | DFT(b3lyp)  DFT(b3lyp)  DFT(b3lyp)  DFT(b3lyp)  DFT(b3lyp)  DFT(b3lyp)  DFT(b3lyp)  DFT(b3lyp)  DFT(b3lyp)  DFT(b3lyp) |
| **4**  **4**  **4**  **4**  **4**  **4**  **4**  **4**  **4**  **4** | 0  20  40  60  80  100  120  140  160  180 | 1.488  0.534  0  1.037  2.388  2.381  1.045  0.007  0.546  1.483 | cc-pvtz(-f)  cc-pvtz(-f)  cc-pvtz(-f)  cc-pvtz(-f)  cc-pvtz(-f)  cc-pvtz(-f)  cc-pvtz(-f)  cc-pvtz(-f)  cc-pvtz(-f)  cc-pvtz(-f) | DFT(b3lyp)  DFT(b3lyp)  DFT(b3lyp)  DFT(b3lyp)  DFT(b3lyp)  DFT(b3lyp)  DFT(b3lyp)  DFT(b3lyp)  DFT(b3lyp)  DFT(b3lyp) |
| **5**  **5**  **5**  **5**  **5**  **5**  **5**  **5**  **5**  **5** | 0  20  40  60  80  100  120  140  160  180 | 0.161  0  0.51  2.193  3.819  3.815  2.19  0.503  0.007  0.166 | cc-pvtz(-f)  cc-pvtz(-f)  cc-pvtz(-f)  cc-pvtz(-f)  cc-pvtz(-f)  cc-pvtz(-f)  cc-pvtz(-f)  cc-pvtz(-f)  cc-pvtz(-f)  cc-pvtz(-f) | DFT(b3lyp)  DFT(b3lyp)  DFT(b3lyp)  DFT(b3lyp)  DFT(b3lyp)  DFT(b3lyp)  DFT(b3lyp)  DFT(b3lyp)  DFT(b3lyp)  DFT(b3lyp) |
| **6**  **6**  **6**  **6**  **6**  **6**  **6**  **6**  **6**  **6** | 0  20  40  60  80  100  120  140  160  180 | 1.891  0.784  0.002  0.751  1.867  1.861  0.729  0  0.782  1.889 | cc-pvtz(-f)  cc-pvtz(-f)  cc-pvtz(-f)  cc-pvtz(-f)  cc-pvtz(-f)  cc-pvtz(-f)  cc-pvtz(-f)  cc-pvtz(-f)  cc-pvtz(-f)  cc-pvtz(-f) | DFT(b3lyp)  DFT(b3lyp)  DFT(b3lyp)  DFT(b3lyp)  DFT(b3lyp)  DFT(b3lyp)  DFT(b3lyp)  DFT(b3lyp)  DFT(b3lyp)  DFT(b3lyp) |
| **7**  **7**  **7**  **7**  **7**  **7**  **7**  **7**  **7**  **7** | 0  20  40  60  80  100  120  140  160  180 | 1.505  0.574  0  0.87  2.027  2.045  0.863  0.001  0.571  1.506 | cc-pvtz(-f)  cc-pvtz(-f)  cc-pvtz(-f)  cc-pvtz(-f)  cc-pvtz(-f)  cc-pvtz(-f)  cc-pvtz(-f)  cc-pvtz(-f)  cc-pvtz(-f)  cc-pvtz(-f) | DFT(b3lyp)  DFT(b3lyp)  DFT(b3lyp)  DFT(b3lyp)  DFT(b3lyp)  DFT(b3lyp)  DFT(b3lyp)  DFT(b3lyp)  DFT(b3lyp)  DFT(b3lyp) |
| **15**  **15**  **15**  **15**  **15**  **15**  **15**  **15**  **15**  **15** | 0  20  40  60  80  100  120  140  160  180 | 6.944  3.599  0.738  0  0.3  0.311  0.004  0.546  3.264  6.878 | cc-pvtz(-f)  cc-pvtz(-f)  cc-pvtz(-f)  cc-pvtz(-f)  cc-pvtz(-f)  cc-pvtz(-f)  cc-pvtz(-f)  cc-pvtz(-f)  cc-pvtz(-f)  cc-pvtz(-f) | DFT(b3lyp)  DFT(b3lyp)  DFT(b3lyp)  DFT(b3lyp)  DFT(b3lyp)  DFT(b3lyp)  DFT(b3lyp)  DFT(b3lyp)  DFT(b3lyp)  DFT(b3lyp) |
| **23**  **23**  **23**  **23**  **23**  **23**  **23**  **23**  **23**  **23** | 0  20  40  60  80  100  120  140  160  180 | 2.733  1.246  0.002  0.377  1.332  1.347  0.425  0  1.189  2.732 | cc-pvtz(-f)  cc-pvtz(-f)  cc-pvtz(-f)  cc-pvtz(-f)  cc-pvtz(-f)  cc-pvtz(-f)  cc-pvtz(-f)  cc-pvtz(-f)  cc-pvtz(-f)  cc-pvtz(-f) | DFT(b3lyp)  DFT(b3lyp)  DFT(b3lyp)  DFT(b3lyp)  DFT(b3lyp)  DFT(b3lyp)  DFT(b3lyp)  DFT(b3lyp)  DFT(b3lyp)  DFT(b3lyp) |
| **24**  **24**  **24**  **24**  **24**  **24**  **24**  **24**  **24**  **24** | 0  20  40  60  80  100  120  140  160  180 | 6.839  3.511  0.733  0  0.35  0.375  0.012  0.487  3.083  6.844 | cc-pvtz(-f)  cc-pvtz(-f)  cc-pvtz(-f)  cc-pvtz(-f)  cc-pvtz(-f)  cc-pvtz(-f)  cc-pvtz(-f)  cc-pvtz(-f)  cc-pvtz(-f)  cc-pvtz(-f) | DFT(b3lyp)  DFT(b3lyp)  DFT(b3lyp)  DFT(b3lyp)  DFT(b3lyp)  DFT(b3lyp)  DFT(b3lyp)  DFT(b3lyp)  DFT(b3lyp)  DFT(b3lyp) |
| **25**  **25**  **25**  **25**  **25**  **25**  **25**  **25**  **25**  **25** | 0  20  40  60  80  100  120  140  160  180 | 8.172  4.218  1.008  0  0.288  0.322  0.01  0.73  3.623  8.156 | cc-pvtz(-f)  cc-pvtz(-f)  cc-pvtz(-f)  cc-pvtz(-f)  cc-pvtz(-f)  cc-pvtz(-f)  cc-pvtz(-f)  cc-pvtz(-f)  cc-pvtz(-f)  cc-pvtz(-f) | DFT(b3lyp)  DFT(b3lyp)  DFT(b3lyp)  DFT(b3lyp)  DFT(b3lyp)  DFT(b3lyp)  DFT(b3lyp)  DFT(b3lyp)  DFT(b3lyp)  DFT(b3lyp) |
| **26**  **26**  **26**  **26**  **26**  **26**  **26**  **26**  **26**  **26** | 0  20  40  60  80  100  120  140  160  180 | 9.447  4.993  1.583  0.225  0  0.012  0.255  1.322  4.361  9.4 | cc-pvtz-pp(-f)  cc-pvtz-pp(-f)  cc-pvtz-pp(-f)  cc-pvtz-pp(-f)  cc-pvtz-pp(-f)  cc-pvtz-pp(-f)  cc-pvtz-pp(-f)  cc-pvtz-pp(-f)  cc-pvtz-pp(-f)  cc-pvtz-pp(-f) | DFT(b3lyp)  DFT(b3lyp)  DFT(b3lyp)  DFT(b3lyp)  DFT(b3lyp)  DFT(b3lyp)  DFT(b3lyp)  DFT(b3lyp)  DFT(b3lyp)  DFT(b3lyp) |
| **31**  **31**  **31**  **31**  **31**  **31**  **31**  **31**  **31**  **31** | 0  20  40  60  80  100  120  140  160  180 | 2.831  1.351  0  0.269  1.308  1.733  1.108  0.705  1.854  3.333 | cc-pvtz(-f)  cc-pvtz(-f)  cc-pvtz(-f)  cc-pvtz(-f)  cc-pvtz(-f)  cc-pvtz(-f)  cc-pvtz(-f)  cc-pvtz(-f)  cc-pvtz(-f)  cc-pvtz(-f) | DFT(b3lyp)  DFT(b3lyp)  DFT(b3lyp)  DFT(b3lyp)  DFT(b3lyp)  DFT(b3lyp)  DFT(b3lyp)  DFT(b3lyp)  DFT(b3lyp)  DFT(b3lyp) |
| **32**  **32**  **32**  **32**  **32**  **32**  **32**  **32**  **32**  **32** | 0  20  40  60  80  100  120  140  160  180 | 7.471  6.878  6.426  7.101  7.895  7.247  4.995  2.33  0.557  0 | cc-pvtz(-f)  cc-pvtz(-f)  cc-pvtz(-f)  cc-pvtz(-f)  cc-pvtz(-f)  cc-pvtz(-f)  cc-pvtz(-f)  cc-pvtz(-f)  cc-pvtz(-f)  cc-pvtz(-f) | DFT(b3lyp)  DFT(b3lyp)  DFT(b3lyp)  DFT(b3lyp)  DFT(b3lyp)  DFT(b3lyp)  DFT(b3lyp)  DFT(b3lyp)  DFT(b3lyp)  DFT(b3lyp) |
| **42** | 0 | 7.351686 | cc-pvtz(-f) | DFT(b3lyp) |
| **42** | 20 | 7.029949 | cc-pvtz(-f) | DFT(b3lyp) |
| **42** | 40 | 7.040587 | cc-pvtz(-f) | DFT(b3lyp) |
| **42** | 60 | 7.895486 | cc-pvtz(-f) | DFT(b3lyp) |
| **42** | 80 | 8.708973 | cc-pvtz(-f) | DFT(b3lyp) |
| **42** | 100 | 7.886998 | cc-pvtz(-f) | DFT(b3lyp) |
| **42** | 120 | 5.407367 | cc-pvtz(-f) | DFT(b3lyp) |
| **42** | 140 | 2.661321 | cc-pvtz(-f) | DFT(b3lyp) |
| **42** | 160 | 0.681038 | cc-pvtz(-f) | DFT(b3lyp) |
| **42** | 180 | 0 | cc-pvtz(-f) | DFT(b3lyp) |
| **44** | 0 | 5.265324 | cc-pvtz(-f) | DFT(b3lyp) |
| **44** | 20 | 4.137904 | cc-pvtz(-f) | DFT(b3lyp) |
| **44** | 40 | 3.016876 | cc-pvtz(-f) | DFT(b3lyp) |
| **44** | 60 | 2.832477 | cc-pvtz(-f) | DFT(b3lyp) |
| **44** | 80 | 3.222141 | cc-pvtz(-f) | DFT(b3lyp) |
| **44** | 100 | 2.957982 | cc-pvtz(-f) | DFT(b3lyp) |
| **44** | 120 | 1.806889 | cc-pvtz(-f) | DFT(b3lyp) |
| **44** | 140 | 0.545514 | cc-pvtz(-f) | DFT(b3lyp) |
| **44** | 160 | 0 | cc-pvtz(-f) | DFT(b3lyp) |
| **44** | 180 | 0.180905 | cc-pvtz(-f) | DFT(b3lyp) |
| **45**  **45**  **45**  **45**  **45**  **45**  **45**  **45**  **45**  **45** | 0  20  40  60  80  100  120  140  160  180 | 0  0.541  2.109  4.138  5.581  5.527  3.949  1.969  0.496  0.001 | cc-pvtz(-f)  cc-pvtz(-f)  cc-pvtz(-f)  cc-pvtz(-f)  cc-pvtz(-f)  cc-pvtz(-f)  cc-pvtz(-f)  cc-pvtz(-f)  cc-pvtz(-f)  cc-pvtz(-f) | DFT(b3lyp)  DFT(b3lyp)  DFT(b3lyp)  DFT(b3lyp)  DFT(b3lyp)  DFT(b3lyp)  DFT(b3lyp)  DFT(b3lyp)  DFT(b3lyp)  DFT(b3lyp) |
| **46** | 0 | 7.169479 | cc-pvtz(-f) | DFT(b3lyp) |
| **46** | 20 | 5.857237 | cc-pvtz(-f) | DFT(b3lyp) |
| **46** | 40 | 6.426921 | cc-pvtz(-f) | DFT(b3lyp) |
| **46** | 60 | 7.073685 | cc-pvtz(-f) | DFT(b3lyp) |
| **46** | 80 | 7.15263 | cc-pvtz(-f) | DFT(b3lyp) |
| **46** | 100 | 6.086282 | cc-pvtz(-f) | DFT(b3lyp) |
| **46** | 120 | 3.831731 | cc-pvtz(-f) | DFT(b3lyp) |
| **46** | 140 | 1.557378 | cc-pvtz(-f) | DFT(b3lyp) |
| **46** | 160 | 0 | cc-pvtz(-f) | DFT(b3lyp) |
| **46** | 180 | 0.241454 | cc-pvtz(-f) | DFT(b3lyp) |
| **47** | 0 | 3.534475 | cc-pvtz(-f) | DFT(b3lyp) |
| **47** | 20 | 3.53196 | cc-pvtz(-f) | DFT(b3lyp) |
| **47** | 40 | 3.921453 | cc-pvtz(-f) | DFT(b3lyp) |
| **47** | 60 | 4.644052 | cc-pvtz(-f) | DFT(b3lyp) |
| **47** | 80 | 5.200438 | cc-pvtz(-f) | DFT(b3lyp) |
| **47** | 100 | 4.547122 | cc-pvtz(-f) | DFT(b3lyp) |
| **47** | 120 | 2.996514 | cc-pvtz(-f) | DFT(b3lyp) |
| **47** | 140 | 1.435697 | cc-pvtz(-f) | DFT(b3lyp) |
| **47** | 160 | 0.304533 | cc-pvtz(-f) | DFT(b3lyp) |
| **47** | 180 | 0 | cc-pvtz(-f) | DFT(b3lyp) |
| **49** | 0 | 10.710103 | cc-pvtz(-f) | DFT(b3lyp) |
| **49** | 20 | 9.45325 | cc-pvtz(-f) | DFT(b3lyp) |
| **49** | 40 | 9.014258 | cc-pvtz(-f) | DFT(b3lyp) |
| **49** | 60 | 9.238873 | cc-pvtz(-f) | DFT(b3lyp) |
| **49** | 80 | 9.472443 | cc-pvtz(-f) | DFT(b3lyp) |
| **49** | 100 | 8.457581 | cc-pvtz(-f) | DFT(b3lyp) |
| **49** | 120 | 5.93886 | cc-pvtz(-f) | DFT(b3lyp) |
| **49** | 140 | 2.993026 | cc-pvtz(-f) | DFT(b3lyp) |
| **49** | 160 | 0.748132 | cc-pvtz(-f) | DFT(b3lyp) |
| **49** | 180 | 0 | cc-pvtz(-f) | DFT(b3lyp) |
| **50** | 0 | 6.836167 | cc-pvtz(-f) | DFT(b3lyp) |
| **50** | 20 | 5.190938 | cc-pvtz(-f) | DFT(b3lyp) |
| **50** | 40 | 4.459623 | cc-pvtz(-f) | DFT(b3lyp) |
| **50** | 60 | 4.686366 | cc-pvtz(-f) | DFT(b3lyp) |
| **50** | 80 | 5.139618 | cc-pvtz(-f) | DFT(b3lyp) |
| **50** | 100 | 4.814349 | cc-pvtz(-f) | DFT(b3lyp) |
| **50** | 120 | 3.388368 | cc-pvtz(-f) | DFT(b3lyp) |
| **50** | 140 | 1.594644 | cc-pvtz(-f) | DFT(b3lyp) |
| **50** | 160 | 0.384232 | cc-pvtz(-f) | DFT(b3lyp) |
| **50** | 180 | 0 | cc-pvtz(-f) | DFT(b3lyp) |
