# Supplementary material for: Rapid assessment of conformational preferences in biaryl and aryl carbonyl fragments
Source: PLoS One. 2018 Mar 14;13(3):e0192974. doi: 10.1371/journal.pone.0192974 (PMC5851544; doi:10.1371/journal.pone.0192974)
Supplement: S1 File — Description of how torsional angle of the fragments were extracted from available crystal structures (CSD: ConQuest and Relibase) and tabulated data of frequency of appearance of fragment in a molecule in a defined torsional angle interval with statistical treatment of the data. (DOCX) [file pone.0192974.s004.docx]

S1 File: Experimental validation and statistical analysis of the method.

1. Description of how torsional angle values of the fragments were extracted from available crystal structures (CSD: ConQuest and Relibase)

2013 CSD software was used to investigate the existence of a molecule that contained one of the studied fragments in a determined torsional angle. *Ortho* positions that were not blocked by a specific studied substituent in the fragment were blocked by hydrogen atoms to run the searches in a single or protein ligand bound state. All data on small molecule crystal structures described in this article were derived by searching CSD version 5.35 (November 2013) + 1 update with the ConQuest 1.16 program. Fragments were sketched manually and the torsion angle of interest was selected. If nitrogen or oxygen atoms were present in the fragment, the number of their bonded atoms was fixed to the corresponding number in the fragment in order to prevent location of irrelevant chelates. The following general flags were selected in order to be in agreement with previous studies reported by Stahl and coworkers: “only organic”, “no disordered”, “no polymeric”, “3D coordinates determined”, R factor <=0.10, “no errors” and “no ions”. The list was transferred into Mercury CSC 3.3. Torsion angle (TOR1) was extracted and data was analyzed in Data Analysis Module 1.5. Torsion angle searches with ConQuest return values between 0-180º. In the case of symmetric substructures only one arbitrary torsion angle was chosen. All data for individual torsion angles that matched with the same core and the same substituents in the *ortho* positions were grouped, exported from the Data Analysis Module and organized in tables that contained the number of X-ray structures (Frequency) that had a torsion angle within each range of 30º. X-ray crystal structures that contained one of the studied fragments as part of a macrocycle which forced an undesirable conformation were removed from the list.

Conformations of protein-bound ligands were searched with Relibase +. All fragments were sketched manually in the sketcher application available and a torsion angle was added as a 3D parameter to be measured. The same definition for the torsional angle between 0-180º was used along with relaxed filters and searches in X-ray crystal structures, NMR structures and DNA structures were performed. Structures were grouped by same core and ortho substituents and in an increment of 30º.

2. Statistical Analysis of extracted data from Cambridge Crystallographic Databases (CSD)

We have considered that in each torsional angle studied interval, a **POSITIVE PREDICTION** is where all rotamers of this interval show higher energy than 4kcal/mol in the CEP, so none rotamer in this interval is predicted to exist.

In the same way, we have considered that in each torsional angle studied interval, a **POSITIVE OBSERVATION** is when none structure with the fragment in an angle higher energy than 4kcal/mol is found in CSD.

The statistical parameters considered in order to validate the non-existence of a rotamer based on its energy were:

**PPV** (Positive Predictive Value)= SUM True Positive / (SUM True Positive + SUM False Positive)

**NPV** (Negative Predictive Value)= SUM True Negative / (SUM True Negative+ SUM False Negative)

**Sensitivity**= SUM True Positive / (SUM True Positive + SUM False Negative)

**Specificity**= SUM True Negative / (SUM True Negative + SUM False Positive)

We couldn’t adequately calculate the NPV or Sensitivity because a false negative would be defined as a rotamer that was predicted to exist but wasn’t found in the CSD; however, there are many reasons why the rotamer may not be found in the CSD apart from energy inaccessibility. So, they are considered less relevant statistics for the application of this method.

**Collected data from ConQuest and calculation of True positive, True negative, False positive and False Negative**

| **Fragment** | **Dihedral Angle- Interval** | **Frequency** | **Prediction**  (No Exist-  ΔE> 4 Kcal/mol) | **True positive** (should not exist and none found) | **True negative** (should exist and some found) | **False positive** (should not exist but some found) | **False negative** (should exist but none found) |
| --- | --- | --- | --- | --- | --- | --- | --- |
| **1,2,3,4** | 0-30 | 866 | EXIST | 0 | 1 | 0 | 0 |
| **1,2,3,4** | 30-60 | 835 | EXIST | 0 | 1 | 0 | 0 |
| **1,2,3,4** | 60-90 | 7 | EXIST | 0 | 1 | 0 | 0 |
| **5, 8, 9** | 0-30 | 304 | EXIST | 0 | 1 | 0 | 0 |
| **5, 8, 9** | 30-60 | 45 | EXIST | 0 | 1 | 0 | 0 |
| **5, 8, 9** | 60-90 | 0 | EXIST | 0 | 0 | 0 | 1 |
| **6** | 0-30 | 21 | EXIST | 0 | 1 | 0 | 0 |
| **6** | 30-60 | 36 | EXIST | 0 | 1 | 0 | 0 |
| **6** | 60-90 | 0 | EXIST | 0 | 0 | 0 | 1 |
| **7** | 0-30 | 112 | EXIST | 0 | 1 | 0 | 0 |
| **7** | 30-60 | 87 | EXIST | 0 | 1 | 0 | 0 |
| **7** | 60-90 | 0 | EXIST | 0 | 0 | 0 | 1 |
| **10,11,12** | 0-30 | 20 | EXIST | 0 | 1 | 0 | 0 |
| **10,11,12** | 30-60 | 2 | EXIST | 0 | 1 | 0 | 0 |
| **10,11,12** | 60-90 | 0 | EXIST | 0 | 0 | 0 | 1 |
| **13** | 0-30 | 1 | EXIST | 0 | 1 | 0 | 0 |
| **13** | 30-60 | 0 | EXIST | 0 | 0 | 0 | 1 |
| **13** | 60-90 | 0 | EXIST | 0 | 0 | 0 | 1 |
| **15** | 0-30 | 0 | EXIST | 0 | 0 | 0 | 1 |
| **15** | 30-60 | 36 | EXIST | 0 | 1 | 0 | 0 |
| **15** | 60-90 | 9 | EXIST | 0 | 1 | 0 | 0 |
| **16** | 0-30 | 0 | NO EXIST | 1 | 0 | 0 | 0 |
| **16** | 30-60 | 0 | EXIST | 0 | 0 | 0 | 1 |
| **16** | 60-90 | 80 | EXIST | 0 | 1 | 0 | 0 |
| **17** | 0-30 | 0 | NO EXIST | 1 | 0 | 0 | 0 |
| **17** | 30-60 | 0 | EXIST | 0 | 0 | 0 | 1 |
| **17** | 60-90 | 6 | EXIST | 0 | 1 | 0 | 0 |
| **17** | 90-120 | 1 | EXIST | 0 | 1 | 0 | 0 |
| **17** | 120-150 | 0 | EXIST | 0 | 0 | 0 | 1 |
| **17** | 150-180 | 0 | NO EXIST | 1 | 0 | 0 | 0 |
| **18** | 0-30 | 0 | NO EXIST | 1 | 0 | 0 | 0 |
| **18** | 30-60 | 0 | EXIST | 0 | 0 | 0 | 1 |
| **18** | 60-90 | 7 | EXIST | 0 | 1 | 0 | 0 |
| **18** | 90-120 | 5 | EXIST | 0 | 1 | 0 | 0 |
| **18** | 120-150 | 0 | EXIST | 0 | 0 | 0 | 1 |
| **18** | 150-180 | 0 | NO EXIST | 1 | 0 | 0 | 0 |
| **19** | 0-30 | 0 | NO EXIST | 1 | 0 | 0 | 0 |
| **19** | 30-60 | 0 | NO EXIST | 1 | 0 | 0 | 0 |
| **19** | 60-90 | 16 | EXIST | 0 | 1 | 0 | 0 |
| **22** | 0-30 | 0 | NO EXIST | 1 | 0 | 0 | 0 |
| **22** | 30-60 | 0 | EXIST | 0 | 0 | 0 | 1 |
| **22** | 60-90 | 2 | EXIST | 0 | 1 | 0 | 0 |
| **23** | 0-30 | 1 | EXIST | 0 | 1 | 0 | 0 |
| **23** | 30-60 | 20 | EXIST | 0 | 1 | 0 | 0 |
| **23** | 60-90 | 0 | EXIST | 0 | 0 | 0 | 1 |
| **24** | 0-30 | 0 | EXIST | 0 | 0 | 0 | 1 |
| **24** | 30-60 | 12 | EXIST | 0 | 1 | 0 | 0 |
| **24** | 60-90 | 2 | EXIST | 0 | 1 | 0 | 0 |
| **25** | 0-30 | 0 | EXIST | 0 | 0 | 0 | 1 |
| **25** | 30-60 | 1 | EXIST | 0 | 1 | 0 | 0 |
| **25** | 60-90 | 3 | EXIST | 0 | 1 | 0 | 0 |
| **26** | 0-30 | 0 | EXIST | 0 | 0 | 0 | 1 |
| **26** | 30-60 | 3 | EXIST | 0 | 1 | 0 | 0 |
| **26** | 60-90 | 9 | EXIST | 0 | 1 | 0 | 0 |
| **27** | 0-30 | 1 | EXIST | 0 | 1 | 0 | 0 |
| **27** | 30-60 | 77 | EXIST | 0 | 1 | 0 | 0 |
| **27** | 60-90 | 6 | EXIST | 0 | 1 | 0 | 0 |
| **28** | 0-30 | 1 | EXIST | 0 | 1 | 0 | 0 |
| **28** | 30-60 | 30 | EXIST | 0 | 1 | 0 | 0 |
| **28** | 60-90 | 4 | EXIST | 0 | 1 | 0 | 0 |
| **29** | 0-30 | 1 | EXIST | 0 | 1 | 0 | 0 |
| **29** | 30-60 | 45 | EXIST | 0 | 1 | 0 | 0 |
| **29** | 60-90 | 2 | EXIST | 0 | 1 | 0 | 0 |
| **30** | 0-30 | 0 | EXIST | 0 | 0 | 0 | 1 |
| **30** | 30-60 | 0 | EXIST | 0 | 0 | 0 | 1 |
| **30** | 60-90 | 2 | EXIST | 0 | 1 | 0 | 0 |
| **31** | 0-30 | 0 | EXIST | 0 | 0 | 0 | 1 |
| **31** | 30-60 | 0 | EXIST | 0 | 0 | 0 | 1 |
| **31** | 60-90 | 0 | EXIST | 0 | 0 | 0 | 1 |
| **31** | 90-120 | 1 | EXIST | 0 | 1 | 0 | 0 |
| **31** | 120-150 | 1 | EXIST | 0 | 1 | 0 | 0 |
| **31** | 150-180 | 0 | EXIST | 0 | 0 | 0 | 1 |
| **32** | 0-30 | 4 | NO EXIST | 0 | 0 | 1 | 0 |
| **32** | 30-60 | 2 | NO EXIST | 0 | 0 | 1 | 0 |
| **32** | 60-90 | 0 | NO EXIST | 1 | 0 | 0 | 0 |
| **32** | 90-120 | 0 | NO EXIST | 1 | 0 | 0 | 0 |
| **32** | 120-150 | 13 | EXIST | 0 | 1 | 0 | 0 |
| **32** | 150-180 | 682 | EXIST | 0 | 1 | 0 | 0 |
| **33** | 0-30 | 0 | NO EXIST | 1 | 0 | 0 | 0 |
| **33** | 30-60 | 0 | NO EXIST | 1 | 0 | 0 | 0 |
| **33** | 60-90 | 0 | NO EXIST | 1 | 0 | 0 | 0 |
| **33** | 90-120 | 0 | NO EXIST | 1 | 0 | 0 | 0 |
| **33** | 120-150 | 0 | EXIST | 0 | 0 | 0 | 1 |
| **33** | 150-180 | 18 | EXIST | 0 | 1 | 0 | 0 |
| **34** | 0-30 | 1 | NO EXIST | 0 | 0 | 1 | 0 |
| **34** | 30-60 | 0 | NO EXIST | 1 | 0 | 0 | 0 |
| **34** | 60-90 | 0 | NO EXIST | 1 | 0 | 0 | 0 |
| **34** | 90-120 | 0 | EXIST | 0 | 0 | 0 | 1 |
| **34** | 120-150 | 0 | EXIST | 0 | 0 | 0 | 1 |
| **34** | 150-180 | 52 | EXIST | 0 | 1 | 0 | 0 |
| **35** | 0-30 | 0 | NO EXIST | 1 | 0 | 0 | 0 |
| **35** | 30-60 | 0 | NO EXIST | 1 | 0 | 0 | 0 |
| **35** | 60-90 | 0 | NO EXIST | 1 | 0 | 0 | 0 |
| **35** | 90-120 | 0 | NO EXIST | 1 | 0 | 0 | 0 |
| **35** | 120-150 | 0 | EXIST | 0 | 0 | 0 | 1 |
| **35** | 150-180 | 18 | EXIST | 0 | 1 | 0 | 0 |
| **38** | 0-30 | 0 | NO EXIST | 1 | 0 | 0 | 0 |
| **38** | 30-60 | 0 | NO EXIST | 1 | 0 | 0 | 0 |
| **38** | 60-90 | 0 | NO EXIST | 1 | 0 | 0 | 0 |
| **38** | 90-120 | 0 | NO EXIST | 1 | 0 | 0 | 0 |
| **38** | 120-150 | 0 | EXIST | 0 | 0 | 0 | 1 |
| **38** | 150-180 | 3 | EXIST | 0 | 1 | 0 | 0 |
| **39** | 0-30 | 0 | EXIST | 0 | 0 | 0 | 1 |
| **39** | 30-60 | 0 | EXIST | 0 | 0 | 0 | 1 |
| **39** | 60-90 | 0 | EXIST | 0 | 0 | 0 | 1 |
| **39** | 90-120 | 0 | EXIST | 0 | 0 | 0 | 1 |
| **39** | 120-150 | 3 | EXIST | 0 | 1 | 0 | 0 |
| **39** | 150-180 | 0 | EXIST | 0 | 0 | 0 | 1 |
| **40** | 0-30 | 0 | NO EXIST | 1 | 0 | 0 | 0 |
| **40** | 30-60 | 4 | EXIST | 0 | 1 | 0 | 0 |
| **40** | 60-90 | 0 | EXIST | 0 | 0 | 0 | 1 |
| **40** | 90-120 | 0 | EXIST | 0 | 0 | 0 | 1 |
| **40** | 120-150 | 1 | EXIST | 0 | 1 | 0 | 0 |
| **40** | 150-180 | 0 | EXIST | 0 | 0 | 0 | 1 |
| **41** | 0-30 | 54 | EXIST | 0 | 1 | 0 | 0 |
| **41** | 30-60 | 7 | EXIST | 0 | 1 | 0 | 0 |
| **41** | 60-90 | 0 | EXIST | 0 | 0 | 0 | 1 |
| **42** | 0-30 | 0 | NO EXIST | 1 | 0 | 0 | 0 |
| **42** | 30-60 | 0 | NO EXIST | 1 | 0 | 0 | 0 |
| **42** | 60-90 | 0 | NO EXIST | 1 | 0 | 0 | 0 |
| **42** | 90-120 | 0 | NO EXIST | 1 | 0 | 0 | 0 |
| **42** | 120-150 | 0 | EXIST | 0 | 0 | 0 | 1 |
| **42** | 150-180 | 30 | EXIST | 0 | 1 | 0 | 0 |
| **45** | 0-30 | 263 | EXIST | 0 | 1 | 0 | 0 |
| **45** | 30-60 | 0 | EXIST | 0 | 0 | 0 | 1 |
| **45** | 60-90 | 0 | NO EXIST | 1 | 0 | 0 | 0 |
| **46** | 0-30 | 1 | NO EXIST | 0 | 0 | 1 | 0 |
| **46** | 30-60 | 0 | NO EXIST | 1 | 0 | 0 | 0 |
| **46** | 60-90 | 0 | NO EXIST | 1 | 0 | 0 | 0 |
| **46** | 90-120 | 0 | EXIST | 0 | 0 | 0 | 1 |
| **46** | 120-150 | 0 | EXIST | 0 | 0 | 0 | 1 |
| **46** | 150-180 | 18 | EXIST | 0 | 1 | 0 | 0 |
| **48** | 0-30 | 148 | EXIST | 0 | 1 | 0 | 0 |
| **48** | 30-60 | 15 | EXIST | 0 | 1 | 0 | 0 |
| **48** | 60-90 | 0 | EXIST | 0 | 0 | 0 | 1 |
| **49** | 0-30 | 0 | NO EXIST | 1 | 0 | 0 | 0 |
| **49** | 30-60 | 0 | NO EXIST | 1 | 0 | 0 | 0 |
| **49** | 60-90 | 0 | NO EXIST | 1 | 0 | 0 | 0 |
| **49** | 90-120 | 0 | NO EXIST | 1 | 0 | 0 | 0 |
| **49** | 120-150 | 0 | EXIST | 0 | 0 | 0 | 1 |
| **49** | 150-180 | 20 | EXIST | 0 | 1 | 0 | 0 |
| **51** | 0-30 | 23 | EXIST | 0 | 1 | 0 | 0 |
| **51** | 30-60 | 8 | EXIST | 0 | 1 | 0 | 0 |
| **51** | 60-90 | 0 | EXIST | 0 | 0 | 0 | 1 |
| **52** | 0-30 | 0 | EXIST | 0 | 0 | 0 | 1 |
| **52** | 30-60 | 5 | EXIST | 0 | 1 | 0 | 0 |
| **52** | 60-90 | 6 | EXIST | 0 | 1 | 0 | 0 |
| **53** | 0-30 | 0 | EXIST | 0 | 0 | 0 | 1 |
| **53** | 30-60 | 2 | EXIST | 0 | 1 | 0 | 0 |
| **53** | 60-90 | 1 | EXIST | 0 | 1 | 0 | 0 |
| **53** | 90-120 | 0 | EXIST | 0 | 0 | 0 | 1 |
| **53** | 120-150 | 0 | EXIST | 0 | 0 | 0 | 1 |
| **53** | 150-180 | 0 | EXIST | 0 | 0 | 0 | 1 |
| **54** | 0-30 | 0 | EXIST | 0 | 0 | 0 | 1 |
| **54** | 30-60 | 1 | EXIST | 0 | 1 | 0 | 0 |
| **54** | 60-90 | 1 | EXIST | 0 | 1 | 0 | 0 |
| **57** | 0-30 | 247 | EXIST | 0 | 1 | 0 | 0 |
| **57** | 30-60 | 42 | EXIST | 0 | 1 | 0 | 0 |
| **57** | 60-90 | 4 | EXIST | 0 | 1 | 0 | 0 |
| **58** | 0-30 | 0 | NO EXIST | 1 | 0 | 0 | 0 |
| **58** | 30-60 | 0 | NO EXIST | 1 | 0 | 0 | 0 |
| **58** | 60-90 | 0 | NO EXIST | 1 | 0 | 0 | 0 |
| **58** | 90-120 | 0 | EXIST | 0 | 0 | 0 | 1 |
| **58** | 120-150 | 1 | EXIST | 0 | 1 | 0 | 0 |
| **58** | 150-180 | 71 | EXIST | 0 | 1 | 0 | 0 |
| **59** | 0-30 | 0 | NO EXIST | 1 | 0 | 0 | 0 |
| **59** | 30-60 | 0 | NO EXIST | 1 | 0 | 0 | 0 |
| **59** | 60-90 | 0 | NO EXIST | 1 | 0 | 0 | 0 |
| **59** | 90-120 | 0 | EXIST | 0 | 0 | 0 | 1 |
| **59** | 120-150 | 0 | EXIST | 0 | 0 | 0 | 1 |
| **59** | 150-180 | 4 | EXIST | 0 | 1 | 0 | 0 |

From this data, we derived:

**PPV = 0.913**

**Specificity = 0.947**

In conclusion, **91%** of the time if we predict the rotamer won’t exist, we can’t find it in ConQuest CSD

**95%** of the time, if it does exist, we will predict that the rotamer will exist.

**Collected data from Relibase and calculation of True positive, True negative, False positive and False Negative**

| Fragment | Dihedral Angle- Interval | Frequency | **Prediction** (No Exist-ΔE> 4 Kcal/mol) | **True positive** (should not exist and none found) | **True negative** (should exist and some found) | **False positive** (should not exist but some found) | **False negative** (should exist but none found) |
| --- | --- | --- | --- | --- | --- | --- | --- |
| **1,2,3,4** | 0-30 | 240 | EXIST | 0 | 1 | 0 | 0 |
| **1,2,3,4** | 30-60 | 361 | EXIST | 0 | 1 | 0 | 0 |
| **1,2,3,4** | 60-90 | 46 | EXIST | 0 | 1 | 0 | 0 |
| **5, 8, 9** | 0-30 | 42 | EXIST | 0 | 1 | 0 | 0 |
| **5, 8, 9** | 30-60 | 13 | EXIST | 0 | 1 | 0 | 0 |
| **5, 8, 9** | 60-90 | 1 | EXIST | 0 | 1 | 0 | 0 |
| **6** | 0-30 | 21 | EXIST | 0 | 1 | 0 | 0 |
| **6** | 30-60 | 40 | EXIST | 0 | 1 | 0 | 0 |
| **6** | 60-90 | 1 | EXIST | 0 | 1 | 0 | 0 |
| **7** | 0-30 | 18 | EXIST | 0 | 1 | 0 | 0 |
| **7** | 30-60 | 6 | EXIST | 0 | 1 | 0 | 0 |
| **7** | 60-90 | 2 | EXIST | 0 | 1 | 0 | 0 |
| **15** | 0-30 | 0 | EXIST | 0 | 0 | 0 | 1 |
| **15** | 30-60 | 3 | EXIST | 0 | 1 | 0 | 0 |
| **15** | 60-90 | 15 | EXIST | 0 | 1 | 0 | 0 |
| **16** | 0-30 | 0 | NO EXIST | 1 | 0 | 0 | 0 |
| **16** | 30-60 | 6 | EXIST | 0 | 1 | 0 | 0 |
| **16** | 60-90 | 6 | EXIST | 0 | 1 | 0 | 0 |
| **23** | 0-30 | 0 | EXIST | 0 | 0 | 0 | 1 |
| **23** | 30-60 | 38 | EXIST | 0 | 1 | 0 | 0 |
| **23** | 60-90 | 17 | EXIST | 0 | 1 | 0 | 0 |
| **24** | 0-30 | 0 | EXIST | 0 | 0 | 0 | 1 |
| **24** | 30-60 | 4 | EXIST | 0 | 1 | 0 | 0 |
| **24** | 60-90 | 1 | EXIST | 0 | 1 | 0 | 0 |
| **27** | 0-30 | 0 | EXIST | 0 | 0 | 0 | 1 |
| **27** | 30-60 | 27 | EXIST | 0 | 1 | 0 | 0 |
| **27** | 60-90 | 8 | EXIST | 0 | 1 | 0 | 0 |
| **28** | 0-30 | 0 | EXIST | 0 | 0 | 0 | 1 |
| **28** | 30-60 | 3 | EXIST | 0 | 1 | 0 | 0 |
| **28** | 60-90 | 2 | EXIST | 0 | 1 | 0 | 0 |
| **29** | 0-30 | 0 | EXIST | 0 | 0 | 0 | 1 |
| **29** | 30-60 | 0 | EXIST | 0 | 0 | 0 | 1 |
| **29** | 60-90 | 3 | EXIST | 0 | 1 | 0 | 0 |
| **32** | 0-30 | 40 | NO EXIST | 0 | 0 | 1 | 0 |
| **32** | 30-60 | 0 | NO EXIST | 1 | 0 | 0 | 0 |
| **32** | 60-90 | 0 | NO EXIST | 1 | 0 | 0 | 0 |
| **32** | 90-120 | 0 | NO EXIST | 1 | 0 | 0 | 0 |
| **32** | 120-150 | 0 | EXIST | 0 | 0 | 0 | 1 |
| **32** | 150-180 | 2 | EXIST | 0 | 1 | 0 | 0 |
| **36** | 0-30 | 0 | NO EXIST | 1 | 0 | 0 | 0 |
| **36** | 30-60 | 0 | NO EXIST | 1 | 0 | 0 | 0 |
| **36** | 60-90 | 0 | NO EXIST | 1 | 0 | 0 | 0 |
| **36** | 90-120 | 0 | NO EXIST | 1 | 0 | 0 | 0 |
| **36** | 120-150 | 0 | EXIST | 0 | 0 | 0 | 1 |
| **36** | 150-180 | 2 | EXIST | 0 | 1 | 0 | 0 |
| **38** | 0-30 | 0 | NO EXIST | 1 | 0 | 0 | 0 |
| **38** | 30-60 | 0 | NO EXIST | 1 | 0 | 0 | 0 |
| **38** | 60-90 | 0 | NO EXIST | 1 | 0 | 0 | 0 |
| **38** | 90-120 | 0 | NO EXIST | 1 | 0 | 0 | 0 |
| **38** | 120-150 | 0 | EXIST | 0 | 0 | 0 | 1 |
| **38** | 150-180 | 4 | EXIST | 0 | 1 | 0 | 0 |
| **39** | 0-30 | 0 | EXIST | 0 | 0 | 0 | 1 |
| **39** | 30-60 | 0 | EXIST | 0 | 0 | 0 | 1 |
| **39** | 60-90 | 0 | EXIST | 0 | 0 | 0 | 1 |
| **39** | 90-120 | 0 | EXIST | 0 | 0 | 0 | 1 |
| **39** | 120-150 | 2 | EXIST | 0 | 1 | 0 | 0 |
| **39** | 150-180 | 1 | EXIST | 0 | 1 | 0 | 0 |
| **41** | 0-30 | 1 | EXIST | 0 | 1 | 0 | 0 |
| **41** | 30-60 | 0 | EXIST | 0 | 0 | 0 | 1 |
| **41** | 60-90 | 0 | EXIST | 0 | 0 | 0 | 1 |
| **45** | 0-30 | 37 | EXIST | 0 | 1 | 0 | 0 |
| **45** | 30-60 | 1 | EXIST | 0 | 1 | 0 | 0 |
| **45** | 60-90 | 0 | NO EXIST | 1 | 0 | 0 | 0 |
| **48** | 0-30 | 63 | EXIST | 0 | 1 | 0 | 0 |
| **48** | 30-60 | 18 | EXIST | 0 | 1 | 0 | 0 |
| **48** | 60-90 | 7 | EXIST | 0 | 1 | 0 | 0 |
| **51** | 0-30 | 5 | EXIST | 0 | 1 | 0 | 0 |
| **51** | 30-60 | 4 | EXIST | 0 | 1 | 0 | 0 |
| **51** | 60-90 | 0 | EXIST | 0 | 0 | 0 | 1 |
| **52** | 0-30 | 0 | EXIST | 0 | 0 | 0 | 1 |
| **52** | 30-60 | 4 | EXIST | 0 | 1 | 0 | 0 |
| **52** | 60-90 | 4 | EXIST | 0 | 1 | 0 | 0 |
| **57** | 0-30 | 12 | EXIST | 0 | 1 | 0 | 0 |
| **57** | 30-60 | 11 | EXIST | 0 | 1 | 0 | 0 |
| **57** | 60-90 | 3 | EXIST | 0 | 1 | 0 | 0 |

From this data, we derived:

**PPV = 0.93**

**Specificity = 0.98**

**93%** of the time if we predict the rotamer won’t exist, we can’t find it in Relibase CSD

**98%** of the time, if it does exist, we will predict that the rotamer will exist.
