# Supplementary material for: Rapid assessment of conformational preferences in biaryl and aryl carbonyl fragments
Source: PLoS One. 2018 Mar 14;13(3):e0192974. doi: 10.1371/journal.pone.0192974 (PMC5851544; doi:10.1371/journal.pone.0192974)

**S2 File. Comparison between CEPs using simple and complex basis sets.**

Set of CEPs comparing CEPs obtained with 6-31g* or lacvp and cc-pVTZ (-f) (cc-pVTZ-pp (-f) for **26**, iodo derivative) basis sets for **1, 5-7, 23-26, 42, 44-47, 49, 50** fragments.

This file contains graphs comparing CEPs obtained with 6-31g* or lacvp and cc-pVTZ (-f) (cc-pVTZ-pp (-f) for **26**, iodo derivative) for several fragments

Comparison between CEPs obtained using DFT/B3LYP 6-31g* or cc-pVTZ (-f) for 1, 5-7 fragments


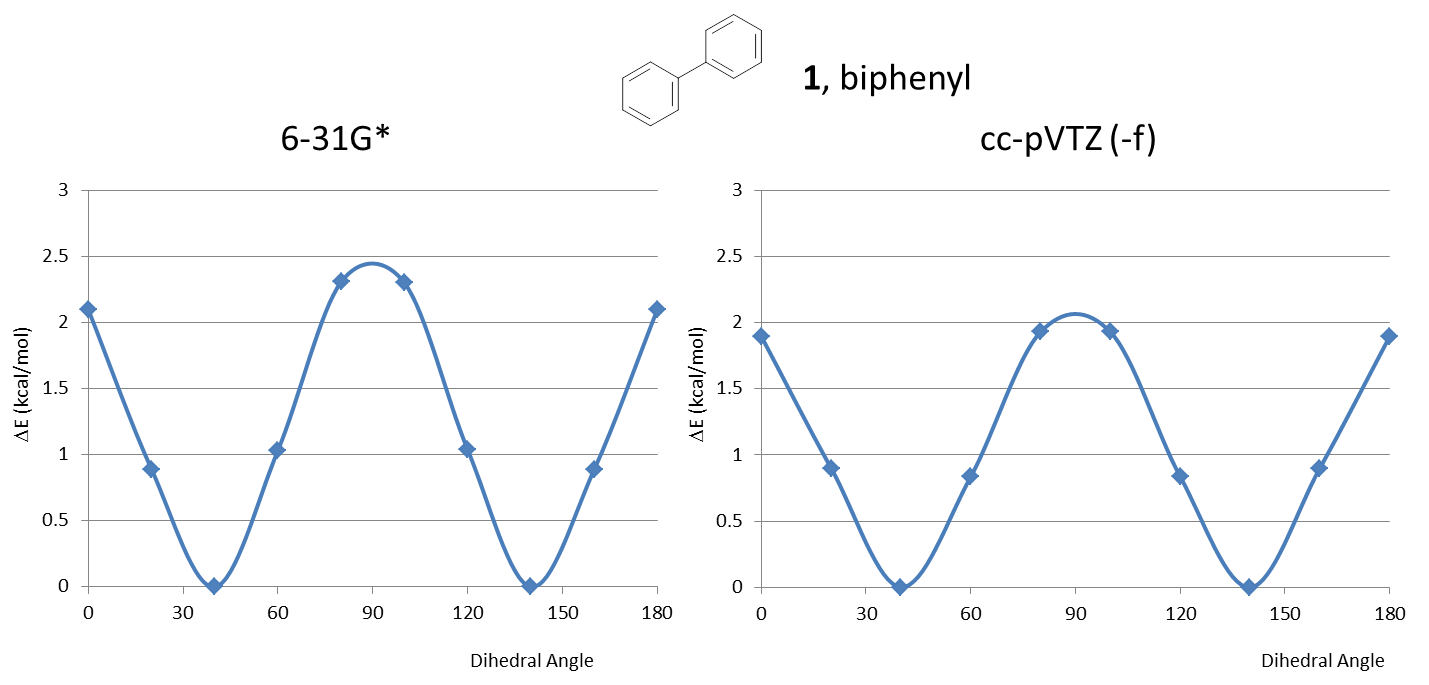


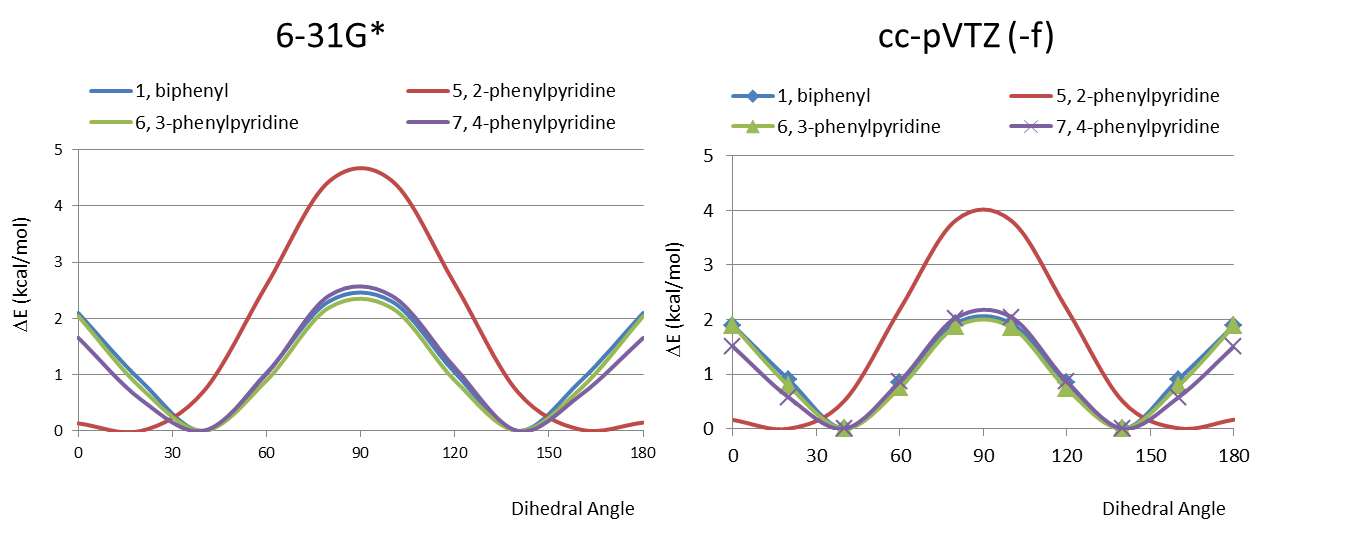


Comparison between CEPs obtained using DFT/B3LYP lacvp* and cc-pVTZ (-f) for 1, 23-26 fragments

2-iodophenyl, fragment 26, had to be calculated using cc-pVTZ-pp (-f) basis set due to the presence of iodine atom.


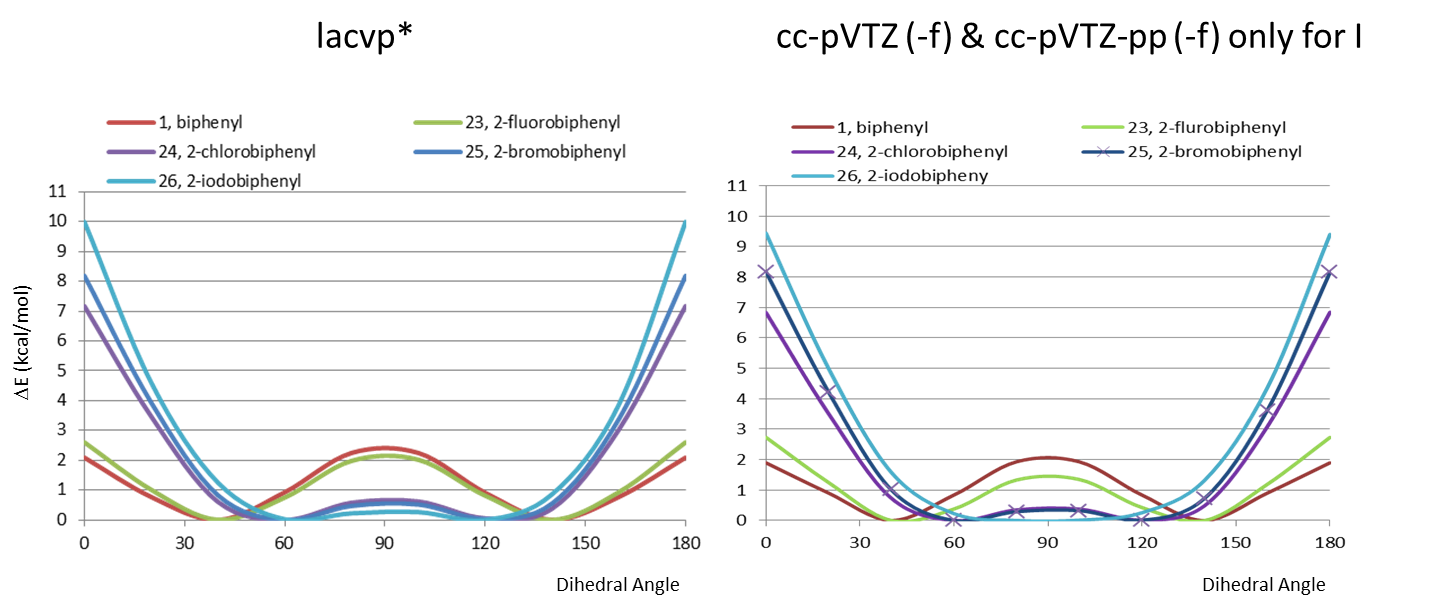


Comparison between CEPs obtained using DFT/B3LYP 6-31g* or cc-pVTZ (-f) for 42, 44, 45, 46, 47, 49, 50 fragments


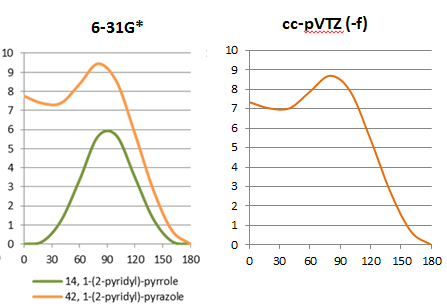


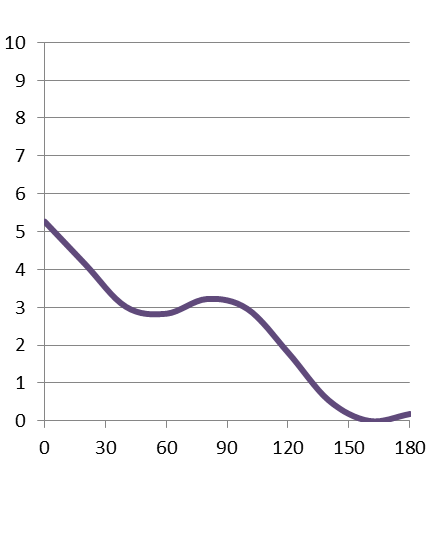


**cc-pVTZ (-f)**

**6-31G***


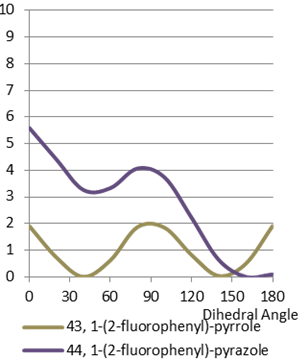


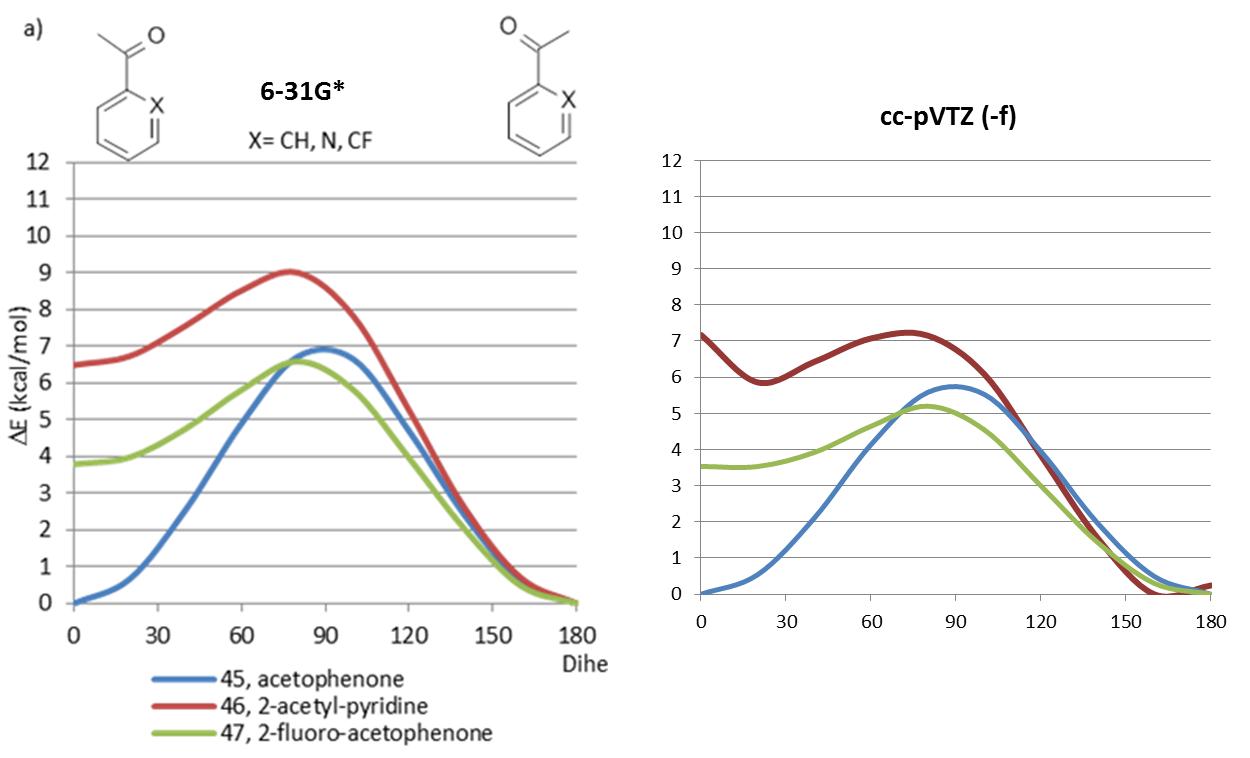


---------


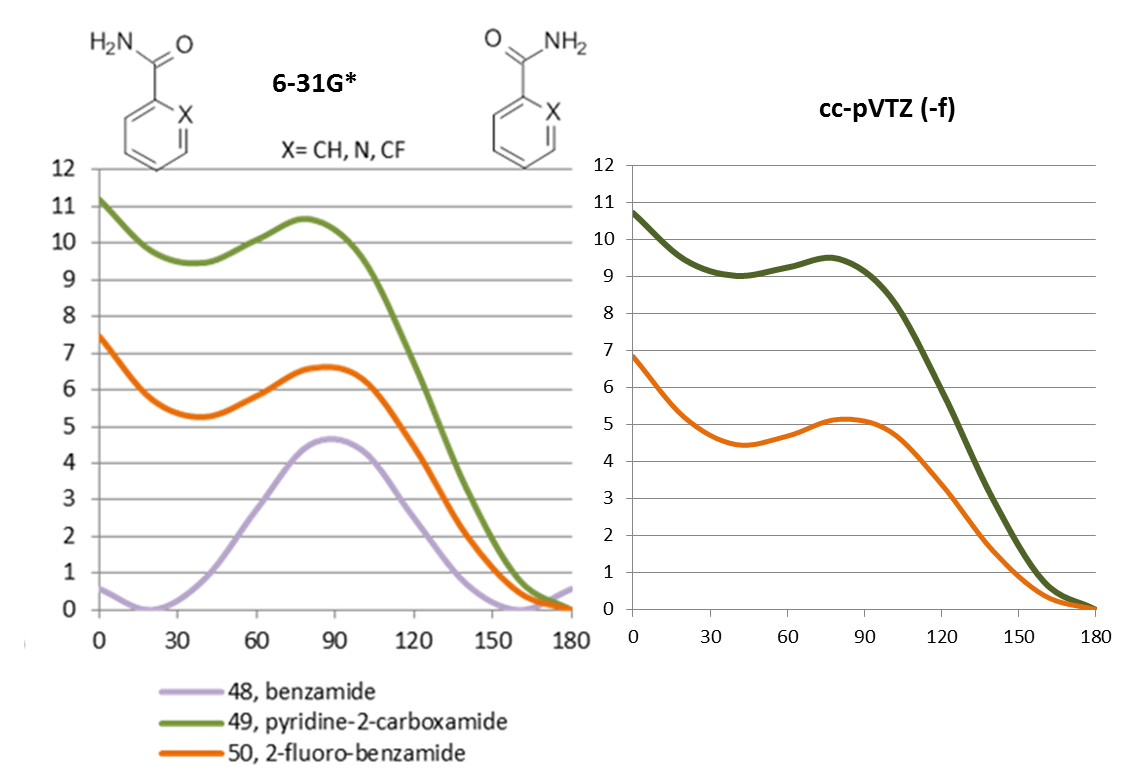

Supplement: S2 File — Set of CEPs comparing CEPs obtained with 6-31g* or lacvp and cc-pVTZ (-f) (cc-pVTZ-pp (-f) for 26, iodo derivative) basis sets for 1, 5–7, 23–26, 42, 44–47, 49, 50 fragments. (DOCX) [file pone.0192974.s005.docx]
